# Analysis of Platelet Count and New Cancer Diagnosis Over a 10-Year Period

**DOI:** 10.1001/jamanetworkopen.2021.41633

**Published:** 2022-01-11

**Authors:** Vasily Giannakeas, Joanne Kotsopoulos, Matthew C. Cheung, Laura Rosella, Jennifer D. Brooks, Lorraine Lipscombe, Mohammad R. Akbari, Peter C. Austin, Steven A. Narod

**Affiliations:** 1 Women’s College Research Institute, Women’s College Hospital, Toronto, Ontario, Canada; 2 Dalla Lana School of Public Health, University of Toronto, Toronto, Ontario, Canada; 3 ICES, Toronto, Ontario, Canada; 4 Division of Hematology, Odette Cancer Centre, Sunnybrook Health Sciences Centre, University of Toronto, Toronto, Ontario, Canada; 5 Department of Medicine, University of Toronto, Toronto, Ontario, Canada; 6 Division of Endocrinology, Women’s College Hospital, Toronto, Ontario, Canada; 7 Institute of Medical Science, University of Toronto, Toronto, Ontario, Canada; 8 Institute of Health Policy Management and Evaluation, University of Toronto, Toronto, Ontario, Canada

## Abstract

**Importance:**

Individuals with cancer often have an elevated platelet count at the time of diagnosis. The extent to which an elevated platelet count is an indicator of cancer is unclear.

**Objective:**

To evaluate the association of an elevated platelet count with a cancer diagnosis.

**Design, Setting, and Participants:**

This nested case-control study included Ontario residents enrolled in the provincial health insurance plan who had 1 or more routine complete blood count (CBC) tests performed between January 1, 2007, and December 31, 2017, with follow-up through December 31, 2018. Case patients were individuals with a new cancer diagnosis during the observation period. Eligible control individuals were cancer free before the date of diagnosis for a case patient to whom they were matched. One case patient was matched to 3 controls based on sex, age, and health care use patterns. Data were analyzed from September 24, 2020, to July 13, 2021.

**Exposures:**

Case patients and controls were assigned to 1 of 5 exposure groups based on age- and sex-specific platelet count distributions in the control population: very low (≤10th percentile), low (>10th to 25th percentile), medium (>25th to <75th percentile), high (75th to <90th percentile), and very high (≥90th percentile).

**Main Outcomes and Measures:**

Odds ratios (ORs) were estimated for specific cancer sites for each category of platelet count at intervals up to 10 years after a blood test.

**Results:**

Of the 8 917 187 eligible Ontario residents with a routine CBC record available, 4 971 578 (55.8%) were women; the median age at the first CBC was 46.4 years (IQR, 32.5-59.5 years). Among individuals with a routine CBC record available, 495 341 (5.6%) received a diagnosis of first primary cancer during the 10-year observation period. The OR for a solid tumor diagnosis associated with a very high platelet count vs a medium platelet count in the 6-month period before the diagnosis was 2.32 (95% CI, 2.28-2.35). A very high platelet count was associated with colon (OR, 4.38; 95% CI, 4.22-4.54), lung (OR, 4.37; 95% CI, 4.22-4.53), ovarian (OR, 4.62; 95% CI, 4.19-5.09), and stomach (OR, 4.27; 95% CI, 3.91-4.66) cancers. Odds ratios attenuated with increasing time from CBC test to cancer diagnosis.

**Conclusions and Relevance:**

In this nested case-control study, an elevated platelet count was associated with increased risk of cancer at several sites. Our findings suggest that an elevated platelet count could potentially serve as a marker for the presence of some cancer types.

## Key Points

**Question:**

Is a high platelet count associated with an increased risk of cancer?

**Findings:**

In this nested case-control study of 8 917 187 Ontario residents who had 1 or more routine complete blood count tests performed, an elevated platelet count was associated with a diagnosis of cancer within 10 years after the blood test. The magnitude of the association varied by cancer type and time elapsed since the blood test.

**Meaning:**

The findings suggest that a high platelet count is associated with increased cancer risk.

## Teaser

This case-control study evaluates the association of an elevated platelet count with risk of cancer by cancer type and time from a complete blood count test to cancer diagnosis.

## Introduction

Patients with cancer often have an abnormally high platelet count at the time of diagnosis (thrombocytosis), defined as a platelet count greater than 450 × 10^9^/L (to convert to ×10^3^ per microliter, divide by 1.0).^1^ A normal platelet count falls between 150 and 450 × 10^9^/L and varies with the age and sex of the individual.^1,2^ Several conditions that commonly cause an elevated platelet count include acute blood loss, infection, and inflammation.^3^ Solid tumor cancers can sometimes lead to an elevated platelet count to the extent that an undiagnosed cancer is often considered in the diagnostic workup of a patient with thrombocytosis.^3^ Cancer is believed to induce platelet formation through the release of interleukin 6, a proinflammatory cytokine that stimulates the production of thrombopoietin hormone.^4^ Elevated levels of thrombopoietin have a direct effect on increased platelet production. Excess levels of thrombopoietin in the blood stimulate megakaryocyte cell division in the bone marrow, which in turn leads to platelet formation.^5^

An elevated platelet count has been shown to be associated with short-term risk of cancer in the general population.^6,7,8^ Prospective studies evaluating platelet count and survival among patients with newly diagnosed cancer have also noted a high proportion of patients who presented with thrombocytosis.^9,10,11,12^ The excess risk associated with an elevated platelet count varies by cancer site but has been most studied for lung, colon, and gastric cancers. The full range of cancers associated with a high platelet count and whether risks associated with platelet counts within the high-normal range exist remain unclear. Furthermore, it is unclear whether the association between a high platelet count and cancer is transient or prolonged. Based on results of previous studies,^6,7,8^ a high platelet count may be a risk factor for developing cancer or, alternatively, a marker indicative of an undetected cancer. It is also not clear whether an increasing platelet count is a better indicator of a new cancer than is a high but steady platelet count.

We identified a cohort of adult residents in Ontario, Canada, who had 1 or more routine blood tests performed for a complete blood count (CBC) including platelet counts and subsequently received a diagnosis of cancer to assess the range of cancers associated with a high platelet count. We also examined whether an increasing platelet count is associated with an increased cancer risk.

## Methods

### Study Design, Population, and Data

Ontario is the most populous province in Canada, with a population of 14.5 million. Ontario residents are covered under the universal health insurance program, which includes coverage for primary care services, emergency visits, hospitalizations, and (among older adults) medication. This nested case-control study used data from ICES, a nonprofit organization that provides researchers with deidentified data that can be used for research purposes. ICES is a prescribed entity under §45 of Ontario’s Personal Health Information Protection Act, which allows for research conduct without a research ethics board review and without the need for informed consent. This study followed the Strengthening the Reporting of Observational Studies in Epidemiology (STROBE) reporting guideline for case-control studies.

ICES data include results of laboratory tests conducted in Ontario from January 2007 to present. The Ontario Laboratory Information System data set includes more than 85 million CBC test records, including those for 9.5 million (of the 14.5 million) Ontario residents. The CBC records include the date of laboratory analysis, the platelet count, and other standard blood parameters.

Incident cancers in Ontario are recorded in the Ontario Cancer Registry, which was started in January 1964. This study also used data on physician billing (Ontario Health Insurance Plan claims database), emergency department visits (National Ambulatory Care and Reporting System database), acute care hospitalizations (Discharge Abstract Database), and dispensed medications among adults aged 65 years or older (Ontario Drug Benefit Claims database). These datasets were linked using unique encoded identifiers and analyzed at ICES.

### Construction of the Cohort

The nesting cohort consisted of 8 917 187 Ontario residents who had at least 1 routine CBC test ordered by a practicing physician in a community health setting from January 1, 2007, through December 31, 2017. Cohort entry date was the date of the first eligible CBC test. Patients with cancer before the cohort entry date were excluded. Patients were observed from the date of their first routine blood test to the first date of any cancer diagnosis, death from any cause, end of Ontario Health Insurance Plan eligibility, or the end of the observation period (December 31, 2018) (eFigure 1 in the Supplement). Details on the inclusion criteria, exclusion criteria, and the study cohort are available in eTables 1-3 in the Supplement.

### Baseline Variables

Baseline information was obtained and updated at the time of each CBC test. We included information on general demographic characteristics, health services use, comorbidities and chronic conditions, medication use (among individuals aged ≥66 years), and additional CBC test results. The Johns Hopkins ACG System software, version 10,^13^ was used to obtain aggregate diagnosis groups and resource utilization bands.

### Case Patients

Case patients were defined as individuals who received a cancer diagnosis after the date of cohort entry. Data on first primary cancer diagnoses during the observation period were captured from the Ontario Cancer Registry. The Ontario Cancer Registry is a validated cancer registry that provides information on the date of diagnosis, cancer site, and tumor-specific data, such as morphologic features, stage, grade, lymph node involvement, and for certain cancers, hormone receptor status.^14^ We restricted the interpretation of our findings to solid cancers other than liver cancer because both liver and hematologic cancers may have a direct effect on platelet count; thrombopoietin production occurs in the liver, and megakaryocyte production occurs in the bone marrow. For case patients, the index date was defined as the date of diagnosis of cancer.

### Matching

We hard-matched 3 control individuals to each case patient. Each matched control was alive and cancer free on the date of diagnosis of the case patient (eFigure 1 in the Supplement). Case patients and controls were matched based on sex, calendar date of CBC test (±30 days), age (±2 years), years of coverage by the Ontario Health Insurance Plan (±2 years), and the patient’s resource utilization band. Incidence density sampling was used such that case patients could serve as potential controls at prior time points.

### Exposure

We assigned a categorical value to each platelet count based on the percentile distribution for the cancer-free controls. Five mutually exclusive categories were created: very low (≤10th percentile), low (>10th to 25th percentile), medium (>25th to <75th percentile), high (75th to <90th percentile), and very high (≥90th percentile). To account for variation in platelet count by sex and age, we defined categories of platelet count using reference distributions that were standardized according to age and sex from the pool of control patients (eFigure 2 in the Supplement).

### Statistical Analysis

#### Primary Analysis

We performed a series of (nested) matched case-control analyses to measure the association of platelet count with risk of cancer at various time intervals before the index date. Each matched quadruplet of case patients and controls (3:1) was assessed at 7 time intervals before the index date: 0 to 6 months, 6 to 12 months, 12 to 18 months, 18 to 24 months, 2 to 3 years, 3 to 5 years, and 5 to 10 years. Each case patient could contribute to up to 7 observations (1 for each time interval). If multiple routine CBC tests were performed within a given period, 1 was chosen at random. For each cancer site and for each time point, an odds ratio (OR) was estimated using conditional logistic regression. At each time point, the medium platelet count category was used as the reference group and ORs were estimated for very high, high, low, and very low counts compared with the reference group. Information on tumor stage was available for several sites. A subanalysis was conducted after stratifying cancers by stage for these sites. In a thrombocytosis sensitivity analysis, we assigned platelet levels using clinical cutoffs: thrombocytopenia (<150 ×10^9^ platelets/L), normal level (150 to 450 ×10^9^ platelets/L), and thrombocytosis (>450 ×10^9^ platelets/L).

#### Secondary Analysis

As a secondary objective, we sought to assess whether a change in platelet count over time was associated with a diagnosis of cancer. To study this, we selected individuals who had 2 routine CBC tests recorded 9 to 15 months apart. If multiple CBC records were available in the 9- to 15-month period, 1 was selected at random. A second iteration of matching (using the same matching criteria) was done using this subset of individuals (n = 4 372 288 [49% of the primary cohort]). A difference in platelet count was measured by subtracting the first platelet count from the second platelet count. Sex- and age-standardized reference distributions for change in platelet count were created (eFigure 3 in the Supplement). Five categories were created to classify the change in platelet count: large decrease (≤10th percentile), small decrease (>10th to 25th percentile), no substantial change (>25 to <75th percentile [reference group]), small increase (75th to <90th percentile), and large increase (≥90th percentile). All statistical analyses were performed using SAS software, version 9.4 (SAS Institute Inc). Data were analyzed from September 24, 2020, to July 13, 2021.

## Results

Of the 8 917 187 eligible individuals with 1 or more routine CBC tests identified in Ontario during the accrual period, 4 971 578 (55.8%) were women; the median age at the first CBC was 46.4 years (IQR, 32.5-59.5 years) (Table 1). Of the entire cohort, 495 341 individuals (5.6%) received a cancer diagnosis during the observation period. We successfully matched 491 779 case patients with cancer (99.3%) to 3 controls in 1 or more predefined time intervals. Case patients were similar to controls with respect to their demographic information, health services use, medication use, and comorbidity variables (eTable 4 in the Supplement).

**Table 1.** Characteristics of the Study Cohort at the First Eligible Routine CBC Test

Description	Individuals (N = 8 917 187)^a^
Sex	
Female	4 971 578 (55.8)
Male	3 945 609 (44.2)
Age, y	
Mean (SD)	47.0 (17.8)
Median (IQR)	46.4 (32.5-59.5)
Residence location	
Urban	8 084 848 (90.7)
Rural	820 419 (9.2)
Missing	11 920 (0.1)
Core primary care visits to general practitioner or family practitioner in previous 2 y, No.	
Mean (SD)	2.8 (3.4)
Median (IQR)	2 (1-3)
Rostered to family physician	6 940 867 (77.8)
Comorbidities and chronic conditions	
Asthma	796 810 (8.9)
Congestive heart failure	172 576 (1.9)
COPD	192 482 (2.2)
Hypertension	2 139 804 (24.0)
Diabetes	666 477 (7.5)
Kidney disease	88 520 (1.0)
Chronic coronary syndrome	321 278 (3.6)
Hemoglobin concentration, g/L	
Mean (SD)	139.5 (14.9)
Median (IQR)	140 (130-150)
Platelet count, 10^9^/L	
Mean (SD)	247.2 (64.5)
Median (IQR)	241 (205-282)
Observation time, y^b^	
Mean (SD)	6.8 (3.0)
Median (IQR)	7.3 (4.4-9.3)
Routine CBC tests in observation period, No.	
Mean (SD)	4.3 (6.3)
Median (IQR)	3 (1-6)
Cancer diagnosis^b^^,^^c^	
Any	492 691 (5.5)
Solid tumor	429 222 (4.8)
Colon	51 521 (0.6)
Lung	56 724 (0.6)
Breast^d^	65 721 (1.3)
Ovary^d^	7661 (0.2)
Cervical^d^	3494 (0.1)
Endometrial^d^	17 101 (0.3)
Prostate^e^	62 946 (1.6)
Thyroid	21 478 (0.2)
Pancreas	12 021 (0.1)
Stomach	9195 (0.1)
Kidney	14 063 (0.2)
Bladder	23 344 (0.3)
Liver	7696 (0.1)
Esophagus	4712 (0.1)
Other gastrointestinal tract	5255 (0.1)
Brain	5724 (0.1)
Melanoma	20 192 (0.2)
Head and neck	13 363 (0.1)
Other	27 011 (0.3)
Hematologic tumor	63 469 (0.7)
Leukemia	5154 (0.1)
Lymphoma	33 827 (0.4)
Multiple myeloma	8274 (0.1)
Other	16 214 (0.2)

Abbreviations: CBC, complete blood count; COPD, chronic obstructive pulmonary disease; OHIP, Ontario Health Insurance Plan.

SI conversion factors: To convert hemoglobin concentration to grams per deciliter, divide by 10.0; platelet count to 10^3^ per microliter, divide by 1.0.

^a^ Data are presented as number (percentage) of individuals unless otherwise indicated.

^b^ Period of observation was from the first eligible CBC test to the earliest date of death, end of OHIP eligibility, or end of the observation period (December 31, 2018).

^c^ Data are from the Ontario Cancer Registry.

^d^ Women only.

^e^ Men only.

The mean platelet count at the most recent blood test was higher among case patients with cancer than among matched controls (245.7 × 10^9^/L vs 237.0 × 10^9^/L). Case patients diagnosed with a solid tumor were more likely to have a recent platelet count in the highest category compared with cancer-free controls (44 344 [19.5%] vs 65 626 [9.6%]). For blood samples obtained during the 6 months before a cancer diagnosis, the OR for any solid cancer associated with a very high platelet count (≥90th percentile) vs a medium platelet count (reference, >25th to <75th percentile) was 2.32 (95% CI, 2.28-2.35) (Table 2). The OR for this association attenuated with increasing time from blood test to cancer diagnosis (Table 2); the ORs for the very high platelet category were 1.41 (95% CI, 1.39-1.44) for 6 to less than 12 months before diagnosis, 1.20 (95% CI, 1.18-1.22) for 12 to less than 24 months before diagnosis, 1.15 (95% CI, 1.13-1.17) for 24 to less than 60 months before diagnosis, and 1.13 (95% CI, 1.10-1.15) for 60 to 120 months before diagnosis.

**Table 2.** Odds Ratios of Any Solid Tumor Diagnosis by Platelet Count Category and Time From Complete Blood Count Test to Cancer Diagnosis^a^

Platelet count percentile category by time to cancer diagnosis^b^	No. (%)		Odds ratio (95% CI)^c^
	Case patients	Control individuals	
<6 mo			
Very low	19 161 (8.4)	74 891 (11.0)	0.87 (0.86-0.89)
Low	27 308 (12.0)	106 707 (15.6)	0.87 (0.86-0.89)
Medium	98 480 (43.3)	336 232 (49.2)	1 [Reference]
High	38 372 (16.9)	99 539 (14.6)	1.32 (1.30-1.34)
Very high	44 344 (19.5)	65 626 (9.6)	2.32 (2.28-2.35)
6 to <12 mo			
Very low	14 870 (10.1)	48 700 (11.0)	0.95 (0.93-0.97)
Low	20 716 (14.0)	69 561 (15.7)	0.93 (0.91-0.95)
Medium	70 078 (47.4)	219 039 (49.4)	1 [Reference]
High	23 209 (15.7)	64 288 (14.5)	1.13 (1.11-1.15)
Very high	19 038 (12.9)	42 145 (9.5)	1.41 (1.39-1.44)
12 to <24 mo			
Very low	22 086 (10.2)	70 743 (10.9)	0.95 (0.93-0.96)
Low	32 018 (14.7)	103 336 (15.9)	0.94 (0.93-0.95)
Medium	106 239 (48.9)	322 348 (49.5)	1 [Reference]
High	32 952 (15.2)	94 872 (14.6)	1.05 (1.04-1.07)
Very high	23 942 (11.0)	60 412 (9.3)	1.20 (1.18-1.22)
24 to <60 mo			
Very low	27 116 (10.1)	87 869 (10.9)	0.93 (0.92-0.95)
Low	40 852 (15.2)	127 941 (15.9)	0.97 (0.95-0.98)
Medium	132 330 (49.4)	400 393 (49.8)	1 [Reference]
High	40 363 (15.1)	115 881 (14.4)	1.05 (1.04-1.07)
Very high	27 481 (10.2)	72 342 (9.0)	1.15 (1.13-1.17)
60-120 mo			
Very low	15 744 (10.1)	49 761 (10.7)	0.96 (0.94-0.98)
Low	23 750 (15.3)	74 880 (16.1)	0.96 (0.94-0.97)
Medium	76 963 (49.5)	232 828 (49.9)	1 [Reference]
High	23 340 (15.0)	66 787 (14.3)	1.06 (1.04-1.08)
Very high	15 655 (10.1)	42 100 (9.0)	1.13 (1.10-1.15)

^a^ Liver cancer was excluded.

^b^ Platelet count percentile categories were defined as follows: very low (≤10th percentile), low (>10th to 25th percentile), medium (>25th to <75th percentile), high (75th to <90th percentile), and very high (≥90th percentile).

^c^ *P* < .001 for all.

**Figure 1.**
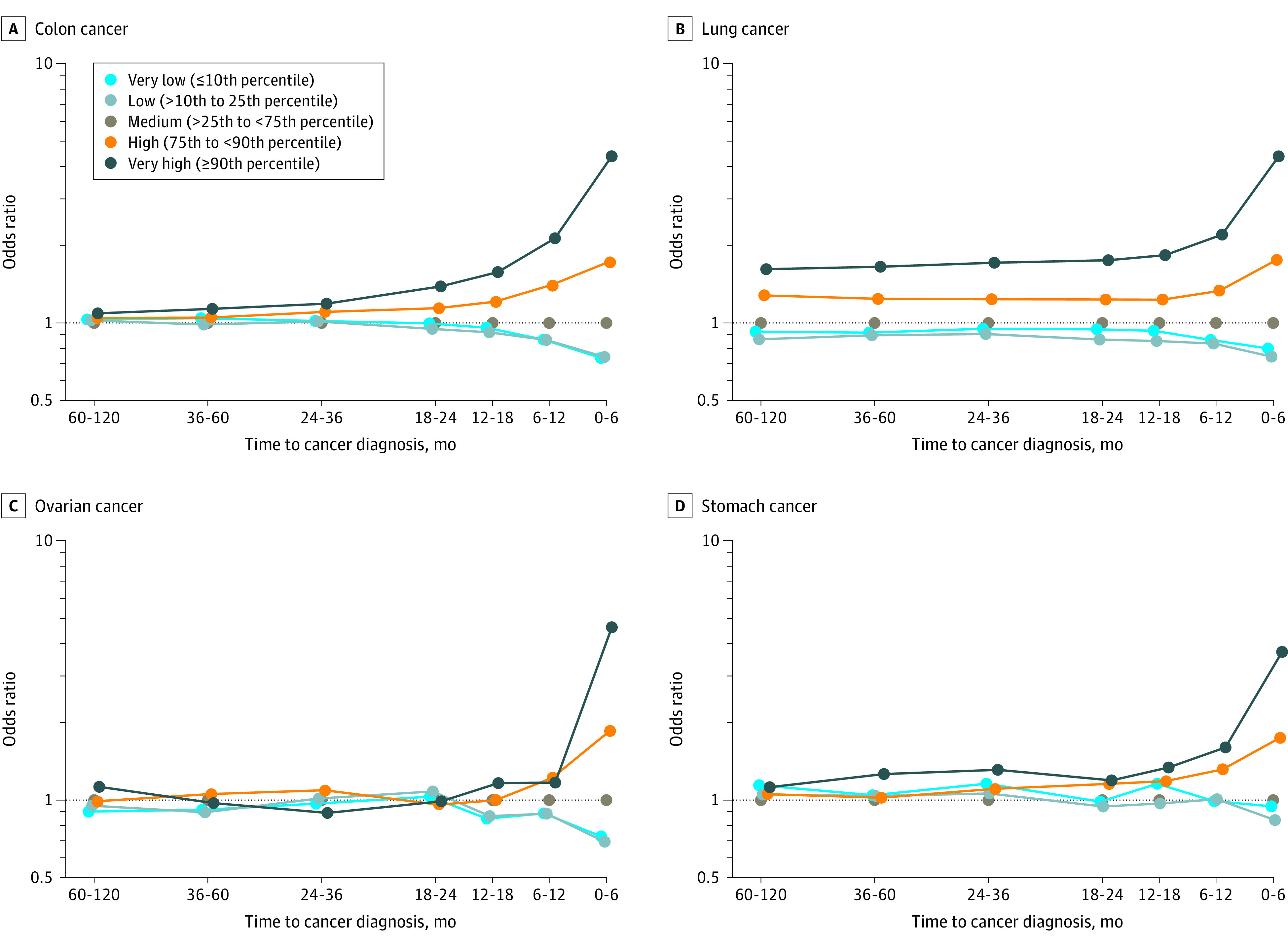
Odds Ratios of Cancer by Platelet Count Category and Time From Complete Blood Count Test to Cancer Diagnosis

The ORs for the association of a high platelet count with a cancer diagnosis were greatest for patients with cancers of the colon, lung, ovary, and stomach (Figure 1). During the 0- to 6-month period before a cancer diagnosis, the ORs for the very high platelet count category were 4.38 (95% CI, 4.22-4.54) for colon cancer, 4.37 (95% CI, 4.22-4.53) for lung cancer, 4.62 (95% CI, 4.19-5.09) for ovarian cancer, and 4.27 (95% CI, 3.91-4.66) for stomach cancer (Figure 1 and eTable 5 in the Supplement). Significant associations were also observed for esophageal cancer (OR, 3.18; 95% CI, 2.81-3.60), other gastrointestinal tract cancers (OR, 3.10; 95% CI, 2.75-3.49), and kidney cancer (OR, 2.55; 95% CI, 2.38-2.74) (eFigure 4 in the Supplement). The associations attenuated with increasing time to diagnosis to varying degrees.

In addition, high platelet count was associated with risk of breast cancer (OR, 1.05; 95% CI, 1.01-1.10) and prostate cancer (OR, 1.24; 95% CI, 1.19-1.29) but was not associated with risk of melanoma (OR, 1.06; 95% CI, 0.97-1.15) or thyroid cancer (OR, 1.01; 95% CI, 0.94-1.09) (eFigure 4 in the Supplement). A low platelet count and a very low platelet count were associated with a decreased risk of breast and prostate cancer. In a sensitivity analysis, the ORs for the association of thrombocytosis and increased cancer risk were greatest for colon cancer, lung cancer, ovarian cancer, stomach cancer, esophageal cancer, and kidney cancer (eFigure 7 in the Supplement).

**Figure 2.**
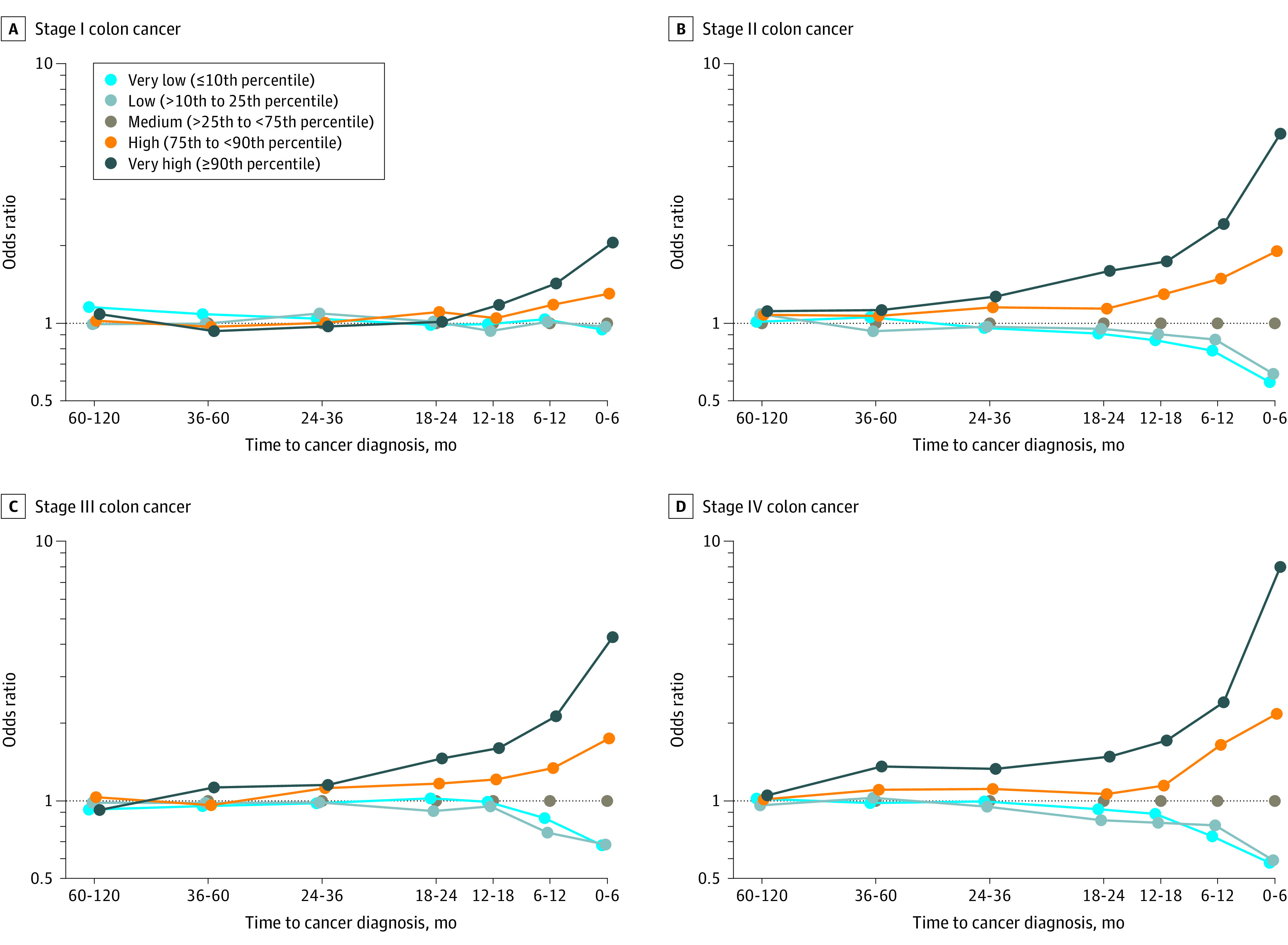
Odds Ratios of Colon Cancer by Platelet Count Category and Time From Complete Blood Count Test to Cancer Diagnosis by Cancer Stage

We also studied the associations between a high platelet count and risk of solid tumors by stage at diagnosis (when data were available). There was a significant association across all stages of colon cancer, but the OR for the association was greatest for metastatic disease (stage IV) (OR, 7.96; 95% CI, 7.26-8.72) (Figure 2). Data for the other cancer sites by stage are presented in eFigure 6 in the Supplement.

**Figure 3.**
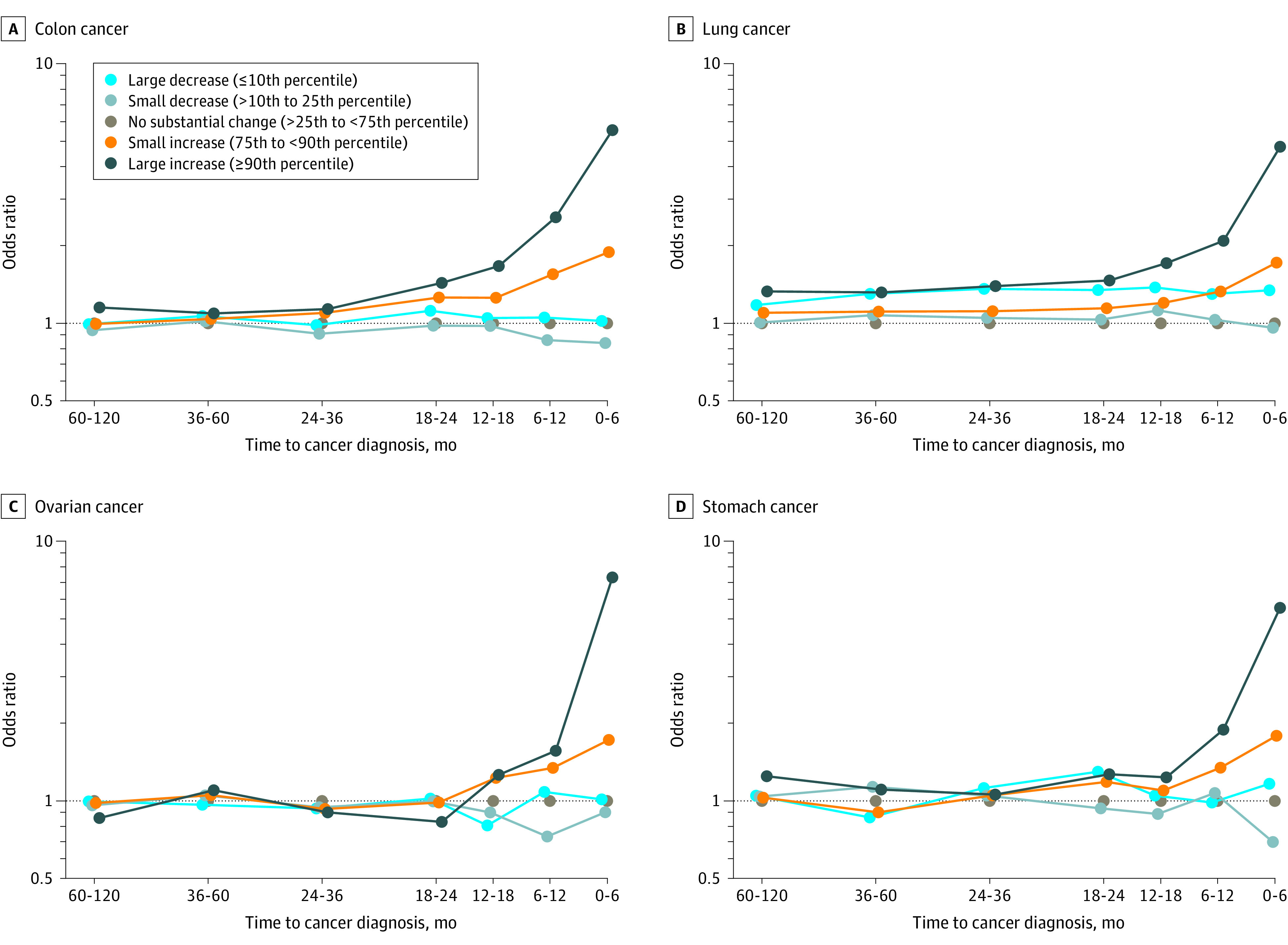
Odds Ratios of Cancer by Change in Platelet Count Category and Time From Complete Blood Count Test to Diagnosis

We also examined whether a substantial increase in platelet count (compared with a platelet count measured in the previous 9 to 15 months) was associated with an risk of cancer. Case patients diagnosed with a solid tumor were more likely to have a recent increase in platelet count (≥90th percentile) than were cancer-free controls (19 750[21.8%] vs 27 530 [10.2%]) (eTable 6 in the Supplement). A recent increase in the platelet count was associated with risk of colon cancer (OR, 5.52; 95% CI, 5.21-5.86), lung cancer (OR, 4.77; 95% CI, 4.51-5.04), ovarian cancer (OR, 7.23; 95% CI, 6.12-8.53), and stomach cancer (OR, 5.51; 95% CI, 4.82-6.29) (Figure 3 and eTable 7 in the Supplement). No associations were observed between a recent increase in the platelet count and breast cancer (OR, 1.01; 95% CI, 0.94-1.09), melanoma (OR, 1.01; 95% CI, 0.89-1.15), or thyroid cancer (OR, 0.97; 95% CI, 0.86-1.09) (eFigure 5 in the Supplement).

## Discussion

In this large, nested case-control study, we found that an elevated platelet count identified during a routine blood examination was associated with an increased risk of developing a range of solid tumors. The OR for the association was greatest for a diagnosis of cancer within 6 months of a blood test. For several cancer sites (lung, colon, stomach, esophagus, and kidney), a high platelet count was associated with a cancer diagnosis in the following 3 or more years. For lung cancer, a significant association was present 10 years before diagnosis. Long-term associations were also seen for kidney cancer and esophageal cancer. In contrast, for ovarian cancer, there was an association only in the 6 months before a diagnosis.

Overall, given the transient nature of the association with platelet count, our findings suggest that an elevated platelet count detected through routine blood examination may be a consequence of the presence of cancer rather than being a risk factor for the disease. The physiologic basis for the association is not clear but may be multifactorial. Platelets are produced in the bone marrow in response to thrombopoietin, which is upregulated by interleukin 6, primarily produced in the liver.^15^ It is possible that the increase in platelet count is a response to circulating factors produced by the cancer cells or is a local response to inflammation induced by the cancer cell mass. Various mechanisms have been proposed to explain the association between high platelet count and cancer, including the aggregation of cancer cells by platelets, increased extravasation or enhanced permeability of the basement membrane, and shielding cancer cells from immune attack in the bloodstream.^16,17,18^ Other possible mechanisms include iron deficiency, bleeding (among patients with colon cancer),^19^ abnormal platelet counts, and the infiltration of disseminated cancer cells in the bone marrow.^20^

Several studies have demonstrated an association between an elevated platelet count (thrombocytosis) and cancer risk. In general, these studies have either measured platelet counts at the time of diagnosis or had a short follow-up period subsequent to a CBC test.^6,7,8^ Pharmacoepidemiologic studies have further shown a lower incidence of certain cancer types among patients receiving platelet-inhibiting medications. For example, there is an established relationship between aspirin use (an antiplatelet drug) and decreased incidence of colon cancer.^21,22,23^ A protective effect of low-dose aspirin against ovarian cancer has also been suggested.^24^ Although antiplatelet medications inhibit platelet function as opposed to lowering the platelet count, the decreased incidence of cancer associated with aspirin use suggests the potential role functional platelets have in cancer risk.

Our study findings suggest that individuals with a high platelet count might be candidates for investigation for the presence of an occult cancer after other nonmalignant causes of an elevated platelet count have been ruled out. Of individuals who had a cancer diagnosed within 6 months after the blood test, 19.5% had a very high platelet count (top 10 percentile). In a sensitivity analysis, we observed similar findings, with an association with some cancers among individuals with thrombocytosis. Giannakeas and Narod^25^ recently reported an association between thrombocytosis and incident cancers using the same data from the present study. The findings of the present study suggest that platelet counts might be useful as a cancer screening tool alone or in combination with other cancer screening modalities, in particular spiral computed tomography for lung cancer, colonoscopy for colon cancer, and a cancer antigen 125 test or transvaginal ultrasonography for ovarian cancer. Novel screening tests that incorporate cell-free DNA and methylation signatures have shown promising results in identifying site-specific cancers.^26^ Platelet count could potentially be used as an affordable screening test to improve the predictive value of other screening modalities. Particular attention should be given to individuals who have an increasing platelet count (Figure 3). A relative increase in platelet count that exceeded 1.5 SDs (ie, ≥90th percentile) was associated with risk for many cancer types.

The associations found in this study were based on a single marker (platelet count) as a 1-time measurement and as a change over time. In future studies, we plan to investigate the clinical utility of platelet count testing as a screening test. We will incorporate additional blood count elements in combination with platelet count in a model to maximize predictive ability.

### Limitations

This study has limitations. The extent to which unmeasured confounders influenced the association of platelet count with cancer diagnosis is unclear. For lung cancer, we observed a prolonged association with elevated platelet count throughout the 10-year observation period (Figure 1). Smoking status was not available through administrative data sources. Platelet counts have been shown to differ among smokers and nonsmokers.^27^ Body mass index was also not available and has been shown to be associated with platelet counts among women.^28^ Additional variables that are likely to influence platelet count include alcohol consumption, family history, and genetics. These variables were not attainable in our study because of the limitations of administrative health data. However, given the transient association observed between platelet count and a cancer diagnosis in this study, it is unlikely that prolonged exposures would be attributed to these variables. Moreover, the secondary analysis of the change in platelet count in an individual (in whom confounders were presumed to be fixed) over time revealed findings comparable to those seen in the primary analysis.

## Conclusions

In this nested case-control study, an elevated platelet count was associated with increased risk of cancer at several sites. The association was transient and attenuated with increasing time from CBC test to the date of the cancer diagnosis. Odds ratios were greatest for colon, lung, ovary, gastroesophageal, and kidney cancers. Our findings suggest that an elevated platelet count could potentially serve as a marker for the presence of some cancer types.

## Notes

Supplement. **eTable 1.** Inclusion Table for CBC Tests **eTable 2.** Exclusion Table for Study Cohort **eTable 3.** Detailed Descriptive Table of Eligible Subjects, Measured at First Eligible Routine CBC Test (Cohort Entry Date) **eTable 4.** Descriptive Table of Matched Subjects (Primary Analysis), Variables Measured on Index CBC **eTable 5.** Odds Ratio of Cancer by Platelet Count Category and Time From Diagnosis. Select Cancer Sites **eTable 6.** Odds Ratio of Any Solid Tumour Diagnosis (Excluding Liver) by Change in Platelet Count Category and Time From Cancer Diagnosis **eTable 7.** Odds Ratio of Cancer by Change in Platelet Count Category and Time From Diagnosis. Select Cancer Sites **eFigure 1.** Study Design Criteria Among Matched Individuals **eFigure 2.** Age- and Sex-Specific Platelet Count Reference Distributions for Exposure Definition **eFigure 3.** Age- and Sex-Specific Platelet Count Reference Distributions for Secondary Exposure Definition **eFigure 4.** Odds Ratio of Cancer by Platelet Count Category and Time From Cancer Diagnosis. Additional Cancer Sites **eFigure 5.** Odds Ratio of Cancer by Change in Platelet Count Category and Time From Cancer Diagnosis. Additional Cancer Sites **eFigure 6.** Odds Ratio of Cancer by Platelet Count Category and Time From Diagnosis. Select Cancer Sites by Cancer Stage **eFigure 7.** Odds Ratio of Cancer by Platelet Count Category (Clinical Definition) and Time From Diagnosis
