# Supplementary material for: Analysis of Platelet Count and New Cancer Diagnosis Over a 10-Year Period
Source: JAMA Netw Open. 2022 Jan 11;5(1):e2141633. doi: 10.1001/jamanetworkopen.2021.41633 (PMC8753503; doi:10.1001/jamanetworkopen.2021.41633)
Supplement: Supplement. — eTable 1. Inclusion Table for CBC Tests eTable 2. Exclusion Table for Study Cohort eTable 3. Detailed Descriptive Table of Eligible Subjects, Measured at First Eligible Routine CBC Test (Cohort Entry Date) eTable 4. Descriptive Table of Matched Subjects (Primary Analysis), Variables Measured on Index CBC eTable 5. Odds Ratio of Cancer by Platelet Count Category and Time From Diagnosis. Select Cancer Sites eTable 6. Odds Ratio of Any Solid Tumour Diagnosis (Excluding Liver) by Change in Platelet Count Category and Time From Cancer Diagnosis eTable 7. Odds Ratio of Cancer by Change in Platelet Count Category and Time From Diagnosis. Select Cancer Sites eFigure 1. Study Design Criteria Among Matched Individuals eFigure 2. Age- and Sex-Specific Platelet Count Reference Distributions for Exposure Definition eFigure 3. Age- and Sex-Specific Platelet Count Reference Distributions for Secondary Exposure Definition eFigure 4. Odds Ratio of Cancer by Platelet Count Category and Time From Cancer Diagnosis. Additional Cancer Sites eFigure 5. Odds Ratio of Cancer by Change in Platelet Count Category and Time From Cancer Diagnosis. Additional Cancer Sites eFigure 6. Odds Ratio of Cancer by Platelet Count Category and Time From Diagnosis. Select Cancer Sites by Cancer Stage eFigure 7. Odds Ratio of Cancer by Platelet Count Category (Clinical Definition) and Time From Diagnosis [file jamanetwopen-e2141633-s001.pdf]

Supplementary Online Content

Giannakeas V, Kotsopoulos J, Cheung MC, et al. Analysis of platelet count and new cancer diagnosis over a 10-year period. *JAMA Netw Open*. 2022;5(1):e2141633. doi:10.1001/jamanetworkopen.2021.41633

eTable 1. Inclusion Table for CBC Tests

eTable 2. Exclusion Table for Study Cohort

eTable 3. Detailed Descriptive Table of Eligible Subjects, Measured at First Eligible Routine CBC Test (Cohort Entry Date)

eTable 4. Descriptive Table of Matched Subjects (Primary Analysis), Variables Measured on Index CBC

eTable 5. Odds Ratio of Cancer by Platelet Count Category and Time From Diagnosis. Select Cancer Sites

eTable 6. Odds Ratio of Any Solid Tumour Diagnosis (Excluding Liver) by Change in Platelet Count Category and Time From Cancer Diagnosis

eTable 7. Odds Ratio of Cancer by Change in Platelet Count Category and Time From Diagnosis. Select Cancer Sites

eFigure 1. Study Design Criteria Among Matched Individuals

eFigure 2. Age- and Sex-Specific Platelet Count Reference Distributions for Exposure Definition

eFigure 3. Age- and Sex-Specific Platelet Count Reference Distributions for Secondary Exposure Definition

eFigure 4. Odds Ratio of Cancer by Platelet Count Category and Time From Cancer Diagnosis. Additional Cancer Sites

eFigure 5. Odds Ratio of Cancer by Change in Platelet Count Category and Time From Cancer Diagnosis. Additional Cancer Sites

eFigure 6. Odds Ratio of Cancer by Platelet Count Category and Time From Diagnosis. Select Cancer Sites by Cancer Stage

eFigure 7. Odds Ratio of Cancer by Platelet Count Category (Clinical Definition) and Time From Diagnosis

This supplementary material has been provided by the authors to give readers additional information about their work.

© 2022 Giannakeas V et al. *JAMA Network Open*.

eTable 1. Inclusion table for CBC tests

	Frequency	Percent
Total CBC observations in accrual period	85,868,893	
**Exclusion**		
1. Priority of blood test not routine	14,033,973	16.34
2. Patient class not in community	15,163,171	17.66
3. Ordering practitioner not an MD	1,416,014	1.65
**Eligible CBC observations for inclusion cohort**	55,255,735	64.35

© 2022 Giannakeas V et al. *JAMA Network Open*.

eTable 2. Exclusion table for study cohort

	Frequency	Percent
Total CBC observations meeting inclusion criteria	55,255,735	
Exclusion		
1. Missing date of birth	34	0
2. Missing sex	-	0
3. Age <18 on CBC date	2,151,138	3.89
4. Age >100 on CBC date	24,801	0.04
5. Death date prior to CBC date	13,661	0.02
6. OHIP ineligible on CBC date	55,740	0.1
7. Multiple CBC tests on same day	246,020	0.44
8. History of cancer in the Ontario Cancer Registry	5,144,653	9.31
**Eligible CBC observations in study**	47,373,668	85.73
**Number of unique individuals**	8,917,187	

© 2022 Giannakeas V et al. *JAMA Network Open*.

eTable 3. Detailed descriptive table of eligible subjects, measured at first eligible routine CBC test (cohort entry date)

Description	Value	Total
Overall		8,917,187
**General demographics**		
Calendar year	Mean (SD)	2011.1 (2.7)
	Median (IQR)	2011 (2009-2013)
Sex	Female	4,971,578 (55.8%)
	Male	3,945,609 (44.2%)
Age	Mean (SD)	47.0 (17.8)
	Median (IQR)	46.4 (32.5-59.5)
Neighborhood income quintile	1 - Low	1,696,790 (19.0%)
	2	1,761,615 (19.8%)
	3	1,781,901 (20.0%)
	4	1,853,508 (20.8%)
	5 - High	1,784,803 (20.0%)
	Missing	38,570 (0.4%)
Residence location	Urban	8,084,848 (90.7%)
	Rural	820,419 (9.2%)
	Missing	11,920 (0.1%)
Landed immigrant	Non-immigrant	7,266,961 (81.5%)
	Recent immigrant (10 years or less)	742,701 (8.3%)
	Past immigrant (more than 10 years)	907,525 (10.2%)
Time eligible in OHIP (years)	Mean (SD)	11.6 (7.3)
	Median (IQR)	11.2 (5.3-17.4)
**Health service utilization**		
Core primary care visits to GP/FP (2 years prior)	Mean (SD)	2.8 (3.4)
	Median (IQR)	2 (1-3)
	0	564,943 (6.3%)
	1 - 2	4,903,886 (55.0%)
	3 - 4	1,824,321 (20.5%)
	5 - 9	1,285,001 (14.4%)
	10+	339,036 (3.8%)
ED visits (2 years prior)	0	5,817,079 (65.2%)
	1	1,672,061 (18.8%)
	2	680,835 (7.6%)
	3+	747,212 (8.4%)
Inpatient hospitalization episodes (2 years prior)	0	8,413,664 (94.4%)
	1	396,207 (4.4%)
	2	71,531 (0.8%)
	3+	35,785 (0.4%)

© 2022 Giannakeas V et al. *JAMA Network Open*.

Description	Value	Total
Rostered to family physician	Yes	6,940,867 (77.8%)
Up to date on cancer screening		
Pap smear (females age 18 to 70)	Yes	2,583,203 (59.0%)
Mammogram (females age 50 to 70)	Yes	823,747 (58.2%)
FOBT/sigmoidoscopy/colonoscopy (age 50 to 70)	Yes	1,333,036 (47.8%)
Comorbidities and chronic conditions		
Resource utilization band	0 - No or invalid diagnosis	279,791 (3.1%)
	1 - Healthy user	464,794 (5.2%)
	2 - Low user	1,653,945 (18.5%)
	3 - Moderate user	4,736,581 (53.1%)
	4 - High user	1,367,006 (15.3%)
	5 - Very high user	415,070 (4.7%)
Chronic conditions		
Asthma	Yes	796,810 (8.9%)
Congestive heart failure	Yes	172,576 (1.9%)
Inflammatory bowel disease	Yes	29,509 (0.3%)
Chronic obstructive pulmonary disease	Yes	192,482 (2.2%)
HIV	Yes	11,242 (0.1%)
Hypertension	Yes	2,139,804 (24.0%)
Dementia	Yes	113,493 (1.3%)
Diabetes	Yes	666,477 (7.5%)
Chronic rheumatoid arthritis	Yes	74,781 (0.8%)
Osteoarthritis	Yes	869,722 (9.8%)
Mood disorder	Yes	1,093,308 (12.3%)
Other mental health disorder	Yes	356,591 (4.0%)
Osteoporosis	Yes	65,463 (0.7%)
Renal disease	Yes	88,520 (1.0%)
Stroke	Yes	62,149 (0.7%)
Chronic coronary syndrome	Yes	321,278 (3.6%)
Acute myocardial infarction	Yes	83,489 (0.9%)
**Medication use (age 66+)**		
Concurrent medication use		
Number of concurrent medications	Mean (SD)	3.7 (3.1)
	Median (IQR)	3 (1-6)
Recent medication use		
Antiplatelet		
Nonsteroidal anti-inflammatory (ASA-based)	Yes	67,300 (4.8%)
Nonsteroidal anti-inflammatory (non-ASA-based)	Yes	290,436 (20.8%)
Adenosine disphosphonate inhibitor	Yes	75,213 (5.4%)
Cardiovascular		
Coronary vasodilator (nitrate)	Yes	113,508 (8.1%)
Beta blocker	Yes	360,850 (25.9%)

© 2022 Giannakeas V et al. *JAMA Network Open*.

Description	Value	Total
Calcium channel blocker	Yes	381,621 (27.4%)
ACE inhibitor	Yes	423,063 (30.4%)
Angiotensin receptor agonist	Yes	286,627 (20.6%)
Lipid-lowering		
Statin	Yes	623,653 (44.7%)
Psychotropics		
Tricyclic antidepressant	Yes	68,640 (4.9%)
Selective serotonin reuptake inhibitor	Yes	150,581 (10.8%)
**Complete blood count**		
Hemoglobin concentration [g/L]	Mean (SD)	139.5 (14.9)
	Median (IQR)	140 (130-150)
Hematocrit ratio [L/L]	Mean (SD)	0.4 (0.0)
	Median (IQR)	0.4 (0.4-0.4)
Platelet count [10^9 cells/L]	Mean (SD)	247.2 (64.5)
	Median (IQR)	241 (205-282)
**Observation period***		
Observation time (years)	Mean (SD)	6.8 (3.0)
	Median (IQR)	7.3 (4.4-9.3)
Number of routine CBC tests	Mean (SD)	4.3 (6.3)
	Median (IQR)	3 (1-6)
Incident cancer events (Ontario Cancer Registry)		
Any cancer diagnosis	Yes	492,691 (5.5%)
Solid tumour	Yes	429,222 (4.8%)
Colon	Yes	51,521 (0.6%)
Lung	Yes	56,724 (0.6%)
Breast (female only)	Yes	65,721 (1.3%)
Ovary (female only)	Yes	7,661 (0.2%)
Cervical (female only)	Yes	3,494 (0.1%)
Endometrial (female only)	Yes	17,101 (0.3%)
Prostate (male only)	Yes	62,946 (1.6%)
Thyroid	Yes	21,478 (0.2%)
Pancreas	Yes	12,021 (0.1%)
Stomach	Yes	9,195 (0.1%)
Kidney	Yes	14,063 (0.2%)
Bladder	Yes	23,344 (0.3%)
Liver	Yes	7,696 (0.1%)
Esophagus	Yes	4,712 (0.1%)
Other GI	Yes	5,255 (0.1%)
Brain	Yes	5,724 (0.1%)
Melanoma	Yes	20,192 (0.2%)
Head and neck	Yes	13,363 (0.1%)
Other solid tumour	Yes	27,011 (0.3%)
Hematologic tumour	Yes	63,469 (0.7%)

© 2022 Giannakeas V et al. *JAMA Network Open*.

Description	Value	Total
Leukemia	Yes	5,154 (0.1%)
Lymphoma	Yes	33,827 (0.4%)
Multiple myeloma	Yes	8,274 (0.1%)
Other hematologic tumour	Yes	16,214 (0.2%)

*Period of observation is from first eligible CBC to the earliest date for: death, end of OHIP eligibility, or end of observation period (December 31 2018)

© 2022 Giannakeas V et al. *JAMA Network Open*.

eTable 4. Descriptive table of matched subjects (primary analysis), variables measured on index CBC.

Description	Value	Total	Case	Control	Standardized Difference
Overall		5,677,304	1,419,326 (25.0%)	4,257,978 (75.0%)	
General demographics					
Calendar year (cont.)	Mean (SD)	2012.2 (2.6)	2012.2 (2.6)	2012.2 (2.6)	
	Median (IQR)	2012 (2010-2014)	2012 (2010-2014)	2012 (2010-2014)	
Calendar year (cat.)	2007-2009	1,091,243 (19.2%)	272,743 (19.2%)	818,500 (19.2%)	0
	2010-2012	2,020,199 (35.6%)	505,028 (35.6%)	1,515,171 (35.6%)	0
	2013-2015	1,777,747 (31.3%)	444,546 (31.3%)	1,333,201 (31.3%)	0
	2016-2017	788,115 (13.9%)	197,009 (13.9%)	591,106 (13.9%)	0
Sex	Female	2,885,112 (50.8%)	721,278 (50.8%)	2,163,834 (50.8%)	0
	Male	2,792,192 (49.2%)	698,048 (49.2%)	2,094,144 (49.2%)	0
Age (cont.)	Mean (SD)	65.9 (13.6)	66.0 (13.6)	65.9 (13.6)	0
	Median (IQR)	67.0 (57.4-75.9)	67.0 (57.4-76.0)	67.1 (57.4-75.9)	
Age (cat.)	18-29	66,388 (1.2%)	16,491 (1.2%)	49,897 (1.2%)	0
	30-39	172,563 (3.0%)	43,121 (3.0%)	129,442 (3.0%)	0
	40-49	468,186 (8.2%)	116,702 (8.2%)	351,484 (8.3%)	0
	50-59	1,045,696 (18.4%)	260,604 (18.4%)	785,092 (18.4%)	0
	60-69	1,604,177 (28.3%)	400,653 (28.2%)	1,203,524 (28.3%)	0
	70-79	1,437,081 (25.3%)	359,683 (25.3%)	1,077,398 (25.3%)	0
	80-89	786,528 (13.9%)	197,074 (13.9%)	589,454 (13.8%)	0
	90+	96,685 (1.7%)	24,998 (1.8%)	71,687 (1.7%)	0.01
Neighbourhood income quintile	1 - Low	1,012,719 (17.8%)	259,849 (18.3%)	752,870 (17.7%)	0.02
	2	1,133,326 (20.0%)	285,773 (20.1%)	847,553 (19.9%)	0.01
	3	1,133,021 (20.0%)	282,123 (19.9%)	850,898 (20.0%)	0
	4	1,180,388 (20.8%)	291,594 (20.5%)	888,794 (20.9%)	0.01
	5 - High	1,201,075 (21.2%)	295,892 (20.8%)	905,183 (21.3%)	0.01
	Missing	16,775 (0.3%)	4,095 (0.3%)	12,680 (0.3%)	
Low-income senior (age 66+)	Yes	468,941 (15.6%)	111,694 (14.9%)	357,247 (15.9%)	0.03
Residence location	Urban	5,051,855 (89.0%)	1,256,231 (88.5%)	3,795,624 (89.1%)	0.02
	Rural	620,875 (10.9%)	161,995 (11.4%)	458,880 (10.8%)	0.02
	Missing	4,574 (0.1%)	1,100 (0.1%)	3,474 (0.1%)	
Recently living in Long-Term Care	Yes	115,231 (2.0%)	20,896 (1.5%)	94,335 (2.2%)	0.06
Landed immigrant (cat.)	Non-immigrant	5,024,782 (88.5%)	1,273,254 (89.7%)	3,751,528 (88.1%)	0.05
	Recent immigrant (10 years or less)	164,498 (2.9%)	37,357 (2.6%)	127,141 (3.0%)	0.02
	Past immigrant (more than 10 years)	488,024 (8.6%)	108,715 (7.7%)	379,309 (8.9%)	0.05
Ethnicity	General Population	5,302,511 (93.4%)	1,344,594 (94.7%)	3,957,917 (93.0%)	0.07
	Chinese	224,715 (4.0%)	49,099 (3.5%)	175,616 (4.1%)	0.03

© 2022 Giannakeas V et al. *JAMA Network Open*.

Description	Value	Total	Case	Control	Standardized Difference
	South Asian	150,078 (2.6%)	25,633 (1.8%)	124,445 (2.9%)	0.07
Time eligible in OHIP (years)	Mean (SD)<br>Median (IQR)	20.6 (5.3)<br>21.5 (19.1-24.0)	20.5 (5.3)<br>21.5 (19.1-24.0)	20.6 (5.3)<br>21.5 (19.1-24.0)	0
Health Services Utilization					
Any OHIP billings in the year prior	Yes	5,598,849 (98.6%)	1,399,563 (98.6%)	4,199,286 (98.6%)	0
Any ODB records on/in the year prior (age 66+)	Yes	2,917,066 (97.3%)	728,501 (97.2%)	2,188,565 (97.3%)	0.01
Physician Billings (2 years prior)					
Any core primary care visits to GP/FP	Yes	5,510,087 (97.1%)	1,378,052 (97.1%)	4,132,035 (97.0%)	0
Core primary care visits to GP/FP (cont.)	Mean (SD)<br>Median (IQR)	3.0 (3.7)<br>2 (1-4)	2.9 (3.6)<br>1 (1-3)	3.0 (3.8)<br>2 (1-4)	0.02
Core primary care visits to GP/FP (cat.)	0	167,217 (2.9%)	41,274 (2.9%)	125,943 (3.0%)	0
	1 - 2	3,198,252 (56.3%)	811,039 (57.1%)	2,387,213 (56.1%)	0.02
	3 - 4	1,201,970 (21.2%)	299,530 (21.1%)	902,440 (21.2%)	0
	5 - 9	846,965 (14.9%)	205,911 (14.5%)	641,054 (15.1%)	0.02
	10+	262,900 (4.6%)	61,572 (4.3%)	201,328 (4.7%)	0.02
Specialist visits (2 years prior)					
Dermatologist	Yes	877,189 (15.5%)	224,227 (15.8%)	652,962 (15.3%)	0.01
General surgeon	Yes	1,143,210 (20.1%)	298,490 (21.0%)	844,720 (19.8%)	0.03
Orthopedic surgeon	Yes	797,962 (14.1%)	194,499 (13.7%)	603,463 (14.2%)	0.01
Geriatrician	Yes	106,833 (1.9%)	23,349 (1.6%)	83,484 (2.0%)	0.02
Internal medicine specialist	Yes	3,366,565 (59.3%)	836,840 (59.0%)	2,529,725 (59.4%)	0.01
Endocrinologist	Yes	190,580 (3.4%)	49,507 (3.5%)	141,073 (3.3%)	0.01
Nephrologist	Yes	155,201 (2.7%)	39,497 (2.8%)	115,704 (2.7%)	0
Neurologist	Yes	504,083 (8.9%)	119,942 (8.5%)	384,141 (9.0%)	0.02
Psychiatrist	Yes	317,703 (5.6%)	73,949 (5.2%)	243,754 (5.7%)	0.02
OBGYN	Yes	498,801 (8.8%)	125,025 (8.8%)	373,776 (8.8%)	0
Geneticist	Yes	5,598 (0.1%)	1,409 (0.1%)	4,189 (0.1%)	0
ENT	Yes	688,708 (12.1%)	174,743 (12.3%)	513,965 (12.1%)	0.01
Urologist	Yes	675,709 (11.9%)	186,833 (13.2%)	488,876 (11.5%)	0.05
Gastroenterologist	Yes	546,089 (9.6%)	137,358 (9.7%)	408,731 (9.6%)	0
Oncologist	Yes	14,865 (0.3%)	5,388 (0.4%)	9,477 (0.2%)	0.03
Respirologist	Yes	378,174 (6.7%)	102,015 (7.2%)	276,159 (6.5%)	0.03
Rheumatologist	Yes	278,374 (4.9%)	64,510 (4.5%)	213,864 (5.0%)	0.02
Cardiologist	Yes	1,790,248 (31.5%)	438,440 (30.9%)	1,351,808 (31.7%)	0.02
Hematologist	Yes	160,143 (2.8%)	52,001 (3.7%)	108,142 (2.5%)	0.06
Emergency and inpatient admissions (2 years prior)					
Any unscheduled ED visits	Yes	2,374,409 (41.8%)	601,600 (42.4%)	1,772,809 (41.6%)	0.02
Unscheduled ED visits (cont.)	Mean (SD)	1.0 (2.0)	1.0 (2.0)	1.0 (2.1)	0.01

© 2022 Giannakeas V et al. *JAMA Network Open*.

Description	Value	Total	Case	Control	Standardized Difference
	Median (IQR)	0 (0-1)	0 (0-1)	0 (0-1)	
Unscheduled ED visits (cat.)	0	3,302,895 (58.2%)	817,726 (57.6%)	2,485,169 (58.4%)	0.02
	1	1,175,254 (20.7%)	296,130 (20.9%)	879,124 (20.6%)	0.01
	2	534,449 (9.4%)	135,339 (9.5%)	399,110 (9.4%)	0.01
	3+	664,706 (11.7%)	170,131 (12.0%)	494,575 (11.6%)	0.01
Any inpatient hospitalization episodes	Yes	663,502 (11.7%)	169,981 (12.0%)	493,521 (11.6%)	0.01
Inpatient hospitalization episodes (cont.)	Mean (SD)	0.2 (0.6)	0.2 (0.6)	0.2 (0.6)	0.01
	Median (IQR)	0 (0-0)	0 (0-0)	0 (0-0)	
Inpatient hospitalization episodes (cat.)	0	5,013,802 (88.3%)	1,249,345 (88.0%)	3,764,457 (88.4%)	0.01
	1	485,938 (8.6%)	123,488 (8.7%)	362,450 (8.5%)	0.01
	2	115,033 (2.0%)	30,075 (2.1%)	84,958 (2.0%)	0.01
	3+	62,531 (1.1%)	16,418 (1.2%)	46,113 (1.1%)	0.01
Primary care provider					
Rostered to family physician	Yes	4,868,959 (85.8%)	1,214,998 (85.6%)	3,653,961 (85.8%)	0.01
CBC test ordered by rostered family physician	Yes	3,925,398 (69.1%)	974,826 (68.7%)	2,950,572 (69.3%)	0.01
Up to date on cancer screening					
Pap smear (females age 18 to 70)	Yes	1,134,728 (64.2%)	276,006 (62.5%)	858,722 (64.7%)	0.05
Mammogram (females age 50 to 70)	Yes	868,591 (68.3%)	209,221 (65.9%)	659,370 (69.1%)	0.07
FOBT/sigmoidoscopy/colonoscopy (age 50 to 70)	Yes	1,721,945 (65.0%)	415,145 (62.8%)	1,306,800 (65.7%)	0.06
Comorbidities and chronic conditions					
Comorbidities					
Aggregate diagnosis groups (cont.)	Mean (SD)	7.1 (3.7)	7.1 (3.7)	7.1 (3.7)	0
	Median (IQR)	7 (4-10)	7 (4-10)	7 (4-10)	
Resource utilization band	0 - No or invalid diagnosis	33,804 (0.6%)	8,451 (0.6%)	25,353 (0.6%)	0
	1 - Healthy user	70,952 (1.2%)	17,738 (1.2%)	53,214 (1.2%)	0
	2 - Low user	415,228 (7.3%)	103,807 (7.3%)	311,421 (7.3%)	0
	3 - Moderate user	3,050,604 (53.7%)	762,651 (53.7%)	2,287,953 (53.7%)	0
	4 - High user	1,296,080 (22.8%)	324,020 (22.8%)	972,060 (22.8%)	0
	5 - Very high user	810,636 (14.3%)	202,659 (14.3%)	607,977 (14.3%)	0
Charlson score (cont.)	Mean (SD)	0.3 (0.9)	0.3 (0.9)	0.3 (0.8)	0.02
	Median (IQR)	0 (0-0)	0 (0-0)	0 (0-0)	
Charlson comorbidities (cat.)	0	4,882,890 (86.0%)	1,214,684 (85.6%)	3,668,206 (86.1%)	0.02
	1	385,248 (6.8%)	97,912 (6.9%)	287,336 (6.7%)	0.01
	2	187,718 (3.3%)	48,646 (3.4%)	139,072 (3.3%)	0.01
	3+	221,448 (3.9%)	58,084 (4.1%)	163,364 (3.8%)	0.01
Chronic conditions					
Asthma	Yes	537,653 (9.5%)	135,029 (9.5%)	402,624 (9.5%)	0

© 2022 Giannakeas V et al. *JAMA Network Open*.

Description	Value	Total	Case	Control	Standardized Difference
Congestive heart failure	Yes	393,049 (6.9%)	101,252 (7.1%)	291,797 (6.9%)	0.01
Inflammatory bowel disease	Yes	30,431 (0.5%)	7,410 (0.5%)	23,021 (0.5%)	0
Chronic obstructive pulmonary disease	Yes	386,047 (6.8%)	119,986 (8.5%)	266,061 (6.2%)	0.08
HIV	Yes	6,691 (0.1%)	1,885 (0.1%)	4,806 (0.1%)	0.01
Hypertension	Yes	3,306,541 (58.2%)	830,089 (58.5%)	2,476,452 (58.2%)	0.01
Dementia	Yes	223,044 (3.9%)	43,311 (3.1%)	179,733 (4.2%)	0.06
Diabetes	Yes	1,238,703 (21.8%)	303,166 (21.4%)	935,537 (22.0%)	0.01
Chronic rheumatoid arthritis	Yes	176,681 (3.1%)	41,026 (2.9%)	135,655 (3.2%)	0.02
Osteoarthritis	Yes	1,210,650 (21.3%)	292,529 (20.6%)	918,121 (21.6%)	0.02
Mood disorder	Yes	798,619 (14.1%)	191,338 (13.5%)	607,281 (14.3%)	0.02
Other mental health disorder	Yes	305,798 (5.4%)	77,442 (5.5%)	228,356 (5.4%)	0
Osteoporosis	Yes	103,466 (1.8%)	23,060 (1.6%)	80,406 (1.9%)	0.02
Renal disease	Yes	250,967 (4.4%)	63,041 (4.4%)	187,926 (4.4%)	0
Stroke	Yes	109,143 (1.9%)	25,536 (1.8%)	83,607 (2.0%)	0.01
Chronic coronary syndrome	Yes	660,914 (11.6%)	159,315 (11.2%)	501,599 (11.8%)	0.02
Acute myocardial infarction	Yes	139,154 (2.5%)	33,439 (2.4%)	105,715 (2.5%)	0.01
Medication use (age 66+)					
Concurrent medication use					
Number of concurrent medications being used (cont.)	Mean (SD)	4.4 (3.2)	4.4 (3.2)	4.4 (3.2)	0.01
	Median (IQR)	4 (2-6)	4 (2-6)	4 (2-6)	
Number of concurrent medications being used (cat.)	0	287,337 (9.6%)	72,453 (9.7%)	214,884 (9.6%)	0
	1 - 4	1,418,824 (47.3%)	355,321 (47.4%)	1,063,503 (47.3%)	0
	5 - 9	1,080,885 (36.0%)	269,166 (35.9%)	811,719 (36.1%)	0
	10+	212,374 (7.1%)	52,915 (7.1%)	159,459 (7.1%)	0
Recent medication use					
Antiplatelet					
Nonsteroidal anti-inflammatory (ASA-based)	Yes	113,066 (3.8%)	26,908 (3.6%)	86,158 (3.8%)	0.01
Nonsteroidal anti-inflammatory (non-ASA-based)	Yes	619,744 (20.7%)	151,206 (20.2%)	468,538 (20.8%)	0.02
Adenosine disphosphonate inhibitor	Yes	218,919 (7.3%)	54,120 (7.2%)	164,799 (7.3%)	0
Cardiovascular					
Coronary vasodilator (nitrate)	Yes	264,859 (8.8%)	64,685 (8.6%)	200,174 (8.9%)	0.01
Beta blocker	Yes	903,836 (30.1%)	223,369 (29.8%)	680,467 (30.2%)	0.01
Calcium channel blocker	Yes	937,762 (31.3%)	236,718 (31.6%)	701,044 (31.2%)	0.01
ACE inhibitor	Yes	987,543 (32.9%)	246,558 (32.9%)	740,985 (32.9%)	0
Angiotensin receptor agonist	Yes	754,041 (25.1%)	184,836 (24.6%)	569,205 (25.3%)	0.02
Lipid-lowering					

© 2022 Giannakeas V et al. *JAMA Network Open*.

Description	Value	Total	Case	Control	Standardized Difference
Statin	Yes	1,705,822 (56.9%)	414,870 (55.3%)	1,290,952 (57.4%)	0.04
Psychotropics					
Tricyclic antidepressant	Yes	151,967 (5.1%)	37,807 (5.0%)	114,160 (5.1%)	0
Selective serotonin reuptake inhibitor	Yes	351,210 (11.7%)	83,934 (11.2%)	267,276 (11.9%)	0.02
Platelet count (exposure definition)					
Continuous value for platelet count	Mean (SD)	239.1 (73.2)	245.7 (89.1)	237.0 (66.9)	0.11
	Median (IQR)	231 (193-274)	234 (194-282)	230 (193-272)	
Categorical platelet count (definition 1)	1 - Very low (≤10th percentile)	636,092 (11.2%)	170,382 (12.0%)	465,710 (10.9%)	0.03
	2 - Low (>10 to 25th percentile)	878,655 (15.5%)	203,017 (14.3%)	675,638 (15.9%)	0.04
	3 - Medium (>25 to <75th percentile)	2,756,889 (48.6%)	649,305 (45.7%)	2,107,584 (49.5%)	0.08
	4 - High (75 to <90th percentile)	823,871 (14.5%)	209,023 (14.7%)	614,848 (14.4%)	0.01
	5 - Very high (≥90th percentile)	581,797 (10.2%)	187,599 (13.2%)	394,198 (9.3%)	0.13
Categorical platelet count (definition 2)	1 - Thrombocytopenia (<150)	354,236 (6.2%)	103,239 (7.3%)	250,997 (5.9%)	0.06
	2 - Low normal (150 to 33.3rd percentile)	1,346,199 (23.7%)	312,501 (22.0%)	1,033,698 (24.3%)	0.05
	3 - Medium normal (>33.4 to <66.6th percentile)	2,602,504 (45.8%)	614,613 (43.3%)	1,987,891 (46.7%)	0.07
	4 - High normal (66.6th percentile to 450)	1,308,241 (23.0%)	356,063 (25.1%)	952,178 (22.4%)	0.06
	5 - Thrombocytosis (>450)	66,124 (1.2%)	32,910 (2.3%)	33,214 (0.8%)	0.12
Observation period					
Years from index CBC to outcome (cont.)	Mean (SD)	2.4 (2.1)	2.4 (2.1)		
	Median (IQR)	1.8 (0.7-3.5)	1.8 (0.7-3.5)		
Years from index CBC to outcome (cat.)	0-6 months	273,042 (19.2%)	273,042 (19.2%)		
	6-12 months	177,460 (12.5%)	177,460 (12.5%)		
	12-18 months	170,915 (12.0%)	170,915 (12.0%)		
	18-24 months	152,076 (10.7%)	152,076 (10.7%)		
	2-3 years	220,742 (15.6%)	220,742 (15.6%)		
	3-5 years	242,367 (17.1%)	242,367 (17.1%)		
	5-10 years	182,724 (12.9%)	182,724 (12.9%)		
Incident cancer events (Ontario Cancer Registry)					
Any cancer diagnosis in the OCR	Yes	1,419,326 (25.0%)	1,419,326 (100.0%)		
Solid tumour	Yes	1,219,140 (21.5%)	1,219,140 (85.9%)		
Colon	Yes	143,349 (2.5%)	143,349 (10.1%)		
Lung	Yes	167,823 (3.0%)	167,823 (11.8%)		
Breast (female only)	Yes	181,706 (6.3%)	181,706 (25.2%)		
Ovary (female only)	Yes	21,203 (0.7%)	21,203 (2.9%)		
Cervical (female only)	Yes	7,923 (0.3%)	7,923 (1.1%)		
Endometrial (female only)	Yes	48,759 (1.7%)	48,759 (6.8%)		

© 2022 Giannakeas V et al. *JAMA Network Open*.

Description	Value	Total	Case	Control	Standardized Difference
Prostate (male only)	Yes	174,026 (6.2%)	174,026 (24.9%)		
Thyroid	Yes	58,931 (1.0%)	58,931 (4.2%)		
Pancreas	Yes	36,861 (0.6%)	36,861 (2.6%)		
Stomach	Yes	27,590 (0.5%)	27,590 (1.9%)		
Kidney	Yes	41,248 (0.7%)	41,248 (2.9%)		
Bladder	Yes	70,164 (1.2%)	70,164 (4.9%)		
Liver	Yes	24,809 (0.4%)	24,809 (1.7%)		
Esophagus	Yes	13,351 (0.2%)	13,351 (0.9%)		
Other GI	Yes	15,674 (0.3%)	15,674 (1.1%)		
Brain	Yes	15,363 (0.3%)	15,363 (1.1%)		
Melanoma	Yes	56,460 (1.0%)	56,460 (4.0%)		
Head and neck	Yes	36,705 (0.6%)	36,705 (2.6%)		
Other solid tumour	Yes	77,195 (1.4%)	77,195 (5.4%)		
Hematologic tumour	Yes	200,186 (3.5%)	200,186 (14.1%)		
Leukemia	Yes	15,699 (0.3%)	15,699 (1.1%)		
Lymphoma	Yes	101,343 (1.8%)	101,343 (7.1%)		
Multiple myeloma	Yes	26,882 (0.5%)	26,882 (1.9%)		
Other hematologic tumour	Yes	56,262 (1.0%)	56,262 (4.0%)		

© 2022 Giannakeas V et al. *JAMA Network Open*.

eTable 5. Odds ratio of cancer by platelet count category and time from diagnosis. Select cancer sites.

		Colon				Lung			
**Categorical Time**	**Value**	**N, % (Case)**	**N, % (Control)**	**OR (95% CI)**	**P-value**	**N, % (Case)**	**N, % (Control)**	**OR (95% CI)**	**P-value**
0-6 months	1 - Very low (≤10th percentile)	1,820 (5.83%)	10,092 (10.77%)	0.73 (0.69-0.77)	<.0001	2,148 (6.61%)	11,045 (11.34%)	0.79 (0.75-0.83)	<.0001
	2 - Low (>10 to 25th percentile)	2,688 (8.60%)	14,722 (15.71%)	0.74 (0.70-0.77)	<.0001	2,748 (8.46%)	15,224 (15.63%)	0.74 (0.70-0.77)	<.0001
	3 - Medium (>25 to <75th percentile)	11,393 (36.47%)	46,289 (49.39%)	1.00 [Reference]		11,595 (35.70%)	47,505 (48.76%)	1.00 [Reference]	
	4 - High (75 to <90th percentile)	5,719 (18.31%)	13,688 (14.60%)	1.71 (1.65-1.78)	<.0001	6,005 (18.49%)	14,299 (14.68%)	1.73 (1.67-1.80)	<.0001
	5 - Very high (≥90th percentile)	9,621 (30.80%)	8,932 (9.53%)	4.38 (4.22-4.54)	<.0001	9,979 (30.73%)	9,352 (9.60%)	4.37 (4.22-4.53)	<.0001
6-12 months	1 - Very low (≤10th percentile)	1,473 (8.32%)	5,754 (10.84%)	0.86 (0.81-0.91)	<.0001	1,869 (8.76%)	7,291 (11.39%)	0.86 (0.81-0.90)	<.0001
	2 - Low (>10 to 25th percentile)	2,134 (12.06%)	8,385 (15.80%)	0.86 (0.81-0.90)	<.0001	2,489 (11.67%)	10,007 (15.63%)	0.83 (0.79-0.87)	<.0001
	3 - Medium (>25 to <75th percentile)	7,787 (44.01%)	26,259 (49.47%)	1.00 [Reference]		9,447 (44.28%)	31,548 (49.29%)	1.00 [Reference]	
	4 - High (75 to <90th percentile)	3,219 (18.19%)	7,755 (14.61%)	1.40 (1.33-1.47)	<.0001	3,630 (17.01%)	9,171 (14.33%)	1.32 (1.26-1.38)	<.0001
	5 - Very high (≥90th percentile)	3,082 (17.42%)	4,932 (9.29%)	2.11 (2.01-2.23)	<.0001	3,902 (18.29%)	5,994 (9.36%)	2.18 (2.08-2.29)	<.0001
12-18 months	1 - Very low (≤10th percentile)	1,643 (9.64%)	5,487 (10.74%)	0.95 (0.90-1.01)	0.1268	1,922 (9.46%)	6,691 (10.98%)	0.93 (0.88-0.98)	0.0087
	2 - Low (>10 to 25th percentile)	2,357 (13.83%)	8,179 (16.00%)	0.92 (0.87-0.96)	0.001	2,562 (12.61%)	9,760 (16.01%)	0.85 (0.81-0.89)	<.0001
	3 - Medium (>25 to <75th percentile)	7,977 (46.82%)	25,404 (49.70%)	1.00 [Reference]		9,241 (45.49%)	29,890 (49.04%)	1.00 [Reference]	
	4 - High (75 to <90th percentile)	2,804 (16.46%)	7,439 (14.55%)	1.20 (1.14-1.26)	<.0001	3,327 (16.38%)	8,785 (14.41%)	1.22 (1.17-1.28)	<.0001
	5 - Very high (≥90th percentile)	2,256 (13.24%)	4,602 (9.00%)	1.56 (1.48-1.66)	<.0001	3,264 (16.07%)	5,822 (9.55%)	1.82 (1.73-1.91)	<.0001
18-24 months	1 - Very low (≤10th percentile)	1,546 (10.38%)	4,882 (10.93%)	0.99 (0.93-1.06)	0.7936	1,828 (9.87%)	6,246 (11.25%)	0.94 (0.89-1.00)	0.0351
	2 - Low (>10 to 25th percentile)	2,169 (14.57%)	7,192 (16.10%)	0.94 (0.89-1.00)	0.0401	2,387 (12.89%)	8,929 (16.08%)	0.86 (0.81-0.90)	<.0001
	3 - Medium (>25 to <75th percentile)	7,067 (47.46%)	22,125 (49.53%)	1.00 [Reference]		8,444 (45.61%)	27,132 (48.85%)	1.00 [Reference]	
	4 - High (75 to <90th percentile)	2,342 (15.73%)	6,463 (14.47%)	1.14 (1.08-1.20)	<.0001	3,080 (16.64%)	8,085 (14.56%)	1.23 (1.17-1.29)	<.0001

© 2022 Giannakeas V et al. *JAMA Network Open*.

		Colon				Lung			
Categorical Time	Value	N, % (Case)	N, % (Control)	OR (95% CI)	P-value	N, % (Case)	N, % (Control)	OR (95% CI)	P-value
	5 - Very high (≥90th percentile)	1,765 (11.85%)	4,005 (8.97%)	1.38 (1.29-1.47)	<.0001	2,774 (14.98%)	5,147 (9.27%)	1.74 (1.65-1.83)	<.0001
2-3 years	1 - Very low (≤10th percentile)	2,276 (10.50%)	6,963 (10.71%)	1.01 (0.96-1.07)	0.6282	2,516 (9.62%)	8,589 (10.95%)	0.94 (0.90-0.99)	0.0229
	2 - Low (>10 to 25th percentile)	3,357 (15.49%)	10,309 (15.86%)	1.01 (0.96-1.05)	0.7113	3,504 (13.40%)	12,509 (15.94%)	0.90 (0.86-0.94)	<.0001
	3 - Medium (>25 to <75th percentile)	10,482 (48.37%)	32,488 (49.97%)	1.00 [Reference]		12,147 (46.44%)	39,114 (49.84%)	1.00 [Reference]	
	4 - High (75 to <90th percentile)	3,345 (15.44%)	9,447 (14.53%)	1.10 (1.05-1.15)	<.0001	4,264 (16.30%)	11,172 (14.24%)	1.23 (1.18-1.28)	<.0001
	5 - Very high (≥90th percentile)	2,210 (10.20%)	5,803 (8.93%)	1.18 (1.12-1.25)	<.0001	3,727 (14.25%)	7,090 (9.03%)	1.70 (1.62-1.78)	<.0001
3-5 years	1 - Very low (≤10th percentile)	2,622 (11.13%)	7,716 (10.92%)	1.04 (0.99-1.09)	0.1338	2,648 (9.39%)	9,235 (10.92%)	0.91 (0.87-0.96)	0.0002
	2 - Low (>10 to 25th percentile)	3,672 (15.59%)	11,431 (16.18%)	0.98 (0.94-1.02)	0.3889	3,832 (13.59%)	13,687 (16.18%)	0.89 (0.86-0.93)	<.0001
	3 - Medium (>25 to <75th percentile)	11,521 (48.91%)	35,208 (49.82%)	1.00 [Reference]		13,184 (46.75%)	42,028 (49.67%)	1.00 [Reference]	
	4 - High (75 to <90th percentile)	3,467 (14.72%)	10,153 (14.37%)	1.04 (1.00-1.09)	0.0569	4,657 (16.51%)	12,077 (14.27%)	1.23 (1.19-1.28)	<.0001
	5 - Very high (≥90th percentile)	2,273 (9.65%)	6,157 (8.71%)	1.13 (1.07-1.19)	<.0001	3,881 (13.76%)	7,579 (8.96%)	1.64 (1.57-1.71)	<.0001
5-10 years	1 - Very low (≤10th percentile)	1,833 (10.62%)	5,456 (10.54%)	1.03 (0.97-1.09)	0.3722	1,925 (9.25%)	6,646 (10.64%)	0.92 (0.87-0.97)	0.0037
	2 - Low (>10 to 25th percentile)	2,725 (15.79%)	8,183 (15.80%)	1.02 (0.97-1.07)	0.4886	2,752 (13.22%)	10,169 (16.28%)	0.86 (0.82-0.90)	<.0001
	3 - Medium (>25 to <75th percentile)	8,494 (49.21%)	25,963 (50.14%)	1.00 [Reference]		9,767 (46.91%)	31,089 (49.77%)	1.00 [Reference]	
	4 - High (75 to <90th percentile)	2,551 (14.78%)	7,502 (14.49%)	1.04 (0.99-1.09)	0.1368	3,549 (17.04%)	8,913 (14.27%)	1.27 (1.21-1.33)	<.0001
	5 - Very high (≥90th percentile)	1,659 (9.61%)	4,682 (9.04%)	1.08 (1.02-1.15)	0.0101	2,829 (13.59%)	5,649 (9.04%)	1.60 (1.52-1.69)	<.0001

© 2022 Giannakeas V et al. *JAMA Network Open*.

		Ovary				Stomach			
**Categorical Time**	**Value**	**N, % (Case)**	**N, % (Control)**	**OR (95% CI)**	**P-value**	**N, % (Case)**	**N, % (Control)**	**OR (95% CI)**	**P-value**
0-6 months	1 - Very low (≤10th percentile)	243 (5.48%)	1,392 (10.46%)	0.72 (0.62-0.84)	<.0001	414 (7.37%)	1,898 (11.26%)	0.91 (0.81-1.02)	0.1017
	2 - Low (>10 to 25th percentile)	350 (7.89%)	2,077 (15.61%)	0.69 (0.61-0.78)	<.0001	503 (8.95%)	2,598 (15.41%)	0.79 (0.71-0.88)	<.0001
	3 - Medium (>25 to <75th percentile)	1,615 (36.42%)	6,666 (50.11%)	1.00 [Reference]		2,039 (36.29%)	8,332 (49.44%)	1.00 [Reference]	
	4 - High (75 to <90th percentile)	830 (18.72%)	1,896 (14.25%)	1.82 (1.65-2.01)	<.0001	1,058 (18.83%)	2,452 (14.55%)	1.79 (1.64-1.95)	<.0001
	5 - Very high (≥90th percentile)	1,396 (31.48%)	1,271 (9.55%)	4.62 (4.19-5.09)	<.0001	1,604 (28.55%)	1,574 (9.34%)	4.27 (3.91-4.66)	<.0001
6-12 months	1 - Very low (≤10th percentile)	233 (9.17%)	804 (10.54%)	0.88 (0.75-1.04)	0.1262	336 (9.47%)	1,155 (10.85%)	0.95 (0.83-1.09)	0.498
	2 - Low (>10 to 25th percentile)	344 (13.53%)	1,188 (15.58%)	0.88 (0.77-1.01)	0.0695	513 (14.45%)	1,723 (16.18%)	0.97 (0.87-1.09)	0.6389
	3 - Medium (>25 to <75th percentile)	1,242 (48.86%)	3,787 (49.66%)	1.00 [Reference]		1,619 (45.62%)	5,299 (49.77%)	1.00 [Reference]	
	4 - High (75 to <90th percentile)	435 (17.11%)	1,091 (14.31%)	1.21 (1.07-1.38)	0.0031	612 (17.24%)	1,529 (14.36%)	1.31 (1.18-1.46)	<.0001
	5 - Very high (≥90th percentile)	288 (11.33%)	756 (9.91%)	1.16 (1.00-1.35)	0.0497	469 (13.21%)	941 (8.84%)	1.64 (1.44-1.85)	<.0001
12-18 months	1 - Very low (≤10th percentile)	218 (8.93%)	758 (10.35%)	0.84 (0.72-0.99)	0.0416	384 (11.73%)	1,072 (10.92%)	1.14 (1.00-1.29)	0.0546
	2 - Low (>10 to 25th percentile)	338 (13.84%)	1,145 (15.63%)	0.86 (0.75-0.99)	0.0352	481 (14.70%)	1,636 (16.66%)	0.93 (0.83-1.05)	0.2399
	3 - Medium (>25 to <75th percentile)	1,228 (50.29%)	3,597 (49.10%)	1.00 [Reference]		1,531 (46.78%)	4,856 (49.46%)	1.00 [Reference]	
	4 - High (75 to <90th percentile)	380 (15.56%)	1,122 (15.32%)	0.99 (0.87-1.14)	0.9202	498 (15.22%)	1,353 (13.78%)	1.17 (1.04-1.31)	0.0099
	5 - Very high (≥90th percentile)	278 (11.38%)	704 (9.61%)	1.16 (0.99-1.35)	0.0623	379 (11.58%)	902 (9.19%)	1.34 (1.17-1.53)	<.0001
18-24 months	1 - Very low (≤10th percentile)	222 (10.11%)	654 (9.93%)	1.02 (0.87-1.21)	0.7721	313 (10.34%)	1,001 (11.02%)	0.95 (0.83-1.09)	0.47
	2 - Low (>10 to 25th percentile)	355 (16.17%)	998 (15.16%)	1.07 (0.93-1.23)	0.3159	431 (14.24%)	1,450 (15.97%)	0.90 (0.80-1.02)	0.1096
	3 - Medium (>25 to <75th percentile)	1,105 (50.34%)	3,333 (50.62%)	1.00 [Reference]		1,480 (48.89%)	4,507 (49.63%)	1.00 [Reference]	
	4 - High (75 to <90th percentile)	298 (13.58%)	941 (14.29%)	0.95 (0.82-1.11)	0.5358	474 (15.66%)	1,270 (13.99%)	1.14 (1.01-1.28)	0.039

© 2022 Giannakeas V et al. *JAMA Network Open*.

		Ovary				Stomach			
**Categorical Time**	**Value**	**N, % (Case)**	**N, % (Control)**	**OR (95% CI)**	**P-value**	**N, % (Case)**	**N, % (Control)**	**OR (95% CI)**	**P-value**
	5 - Very high (≥90th percentile)	215 (9.79%)	659 (10.01%)	0.98 (0.83-1.16)	0.8472	329 (10.87%)	853 (9.39%)	1.18 (1.02-1.35)	0.0248
2-3 years	1 - Very low (≤10th percentile)	327 (10.06%)	1,017 (10.42%)	0.96 (0.84-1.10)	0.5943	482 (11.47%)	1,345 (10.67%)	1.14 (1.01-1.28)	0.0292
	2 - Low (>10 to 25th percentile)	511 (15.71%)	1,518 (15.56%)	1.01 (0.90-1.13)	0.8836	656 (15.62%)	2,018 (16.01%)	1.03 (0.93-1.14)	0.5571
	3 - Medium (>25 to <75th percentile)	1,628 (50.06%)	4,879 (50.01%)	1.00 [Reference]		1,971 (46.92%)	6,262 (49.69%)	1.00 [Reference]	
	4 - High (75 to <90th percentile)	506 (15.56%)	1,398 (14.33%)	1.09 (0.97-1.22)	0.1662	629 (14.97%)	1,852 (14.69%)	1.08 (0.97-1.20)	0.1452
	5 - Very high (≥90th percentile)	280 (8.61%)	944 (9.68%)	0.89 (0.77-1.03)	0.1055	463 (11.02%)	1,126 (8.93%)	1.31 (1.16-1.47)	<.0001
3-5 years	1 - Very low (≤10th percentile)	374 (10.33%)	1,201 (11.06%)	0.91 (0.80-1.04)	0.1559	506 (11.10%)	1,531 (11.19%)	1.01 (0.91-1.13)	0.8204
	2 - Low (>10 to 25th percentile)	560 (15.47%)	1,839 (16.93%)	0.89 (0.80-0.99)	0.0379	730 (16.01%)	2,226 (16.27%)	1.01 (0.91-1.11)	0.9072
	3 - Medium (>25 to <75th percentile)	1,819 (50.23%)	5,321 (48.98%)	1.00 [Reference]		2,223 (48.75%)	6,818 (49.84%)	1.00 [Reference]	
	4 - High (75 to <90th percentile)	539 (14.89%)	1,505 (13.85%)	1.05 (0.94-1.17)	0.4019	618 (13.55%)	1,920 (14.04%)	0.99 (0.89-1.09)	0.817
	5 - Very high (≥90th percentile)	329 (9.09%)	997 (9.18%)	0.97 (0.84-1.11)	0.6412	483 (10.59%)	1,185 (8.66%)	1.25 (1.11-1.41)	0.0002
5-10 years	1 - Very low (≤10th percentile)	272 (10.01%)	898 (11.02%)	0.90 (0.77-1.04)	0.1524	393 (11.69%)	1,083 (10.74%)	1.12 (0.98-1.27)	0.0845
	2 - Low (>10 to 25th percentile)	423 (15.57%)	1,324 (16.24%)	0.95 (0.83-1.07)	0.3873	541 (16.09%)	1,636 (16.22%)	1.02 (0.91-1.14)	0.7301
	3 - Medium (>25 to <75th percentile)	1,372 (50.50%)	4,064 (49.86%)	1.00 [Reference]		1,630 (48.48%)	5,028 (49.85%)	1.00 [Reference]	
	4 - High (75 to <90th percentile)	385 (14.17%)	1,162 (14.26%)	0.98 (0.86-1.12)	0.8008	481 (14.31%)	1,449 (14.37%)	1.02 (0.91-1.15)	0.6883
	5 - Very high (≥90th percentile)	265 (9.75%)	703 (8.62%)	1.12 (0.96-1.31)	0.1539	317 (9.43%)	890 (8.82%)	1.10 (0.96-1.26)	0.189

© 2022 Giannakeas V et al. *JAMA Network Open*.

eTable 6. Odds ratio of any solid tumour diagnosis (excluding liver) by change in platelet count category and time from cancer diagnosis.

Time from cancer diagnosis	Change in Platelet Count Percentile Value	N, % (Case)	N, % (Control)	OR (95% CI)	P-Value
0-6 months	1 - Large decrease (≤10th percentile)	8,494 (9.4%)	28,643 (10.6%)	1.08 (1.05-1.11)	<.0001
	2 - Small decrease (>10 to 25th percentile)	10,990 (12.2%)	42,253 (15.6%)	0.94 (0.92-0.96)	<.0001
	3 - No significant change (>25 to <75th percentile)	36,283 (40.1%)	131,443 (48.5%)	1.00 [Reference]	
	4 - Small increase (75 to <90th percentile)	14,887 (16.5%)	41,343 (15.2%)	1.31 (1.28-1.34)	<.0001
	5 - Large increase (≥90th percentile)	19,750 (21.8%)	27,530 (10.2%)	2.62 (2.56-2.68)	<.0001
6-12 months	1 - Large decrease (≤10th percentile)	7,020 (10.3%)	21,563 (10.6%)	1.05 (1.02-1.09)	0.0008
	2 - Small decrease (>10 to 25th percentile)	9,790 (14.4%)	31,848 (15.6%)	0.99 (0.97-1.02)	0.5287
	3 - No significant change (>25 to <75th percentile)	30,695 (45.2%)	99,042 (48.7%)	1.00 [Reference]	
	4 - Small increase (75 to <90th percentile)	11,268 (16.6%)	31,008 (15.2%)	1.18 (1.15-1.21)	<.0001
	5 - Large increase (≥90th percentile)	9,081 (13.4%)	20,101 (9.9%)	1.46 (1.42-1.51)	<.0001
1-2 years	1 - Large decrease (≤10th percentile)	10,404 (10.3%)	30,958 (10.3%)	1.03 (1.01-1.06)	0.0113
	2 - Small decrease (>10 to 25th percentile)	15,050 (15.0%)	47,148 (15.6%)	0.98 (0.96-1.00)	0.0535
	3 - No significant change (>25 to <75th percentile)	48,403 (48.1%)	148,583 (49.2%)	1.00 [Reference]	
	4 - Small increase (75 to <90th percentile)	15,763 (15.7%)	46,051 (15.3%)	1.05 (1.03-1.07)	<.0001
	5 - Large increase (≥90th percentile)	11,024 (11.0%)	29,192 (9.7%)	1.16 (1.13-1.19)	<.0001
2-5 years	1 - Large decrease (≤10th percentile)	12,076 (10.1%)	36,007 (10.0%)	1.02 (1.00-1.04)	0.1094
	2 - Small decrease (>10 to 25th percentile)	18,452 (15.4%)	55,580 (15.4%)	1.01 (0.99-1.03)	0.4105
	3 - No significant change (>25 to <75th percentile)	58,748 (48.9%)	178,385 (49.5%)	1.00 [Reference]	

© 2022 Giannakeas V et al. *JAMA Network Open*.

Time from cancer diagnosis	Change in Platelet Count Percentile Value	N, % (Case)	N, % (Control)	OR (95% CI)	P-Value
	4 - Small increase (75 to <90th percentile)	18,538 (15.4%)	55,595 (15.4%)	1.01 (0.99-1.03)	0.1875
	5 - Large increase (≥90th percentile)	12,229 (10.2%)	34,562 (9.6%)	1.08 (1.05-1.10)	<.0001
5-10 years	1 - Large decrease (≤10th percentile)	6,539 (11.5%)	19,425 (11.4%)	1.01 (0.98-1.04)	0.4869
	2 - Small decrease (>10 to 25th percentile)	10,002 (17.6%)	30,704 (18.1%)	0.98 (0.95-1.00)	0.102
	3 - No significant change (>25 to <75th percentile)	28,116 (49.6%)	84,446 (49.7%)	1.00 [Reference]	
	4 - Small increase (75 to <90th percentile)	7,399 (13.1%)	22,136 (13.0%)	1.00 (0.97-1.03)	0.78
	5 - Large increase (≥90th percentile)	4,615 (8.1%)	13,302 (7.8%)	1.04 (1.01-1.08)	0.0239

© 2022 Giannakeas V et al. *JAMA Network Open*.

eTable 7. Odds ratio of cancer by change in platelet count category and time from diagnosis. Select cancer sites.

		Colon				Lung			
Categorical Time	Value	N, % (Case)	N, % (Control)	OR (95% CI)	P-value	N, % (Case)	N, % (Control)	OR (95% CI)	P-value
0-6 months	1 - Large decrease (≤10th pctl)	794 (6.59%)	3,660 (10.13%)	1.02 (0.93-1.11)	0.7074	1,335 (9.72%)	4,591 (11.14%)	1.33 (1.24-1.43)	<.0001
	2 - Small decrease (>10 to 25th pctl)	999 (8.29%)	5,645 (15.62%)	0.84 (0.77-0.90)	<.0001	1,332 (9.70%)	6,348 (15.40%)	0.96 (0.89-1.02)	0.1953
	3 - No significant change (>25 to <75th pctl)	3,792 (31.48%)	17,663 (48.88%)	1.00 [Reference]		4,330 (31.52%)	19,677 (47.74%)	1.00 [Reference]	
	4 - Small increase (75 to <90th pctl)	2,169 (18.01%)	5,499 (15.22%)	1.87 (1.76-1.99)	<.0001	2,314 (16.84%)	6,299 (15.28%)	1.70 (1.61-1.81)	<.0001
	5 - Large increase (≥90th pctl)	4,292 (35.63%)	3,671 (10.16%)	5.52 (5.21-5.86)	<.0001	4,427 (32.22%)	4,299 (10.43%)	4.77 (4.51-5.04)	<.0001
6-12 months	1 - Large decrease (≤10th pctl)	738 (8.99%)	2,572 (10.45%)	1.05 (0.96-1.15)	0.3158	1,196 (11.34%)	3,336 (10.54%)	1.29 (1.20-1.39)	<.0001
	2 - Small decrease (>10 to 25th pctl)	906 (11.04%)	3,839 (15.59%)	0.86 (0.79-0.93)	0.0003	1,420 (13.46%)	4,930 (15.58%)	1.03 (0.96-1.10)	0.4622
	3 - No significant change (>25 to <75th pctl)	3,317 (40.42%)	12,045 (48.92%)	1.00 [Reference]		4,313 (40.89%)	15,368 (48.57%)	1.00 [Reference]	
	4 - Small increase (75 to <90th pctl)	1,584 (19.30%)	3,764 (15.29%)	1.54 (1.43-1.65)	<.0001	1,783 (16.91%)	4,831 (15.27%)	1.32 (1.24-1.41)	<.0001
	5 - Large increase (≥90th pctl)	1,662 (20.25%)	2,401 (9.75%)	2.55 (2.37-2.75)	<.0001	1,835 (17.40%)	3,176 (10.04%)	2.07 (1.93-2.21)	<.0001
12-18 months	1 - Large decrease (≤10th pctl)	793 (9.83%)	2,506 (10.35%)	1.04 (0.95-1.14)	0.3568	1,301 (12.91%)	3,323 (10.99%)	1.37 (1.27-1.47)	<.0001
	2 - Small decrease (>10 to 25th pctl)	1,114 (13.81%)	3,774 (15.59%)	0.97 (0.90-1.05)	0.4754	1,523 (15.11%)	4,730 (15.64%)	1.11 (1.04-1.19)	0.0016
	3 - No significant change (>25 to <75th pctl)	3,630 (44.99%)	11,959 (49.40%)	1.00 [Reference]		4,242 (42.09%)	14,665 (48.50%)	1.00 [Reference]	
	4 - Small increase (75 to <90th pctl)	1,413 (17.51%)	3,729 (15.40%)	1.25 (1.16-1.34)	<.0001	1,562 (15.50%)	4,535 (15.00%)	1.19 (1.12-1.28)	<.0001
	5 - Large increase (≥90th pctl)	1,119 (13.87%)	2,239 (9.25%)	1.65 (1.52-1.79)	<.0001	1,451 (14.40%)	2,984 (9.87%)	1.70 (1.58-1.82)	<.0001
18-24 months	1 - Large decrease (≤10th pctl)	693 (10.28%)	2,029 (10.03%)	1.11 (1.01-1.22)	0.0289	1,153 (12.88%)	2,877 (10.71%)	1.34 (1.24-1.44)	<.0001
	2 - Small decrease (>10 to 25th pctl)	955 (14.16%)	3,180 (15.72%)	0.97 (0.90-1.06)	0.5415	1,301 (14.53%)	4,194 (15.62%)	1.03 (0.96-1.10)	0.4549
	3 - No significant change (>25 to <75th pctl)	3,115 (46.20%)	10,125 (50.06%)	1.00 [Reference]		3,944 (44.06%)	13,051 (48.60%)	1.00 [Reference]	
	4 - Small increase (75 to <90th pctl)	1,169 (17.34%)	3,038 (15.02%)	1.25 (1.16-1.35)	<.0001	1,390 (15.53%)	4,059 (15.11%)	1.14 (1.06-1.22)	0.0004

© 2022 Giannakeas V et al. *JAMA Network Open*.

		Colon				Lung			
**Categorical Time**	**Value**	**N, % (Case)**	**N, % (Control)**	**OR (95% CI)**	**P-value**	**N, % (Case)**	**N, % (Control)**	**OR (95% CI)**	**P-value**
	5 - Large increase (≥90th pctl)	810 (12.01%)	1,854 (9.17%)	1.42 (1.30-1.56)	<.0001	1,164 (13.00%)	2,675 (9.96%)	1.45 (1.34-1.57)	<.0001
2-3 years	1 - Large decrease (≤10th pctl)	904 (9.56%)	2,792 (9.84%)	0.98 (0.90-1.06)	0.6224	1,550 (12.70%)	3,799 (10.37%)	1.35 (1.26-1.44)	<.0001
	2 - Small decrease (>10 to 25th pctl)	1,343 (14.20%)	4,469 (15.75%)	0.91 (0.85-0.97)	0.0062	1,840 (15.07%)	5,810 (15.86%)	1.04 (0.98-1.11)	0.1718
	3 - No significant change (>25 to <75th pctl)	4,690 (49.58%)	14,197 (50.02%)	1.00 [Reference]		5,394 (44.18%)	17,723 (48.39%)	1.00 [Reference]	
	4 - Small increase (75 to <90th pctl)	1,542 (16.30%)	4,286 (15.10%)	1.09 (1.02-1.17)	0.0103	1,903 (15.59%)	5,655 (15.44%)	1.11 (1.04-1.18)	0.0009
	5 - Large increase (≥90th pctl)	981 (10.37%)	2,636 (9.29%)	1.13 (1.04-1.22)	0.0031	1,521 (12.46%)	3,637 (9.93%)	1.38 (1.29-1.48)	<.0001
3-5 years	1 - Large decrease (≤10th pctl)	969 (10.05%)	2,798 (9.68%)	1.06 (0.98-1.15)	0.1457	1,501 (12.19%)	3,797 (10.27%)	1.29 (1.21-1.38)	<.0001
	2 - Small decrease (>10 to 25th pctl)	1,501 (15.57%)	4,536 (15.69%)	1.01 (0.95-1.08)	0.6904	1,867 (15.16%)	5,685 (15.38%)	1.07 (1.01-1.13)	0.0311
	3 - No significant change (>25 to <75th pctl)	4,772 (49.51%)	14,617 (50.55%)	1.00 [Reference]		5,624 (45.66%)	18,288 (49.49%)	1.00 [Reference]	
	4 - Small increase (75 to <90th pctl)	1,457 (15.12%)	4,313 (14.92%)	1.03 (0.97-1.11)	0.3237	1,920 (15.59%)	5,664 (15.33%)	1.10 (1.04-1.17)	0.0013
	5 - Large increase (≥90th pctl)	939 (9.74%)	2,650 (9.17%)	1.09 (1.00-1.18)	0.0464	1,406 (11.41%)	3,520 (9.53%)	1.31 (1.22-1.40)	<.0001
5-10 years	1 - Large decrease (≤10th pctl)	695 (11.33%)	2,104 (11.44%)	0.99 (0.90-1.09)	0.8018	1,050 (13.03%)	2,851 (11.79%)	1.17 (1.08-1.27)	0.0001
	2 - Small decrease (>10 to 25th pctl)	1,031 (16.81%)	3,292 (17.89%)	0.94 (0.86-1.01)	0.1094	1,378 (17.09%)	4,355 (18.01%)	1.00 (0.93-1.08)	0.963
	3 - No significant change (>25 to <75th pctl)	3,118 (50.84%)	9,323 (50.67%)	1.00 [Reference]		3,766 (46.72%)	11,927 (49.32%)	1.00 [Reference]	
	4 - Small increase (75 to <90th pctl)	766 (12.49%)	2,315 (12.58%)	0.99 (0.90-1.08)	0.8309	1,101 (13.66%)	3,199 (13.23%)	1.09 (1.01-1.18)	0.0269
	5 - Large increase (≥90th pctl)	523 (8.53%)	1,365 (7.42%)	1.15 (1.03-1.28)	0.0142	766 (9.50%)	1,851 (7.65%)	1.32 (1.20-1.45)	<.0001

© 2022 Giannakeas V et al. *JAMA Network Open*.

		Ovary				Stomach			
**Categorical Time**	**Value**	**N, % (Case)**	**N, % (Control)**	**OR (95% CI)**	**P-value**	**N, % (Case)**	**N, % (Control)**	**OR (95% CI)**	**P-value**
0-6 months	1 - Large decrease (≤10th pctl)	97 (6.07%)	480 (10.01%)	1.01 (0.79-1.29)	0.9506	185 (7.80%)	738 (10.37%)	1.16 (0.96-1.39)	0.1218
	2 - Small decrease (>10 to 25th pctl)	136 (8.51%)	741 (15.46%)	0.90 (0.73-1.11)	0.3245	172 (7.25%)	1,136 (15.96%)	0.69 (0.58-0.83)	<.0001
	3 - No significant change (>25 to <75th pctl)	462 (28.91%)	2,359 (49.21%)	1.00 [Reference]		745 (31.41%)	3,437 (48.30%)	1.00 [Reference]	
	4 - Small increase (75 to <90th pctl)	248 (15.52%)	736 (15.35%)	1.71 (1.43-2.05)	<.0001	407 (17.16%)	1,068 (15.01%)	1.77 (1.54-2.04)	<.0001
	5 - Large increase (≥90th pctl)	655 (40.99%)	478 (9.97%)	7.23 (6.12-8.53)	<.0001	863 (36.38%)	737 (10.36%)	5.51 (4.82-6.29)	<.0001
6-12 months	1 - Large decrease (≤10th pctl)	119 (10.54%)	351 (10.36%)	1.08 (0.86-1.35)	0.5295	167 (9.56%)	585 (11.17%)	0.98 (0.81-1.18)	0.8258
	2 - Small decrease (>10 to 25th pctl)	130 (11.51%)	566 (16.71%)	0.72 (0.58-0.90)	0.0037	252 (14.43%)	806 (15.39%)	1.06 (0.91-1.25)	0.4461
	3 - No significant change (>25 to <75th pctl)	518 (45.88%)	1,653 (48.80%)	1.00 [Reference]		746 (42.73%)	2,557 (48.82%)	1.00 [Reference]	
	4 - Small increase (75 to <90th pctl)	210 (18.60%)	504 (14.88%)	1.33 (1.10-1.61)	0.0029	298 (17.07%)	769 (14.68%)	1.33 (1.14-1.56)	0.0003
	5 - Large increase (≥90th pctl)	152 (13.46%)	313 (9.24%)	1.55 (1.25-1.93)	<.0001	283 (16.21%)	521 (9.95%)	1.87 (1.58-2.21)	<.0001
12-18 months	1 - Large decrease (≤10th pctl)	87 (8.15%)	335 (10.47%)	0.80 (0.62-1.03)	0.0861	171 (10.48%)	505 (10.32%)	1.04 (0.86-1.26)	0.6826
	2 - Small decrease (>10 to 25th pctl)	142 (13.31%)	486 (15.18%)	0.90 (0.73-1.11)	0.3115	211 (12.94%)	730 (14.92%)	0.88 (0.74-1.05)	0.1689
	3 - No significant change (>25 to <75th pctl)	519 (48.64%)	1,591 (49.70%)	1.00 [Reference]		797 (48.87%)	2,444 (49.95%)	1.00 [Reference]	
	4 - Small increase (75 to <90th pctl)	191 (17.90%)	477 (14.90%)	1.22 (1.00-1.48)	0.0453	258 (15.82%)	727 (14.86%)	1.09 (0.92-1.28)	0.308
	5 - Large increase (≥90th pctl)	128 (12.00%)	312 (9.75%)	1.25 (1.00-1.57)	0.0533	194 (11.89%)	487 (9.95%)	1.22 (1.02-1.47)	0.0334
18-24 months	1 - Large decrease (≤10th pctl)	96 (10.01%)	278 (9.66%)	1.01 (0.79-1.31)	0.9115	174 (11.73%)	437 (9.82%)	1.29 (1.06-1.57)	0.0127
	2 - Small decrease (>10 to 25th pctl)	146 (15.22%)	433 (15.05%)	0.99 (0.80-1.23)	0.9258	198 (13.34%)	683 (15.34%)	0.93 (0.78-1.11)	0.4262
	3 - No significant change (>25 to <75th pctl)	486 (50.68%)	1,426 (49.57%)	1.00 [Reference]		684 (46.09%)	2,191 (49.21%)	1.00 [Reference]	
	4 - Small increase (75 to <90th pctl)	142 (14.81%)	425 (14.77%)	0.98 (0.79-1.22)	0.8563	255 (17.18%)	697 (15.66%)	1.18 (0.99-1.39)	0.0581
	5 - Large increase (≥90th pctl)	89 (9.28%)	315 (10.95%)	0.83 (0.64-1.07)	0.1469	173 (11.66%)	444 (9.97%)	1.26 (1.03-1.53)	0.0225

© 2022 Giannakeas V et al. *JAMA Network Open*.

		Ovary				Stomach			
Categorical Time	Value	N, % (Case)	N, % (Control)	OR (95% CI)	P-value	N, % (Case)	N, % (Control)	OR (95% CI)	P-value
2-3 years	1 - Large decrease (≤10th pctl)	137 (9.88%)	425 (10.21%)	0.93 (0.75-1.15)	0.4994	211 (10.65%)	586 (9.86%)	1.11 (0.93-1.33)	0.23
	2 - Small decrease (>10 to 25th pctl)	208 (15.00%)	639 (15.36%)	0.94 (0.79-1.12)	0.4929	312 (15.75%)	925 (15.56%)	1.04 (0.90-1.21)	0.5961
	3 - No significant change (>25 to <75th pctl)	712 (51.33%)	2,056 (49.41%)	1.00 [Reference]		961 (48.51%)	2,964 (49.87%)	1.00 [Reference]	
	4 - Small increase (75 to <90th pctl)	201 (14.49%)	627 (15.07%)	0.93 (0.77-1.11)	0.4019	311 (15.70%)	921 (15.50%)	1.04 (0.90-1.21)	0.5785
	5 - Large increase (≥90th pctl)	129 (9.30%)	414 (9.95%)	0.90 (0.72-1.12)	0.3319	186 (9.39%)	547 (9.20%)	1.05 (0.88-1.26)	0.5972
3-5 years	1 - Large decrease (≤10th pctl)	132 (9.07%)	420 (9.62%)	0.96 (0.78-1.19)	0.7121	174 (8.59%)	608 (10.00%)	0.86 (0.71-1.03)	0.1026
	2 - Small decrease (>10 to 25th pctl)	234 (16.08%)	685 (15.69%)	1.04 (0.88-1.24)	0.6237	342 (16.88%)	911 (14.99%)	1.13 (0.97-1.30)	0.1069
	3 - No significant change (>25 to <75th pctl)	721 (49.55%)	2,203 (50.47%)	1.00 [Reference]		1,011 (49.90%)	3,025 (49.77%)	1.00 [Reference]	
	4 - Small increase (75 to <90th pctl)	224 (15.40%)	654 (14.98%)	1.05 (0.88-1.24)	0.6099	290 (14.31%)	966 (15.89%)	0.90 (0.77-1.04)	0.1554
	5 - Large increase (≥90th pctl)	144 (9.90%)	403 (9.23%)	1.09 (0.89-1.35)	0.4079	209 (10.32%)	568 (9.35%)	1.10 (0.92-1.31)	0.2946
5-10 years	1 - Large decrease (≤10th pctl)	113 (11.58%)	334 (11.41%)	0.99 (0.78-1.25)	0.9316	151 (11.30%)	449 (11.20%)	1.04 (0.85-1.28)	0.7091
	2 - Small decrease (>10 to 25th pctl)	174 (17.83%)	533 (18.20%)	0.96 (0.78-1.17)	0.6571	241 (18.04%)	720 (17.96%)	1.03 (0.87-1.22)	0.7195
	3 - No significant change (>25 to <75th pctl)	489 (50.10%)	1,432 (48.91%)	1.00 [Reference]		658 (49.25%)	2,032 (50.70%)	1.00 [Reference]	
	4 - Small increase (75 to <90th pctl)	131 (13.42%)	393 (13.42%)	0.98 (0.78-1.22)	0.8315	174 (13.02%)	527 (13.15%)	1.02 (0.84-1.24)	0.8269
	5 - Large increase (≥90th pctl)	69 (7.07%)	236 (8.06%)	0.85 (0.64-1.14)	0.2854	112 (8.38%)	280 (6.99%)	1.24 (0.98-1.57)	0.0792

© 2022 Giannakeas V et al. *JAMA Network Open*.

eFigure 1. Study design criteria among matched individuals

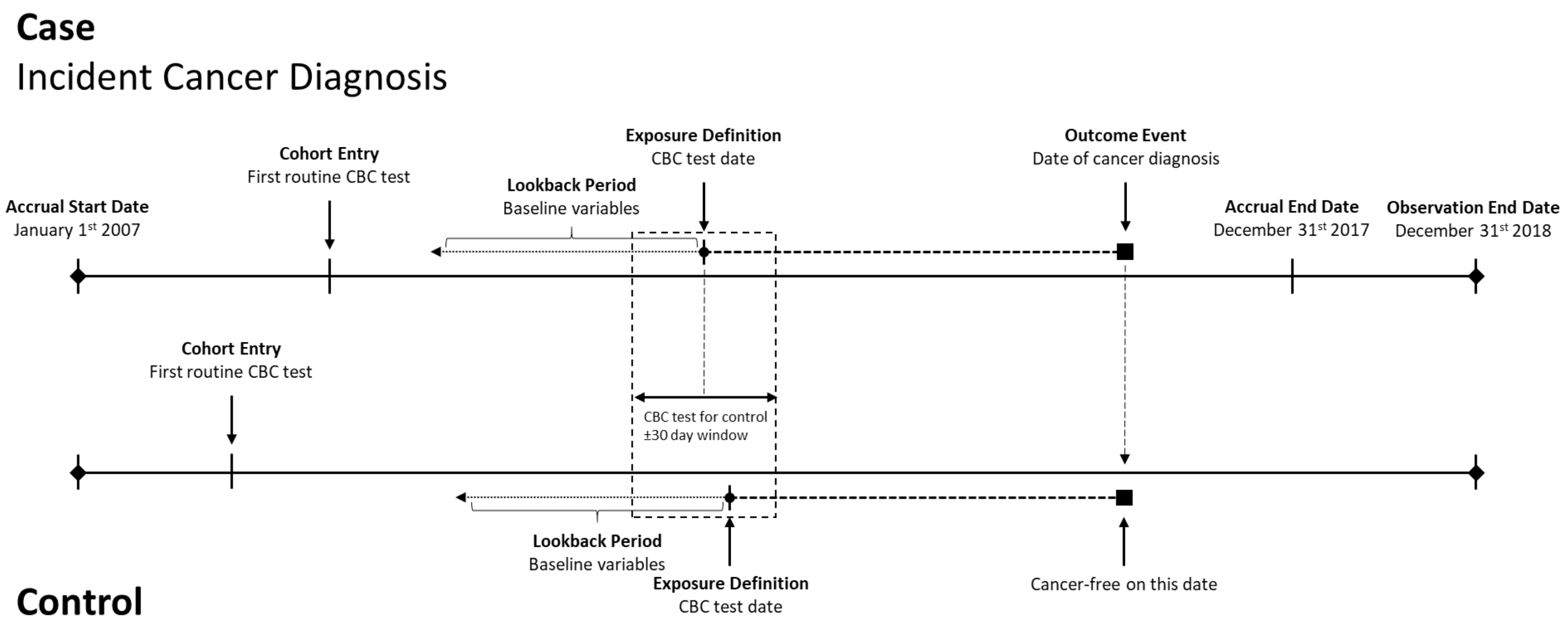

© 2022 Giannakeas V et al. *JAMA Network Open*.

eFigure 2. Age- and sex-specific platelet count reference distributions for exposure definition

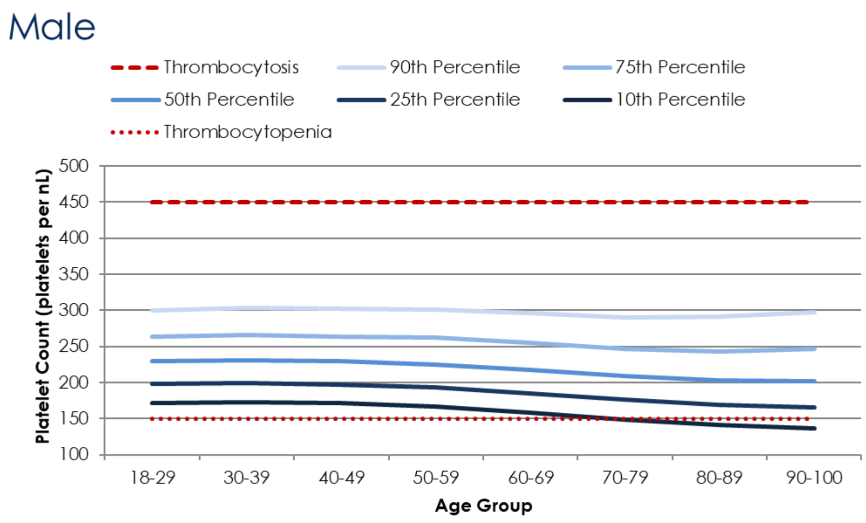

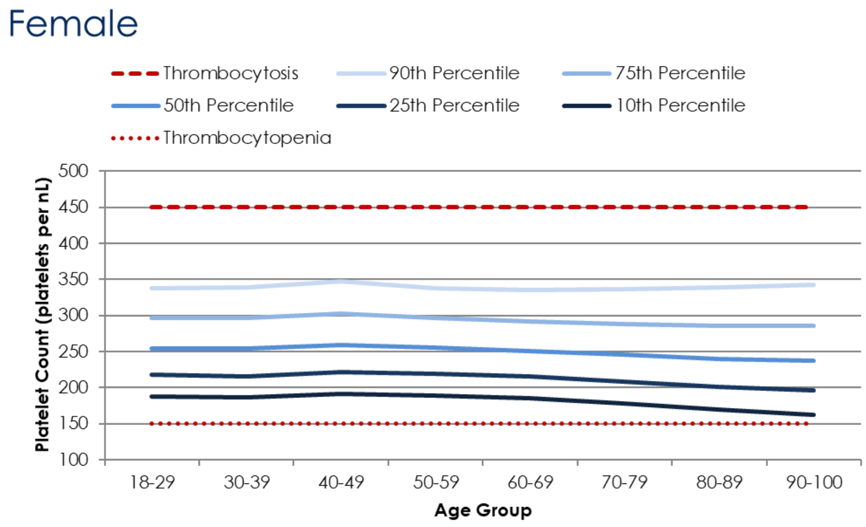

Male

		Platelet count observation percentile				
**Age**	**N**	**10th**	**25th**	**50th**	**75th**	**90th**
18-29	1,417,076	172	198	229	264	300
30-39	1,770,053	173	199	231	266	303
40-49	2,948,869	171	197	229	264	302
50-59	4,161,931	166	193	225	262	301
60-69	4,126,398	158	185	218	255	296
70-79	2,895,422	149	176	209	247	290
80-89	1,438,574	141	169	203	243	291
90-100	212,467	136	165	202	246	297

Female

		Platelet count observation percentile				
**Age**	**N**	**10th**	**25th**	**50th**	**75th**	**90th**
18-29	3,393,141	188	218	254	296	338
30-39	4,139,515	186	216	254	296	339
40-49	4,690,125	191	222	259	303	348
50-59	5,199,080	189	219	255	296	338
60-69	4,638,692	185	215	251	292	335
70-79	3,487,173	178	208	246	288	336
80-89	2,286,750	169	201	240	286	339
90-100	568,402	162	196	237	286	343

Note: Observations inversely weighted based on the number of CBC tests per-subject, for each sex-age category

© 2022 Giannakeas V et al. *JAMA Network Open*.

eFigure 3. Age- and sex-specific platelet count reference distributions for secondary exposure definition

Male

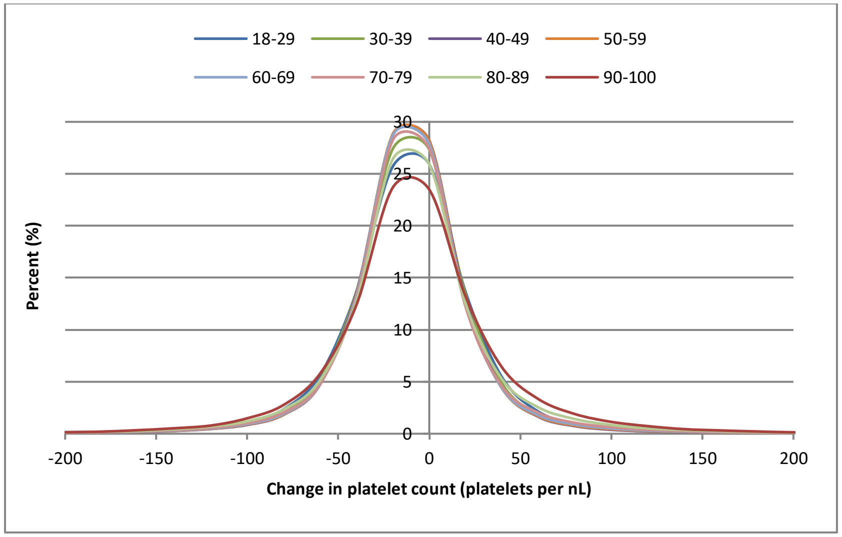

		Change in platelet count observation percentile				
Age	N	10th	25th	50th	75th	90th
18-29	239,713	-39	-19	-1	17	37
30-39	407,352	-36	-18	-1	16	34
40-49	870,005	-35	-17	-1	15	32
50-59	1,559,807	-35	-17	-1	15	33
60-69	1,950,142	-36	-17	-1	15	33
70-79	1,588,074	-36	-17	-1	15	35
80-89	835,988	-40	-18	0	18	42
90-100	125,702	-44	-19	1	22	52

Female

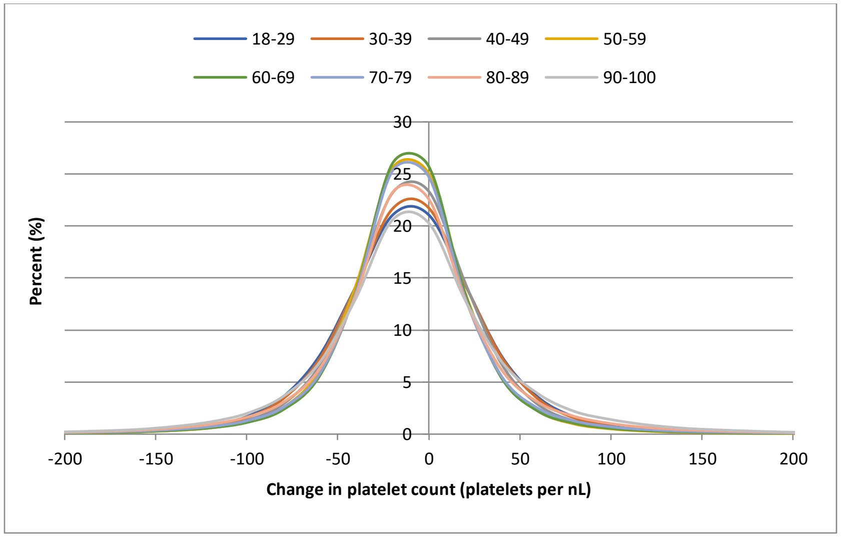

		Change in platelet count observation percentile				
Age	N	10th	25th	50th	75th	90th
18-29	762,668	-49	-24	0	24	48
30-39	1,267,171	-48	-23	0	23	47
40-49	1,664,504	-44	-21	0	21	43
50-59	2,144,471	-41	-20	-1	17	37
60-69	2,246,168	-38	-19	-1	17	37
70-79	1,907,064	-41	-19	-1	18	40
80-89	1,300,664	-47	-21	-1	21	47
90-100	334,804	-53	-24	0	25	58

Note: Observations inversely weighted based on the number of CBC tests per-subject, for each sex-age category

© 2022 Giannakeas V et al. *JAMA Network Open*.

eFigure 4. Odds ratio of cancer by platelet count category and time from cancer diagnosis. Additional cancer sites.

Breast (N = 65,703)

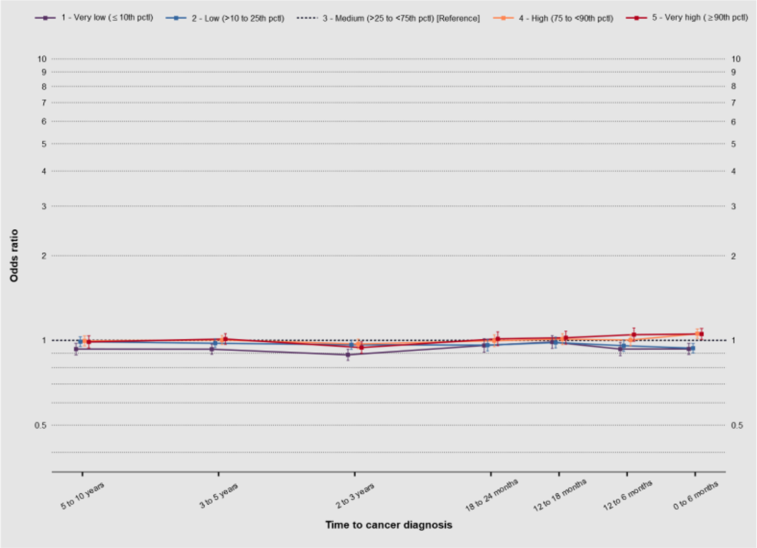

Prostate (N = 62,770)

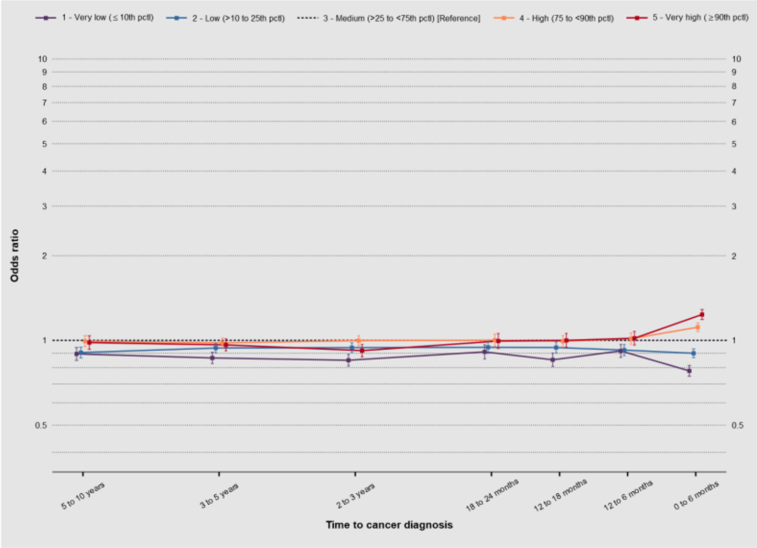

Melanoma (N = 20,167)

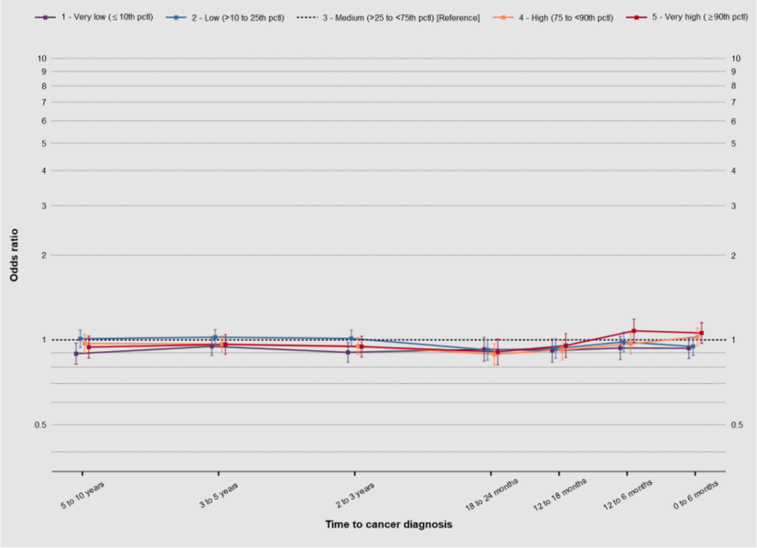

Thyroid (N = 21,559)

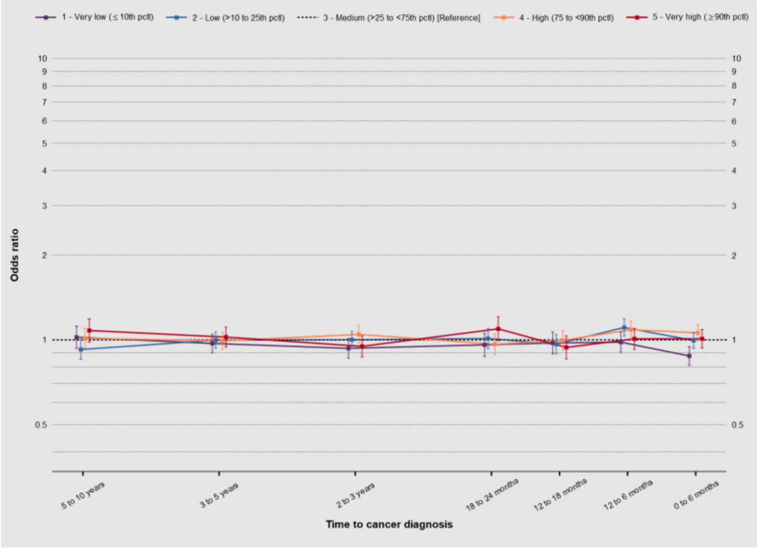

© 2022 Giannakeas V et al. *JAMA Network Open*.

Esophagus (N = 4,691)

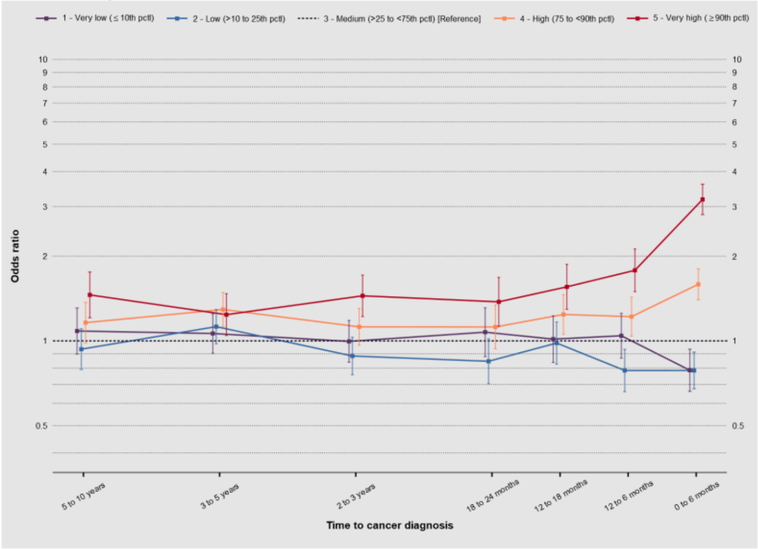

Kidney (N = 14,057)

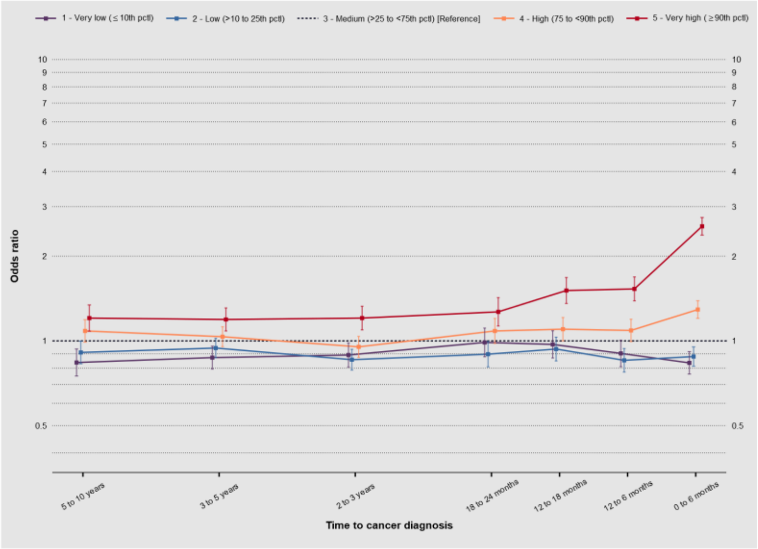

Pancreas (N = 12,009)

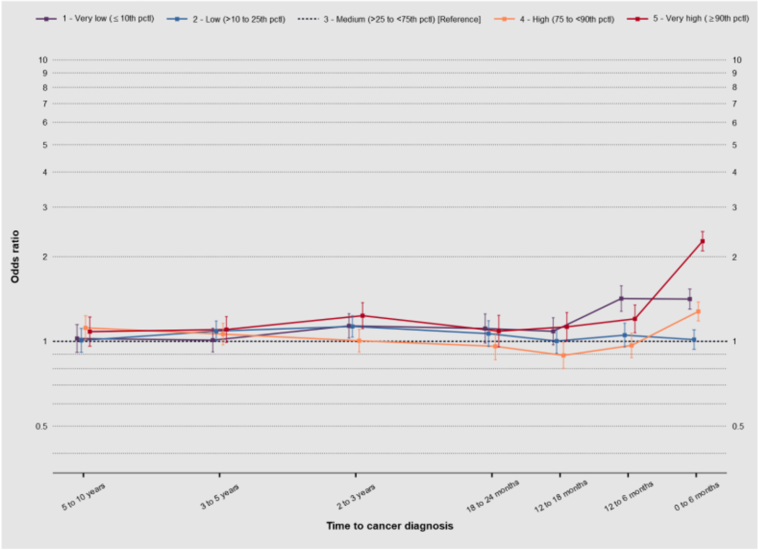

Other GI (N = 5,259)

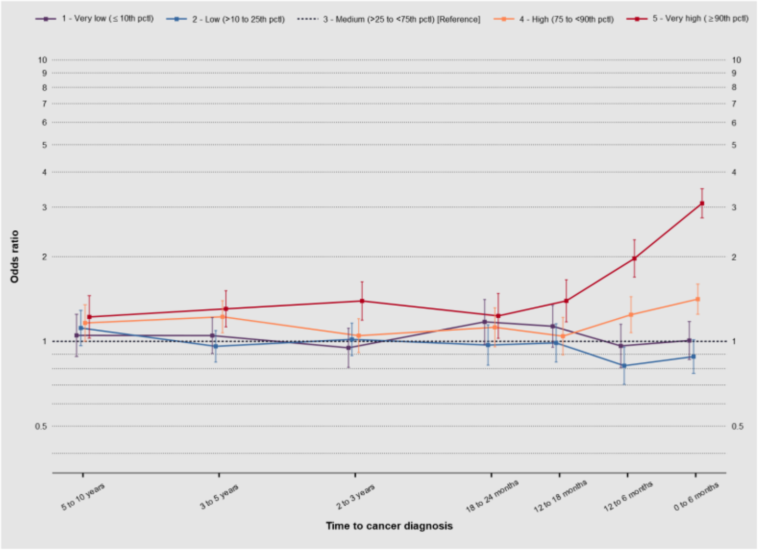

© 2022 Giannakeas V et al. *JAMA Network Open*.

Cervix (N = 3,493)

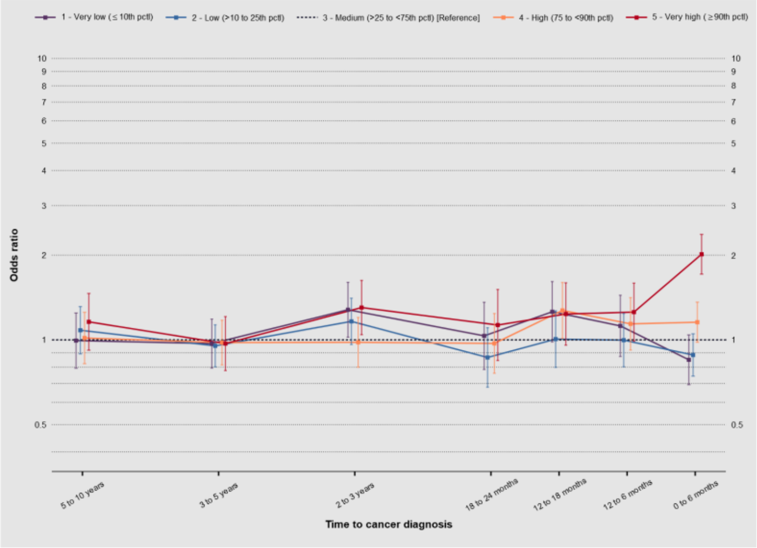

Endometrium (N = 17,124)

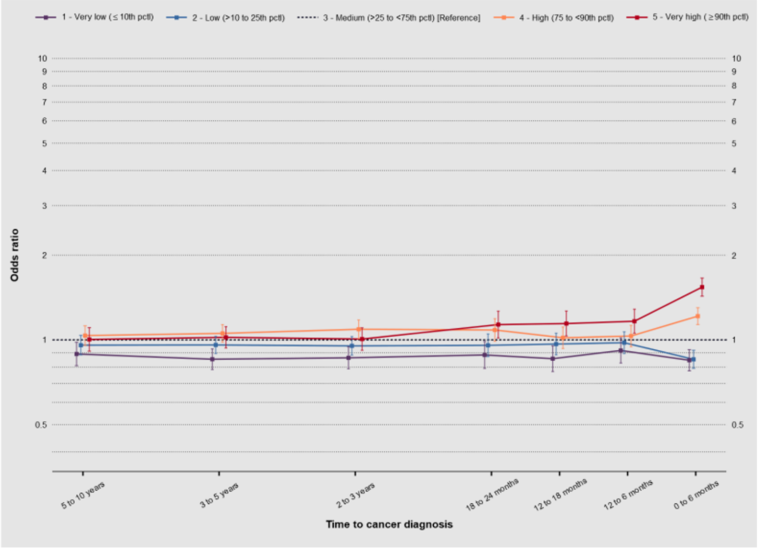

Bladder (N = 23,267)

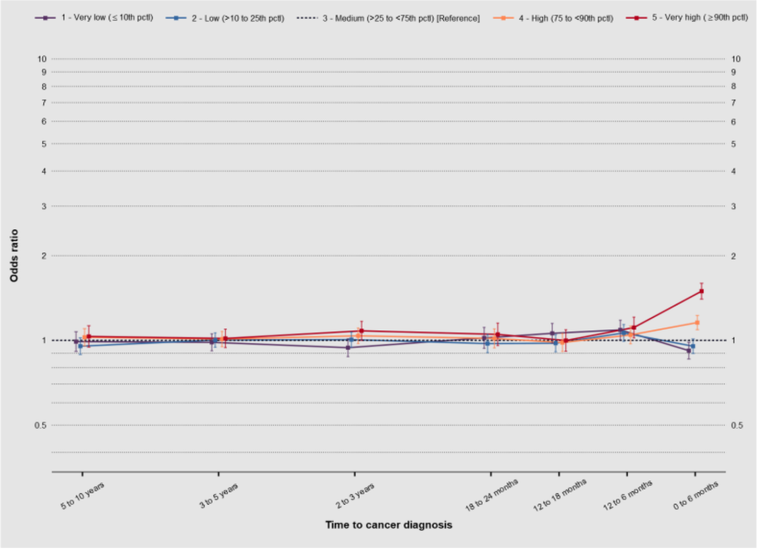

Liver (N = 7,651)

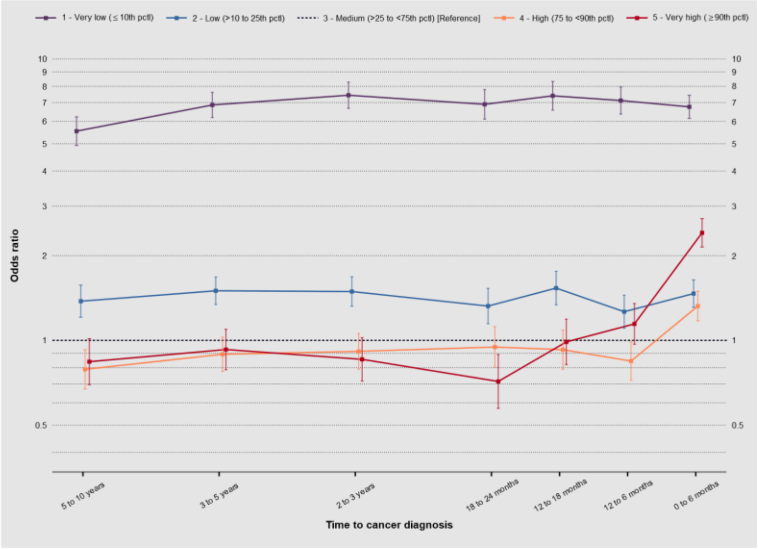

© 2022 Giannakeas V et al. *JAMA Network Open*.

Brain (N = 5,721)

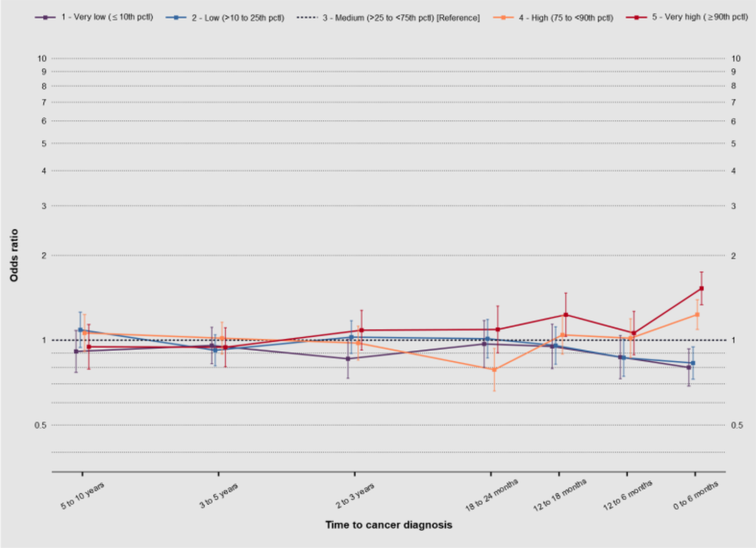

Head and neck (N = 13,318)

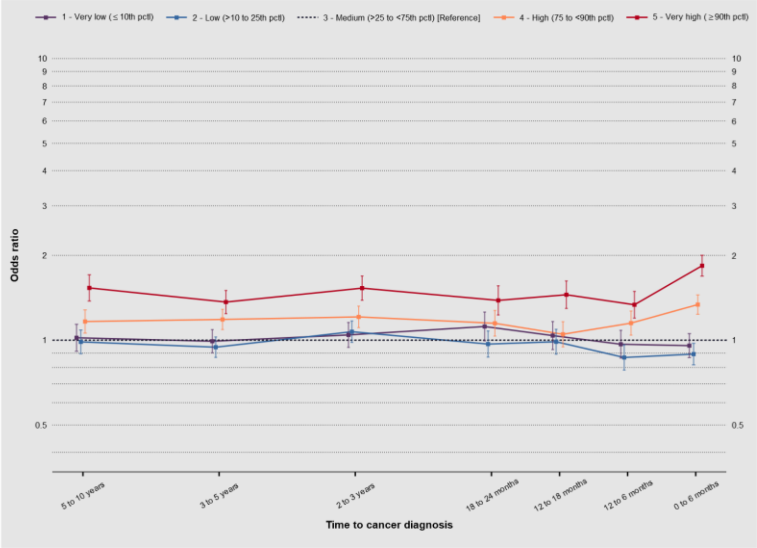

Other solid tumour (N = 26,907)

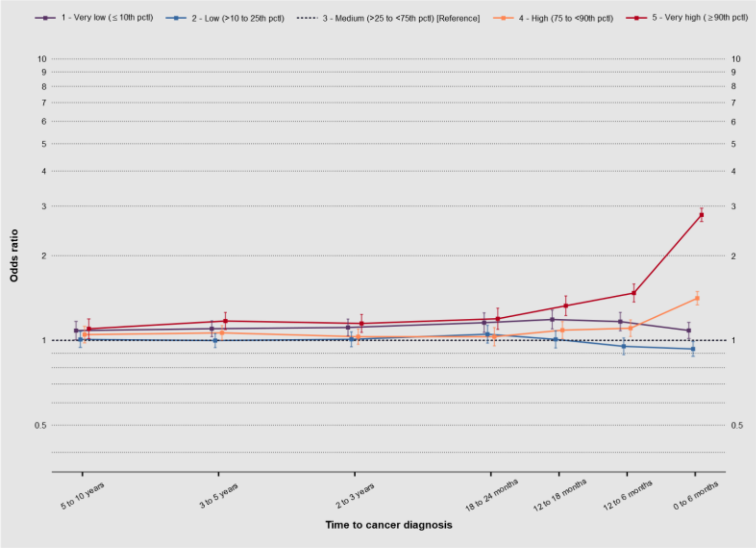

© 2022 Giannakeas V et al. *JAMA Network Open*.

Leukemia (N = 5,157)

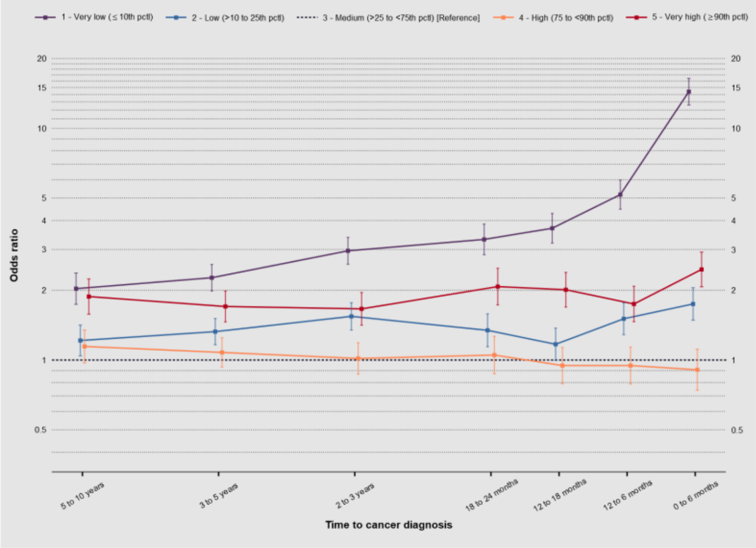

Lymphoma (N = 33,785)

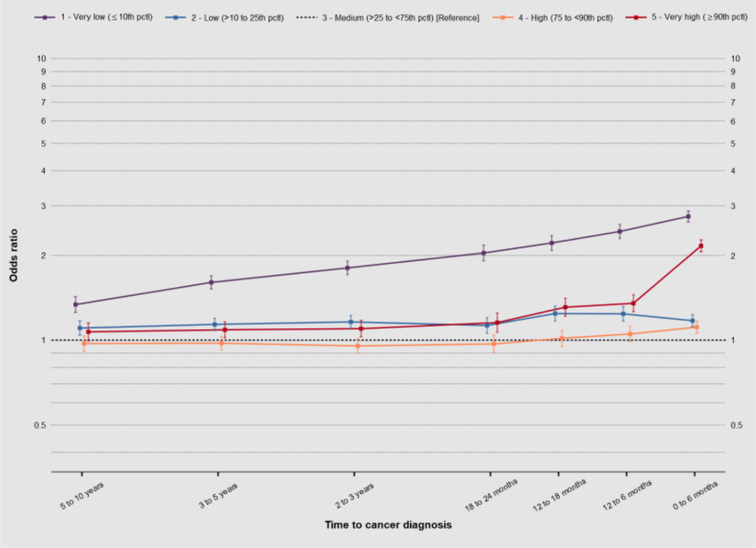

Multiple myeloma (N = 8,265)

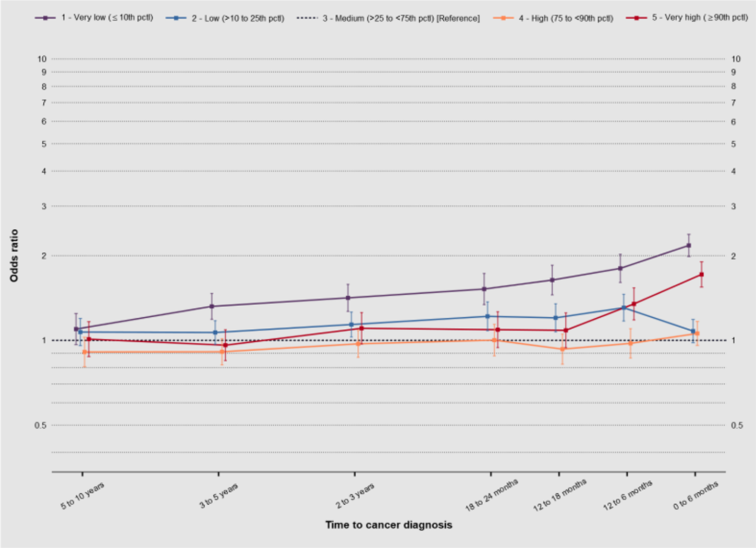

Other hematologic tumour (N = 16,195)

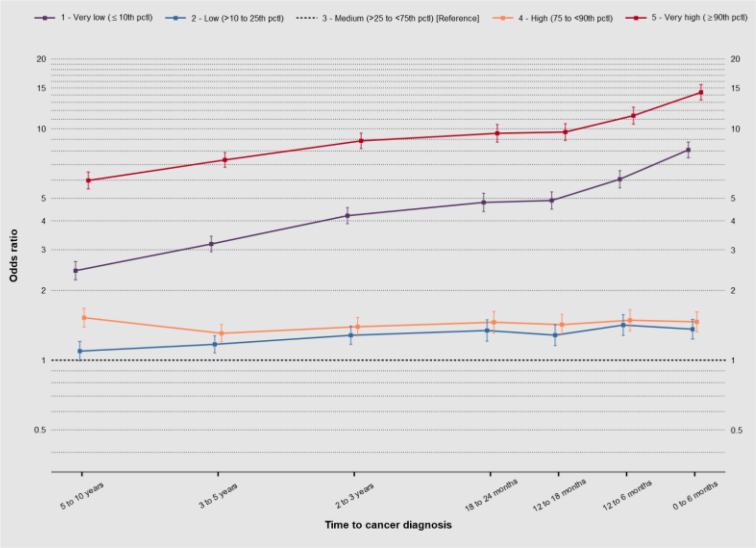

© 2022 Giannakeas V et al. *JAMA Network Open*.

eFigure 5. Odds ratio of cancer by change in platelet count category and time from cancer diagnosis. Additional cancer sites.

Breast (N = 32,650)

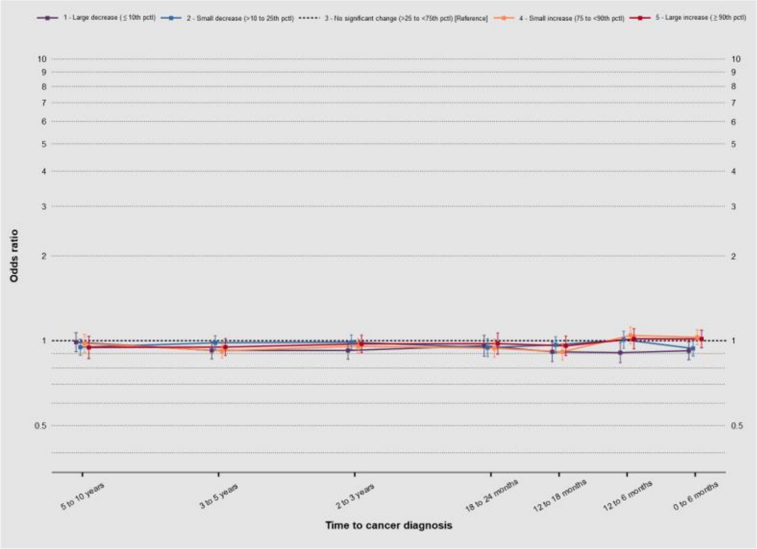

Prostate (N = 30,994)

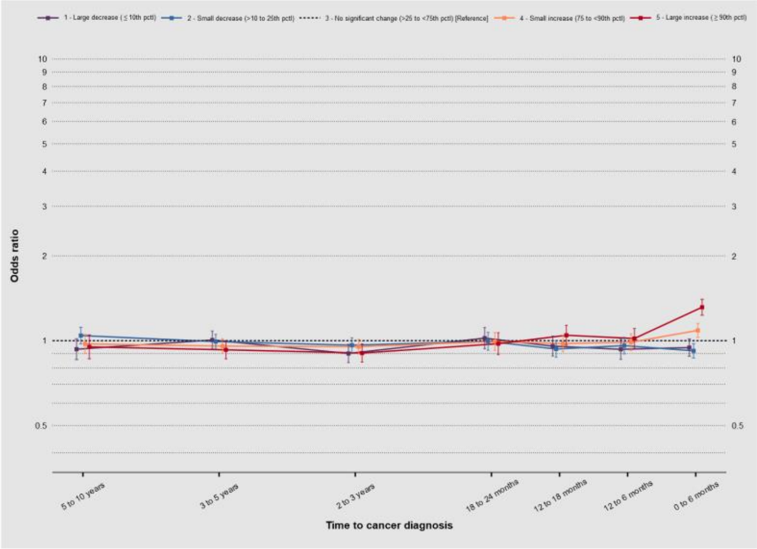

Melanoma (N = 9,957)

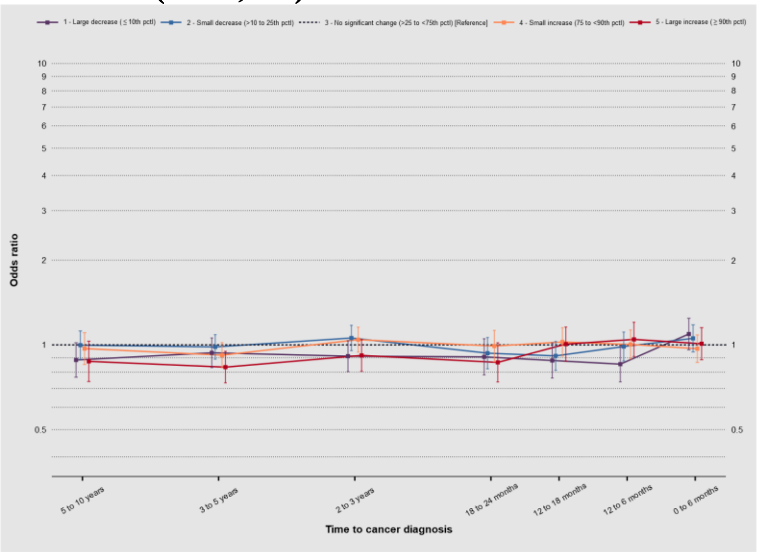

Thyroid (N = 10,176)

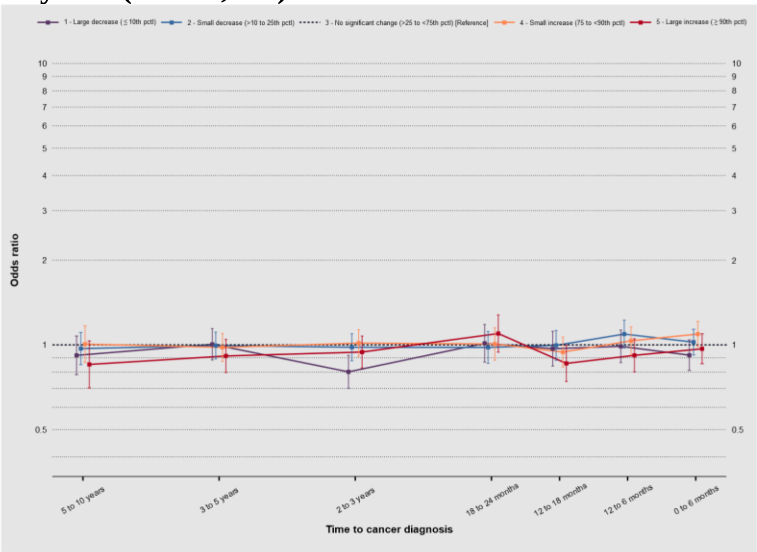

© 2022 Giannakeas V et al. *JAMA Network Open*.

Esophagus (N = 2,307)

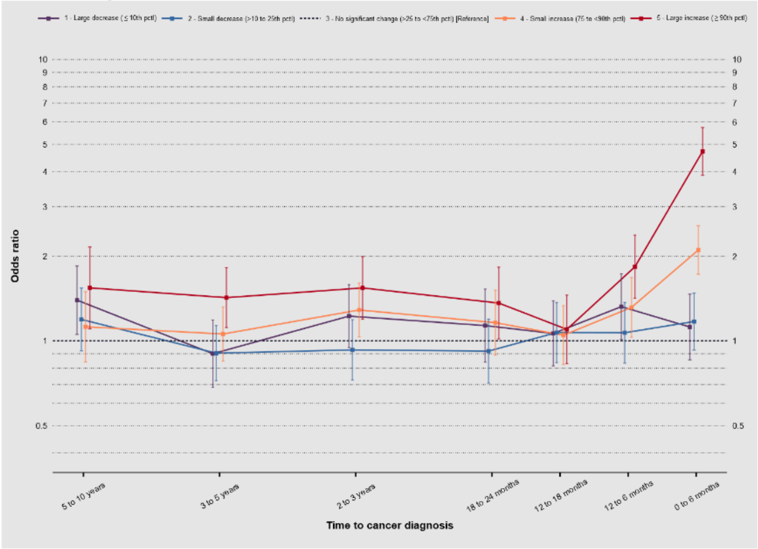

Kidney (N = 7,149)

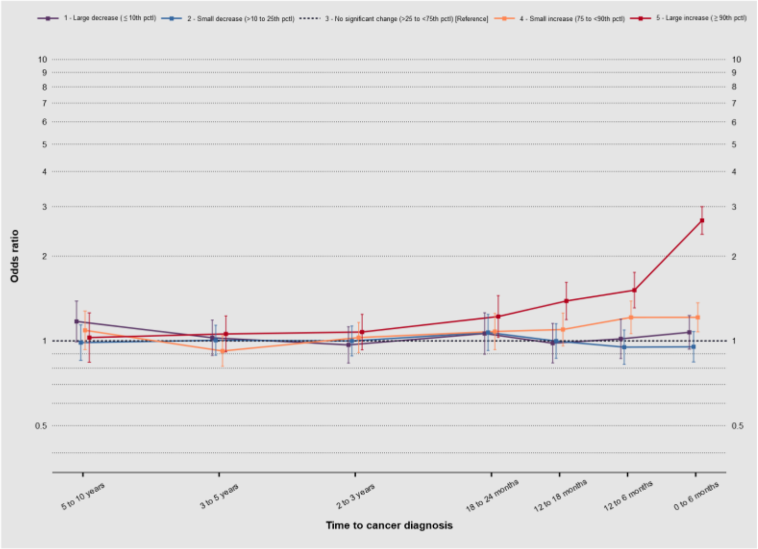

Pancreas (N = 6,667)

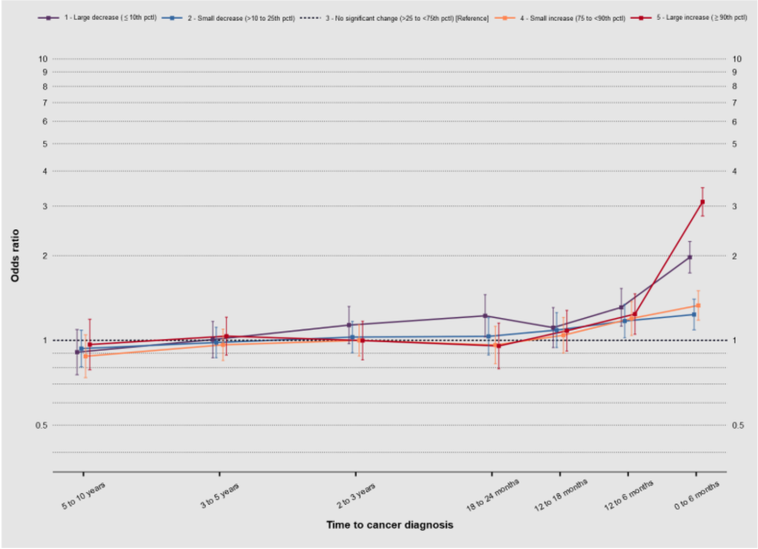

Other GI (N = 2,816)

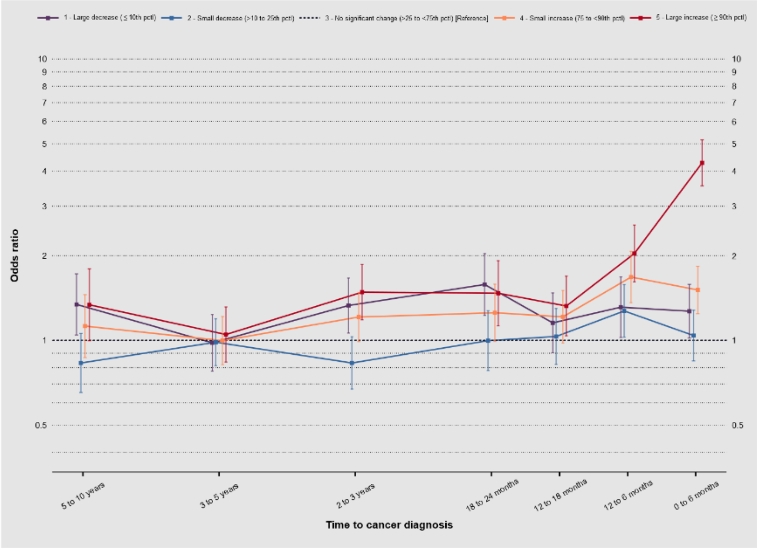

Cervix (N = 1,192)

Endometrium (N = 8,597)

© 2022 Giannakeas V et al. *JAMA Network Open*.

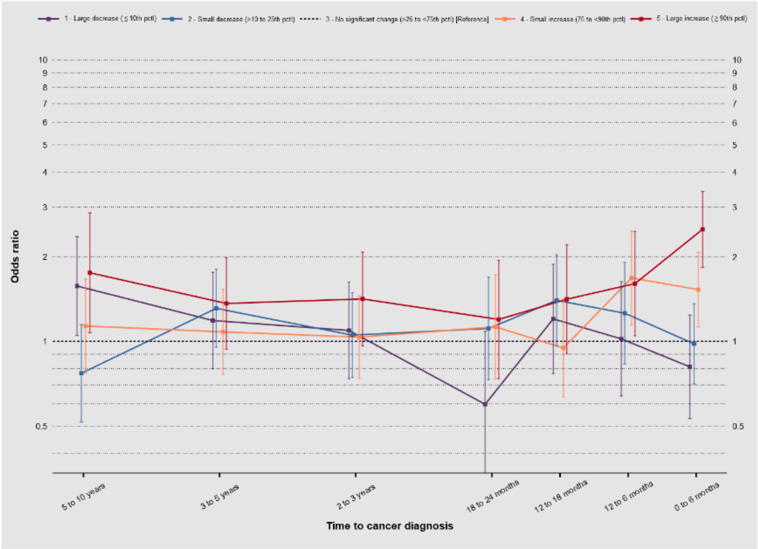

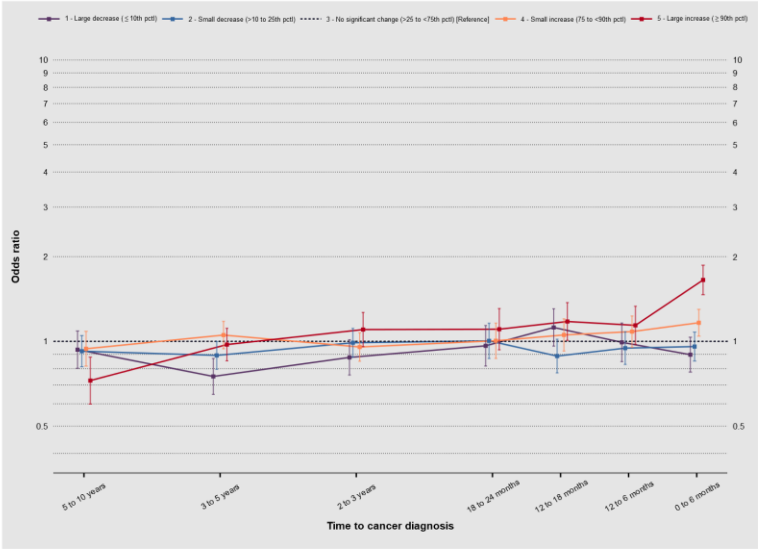

Bladder (N = 12,895)

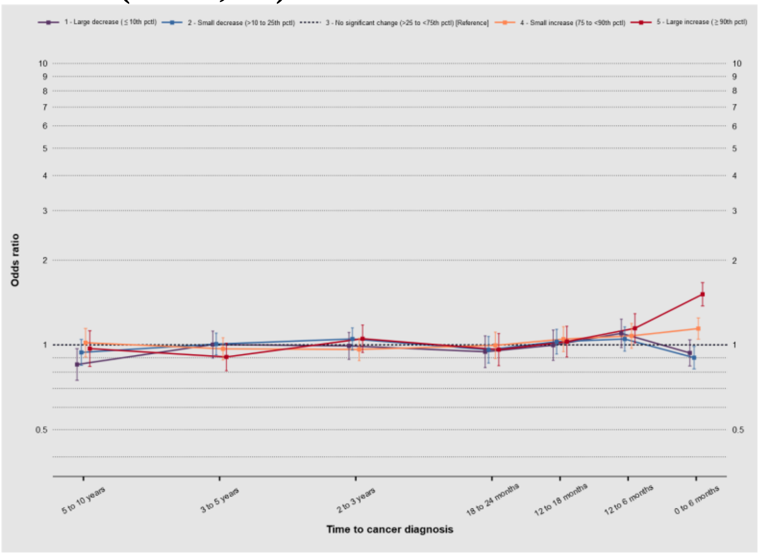

Liver (N = 4,489)

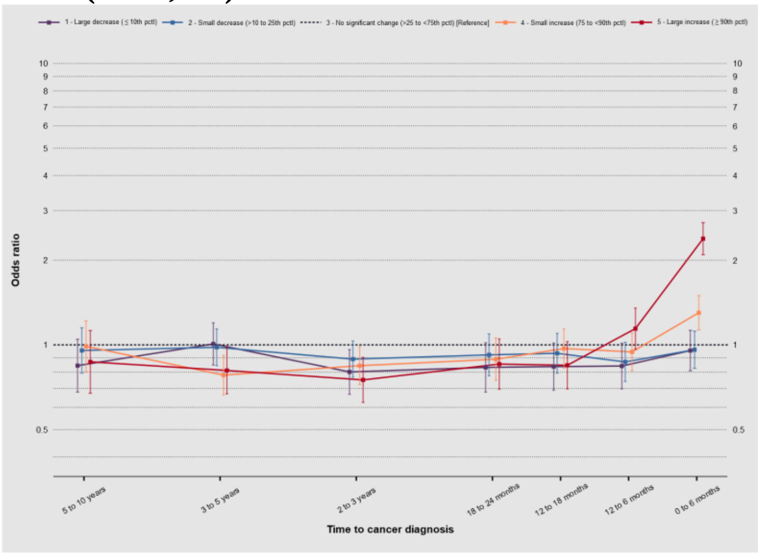

© 2022 Giannakeas V et al. *JAMA Network Open*.

Brain (N = 2,648)

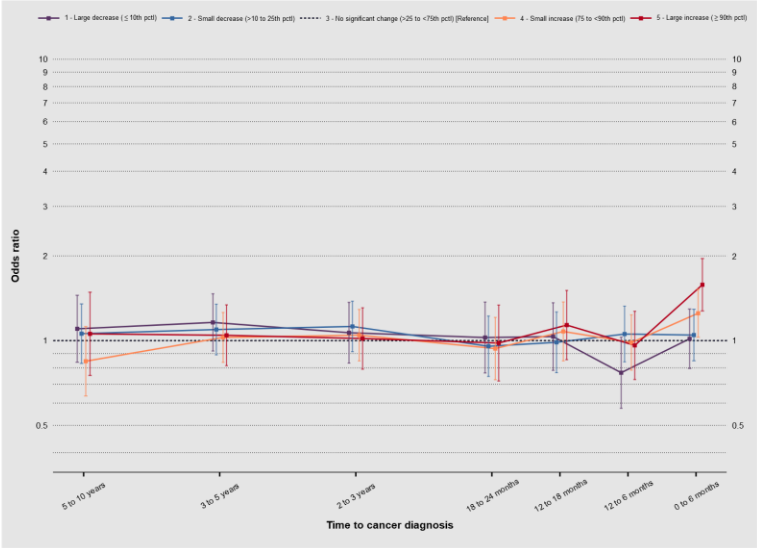

Head and neck (N = 6,393)

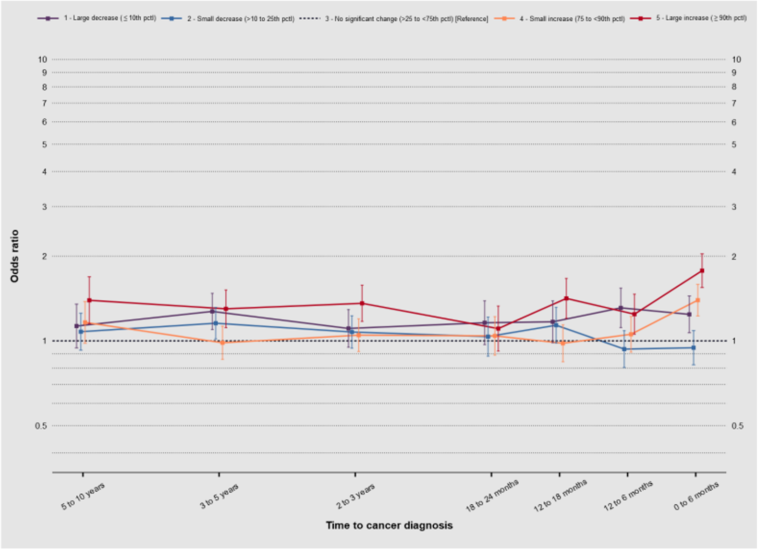

Other solid tumour (N = 13,624)

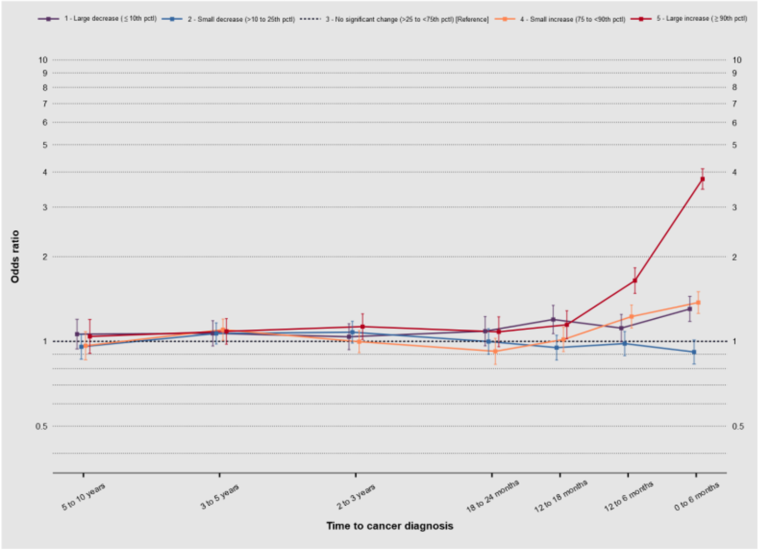

© 2022 Giannakeas V et al. *JAMA Network Open*.

Leukemia (N = 2,834)

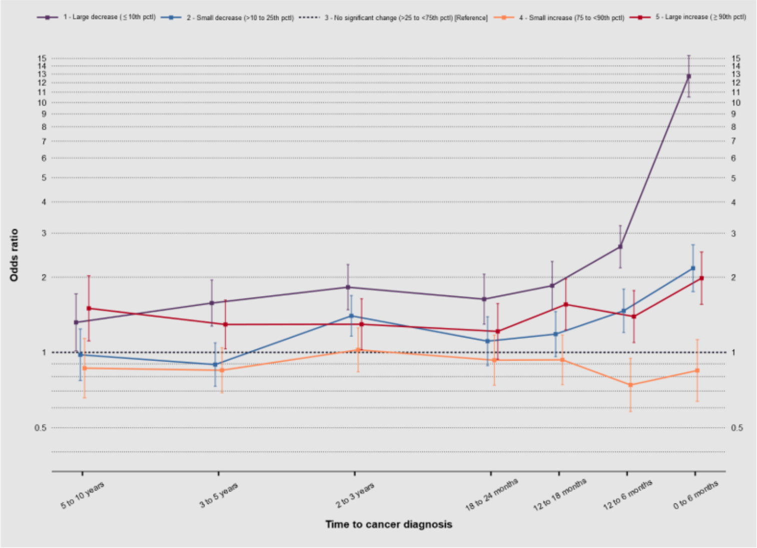

Lymphoma (N = 17,736)

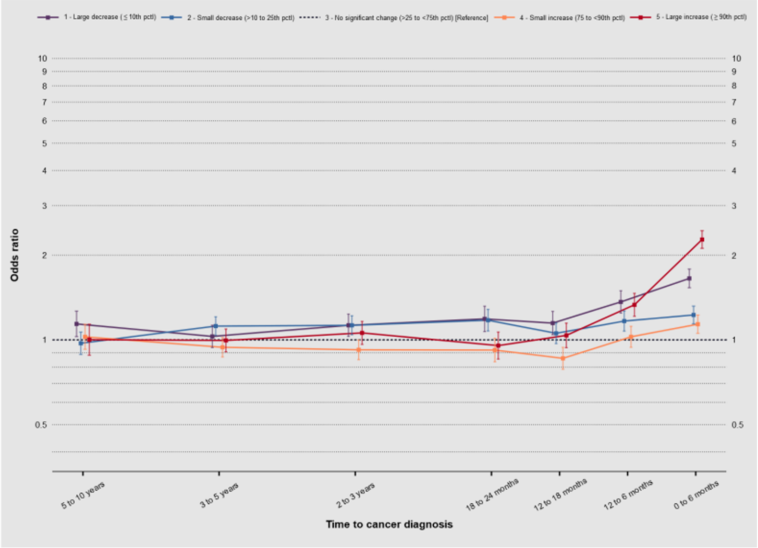

Multiple myeloma (N = 4,877)

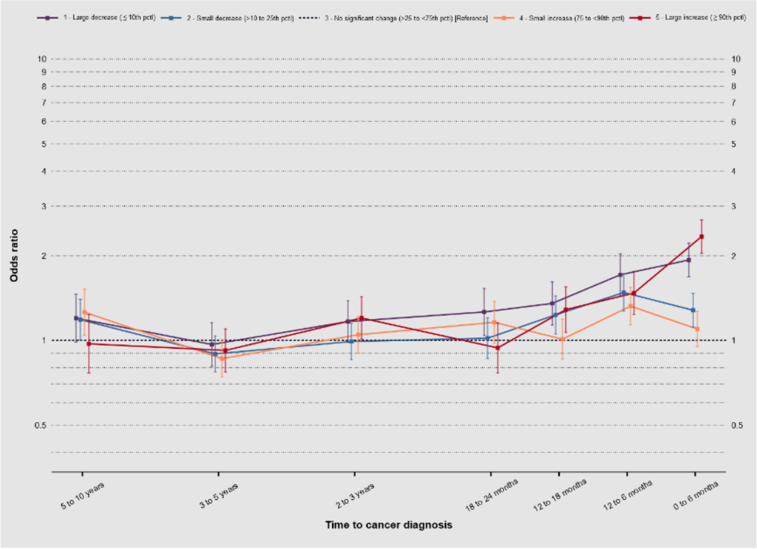

Other hematologic tumour (N = 10,306)

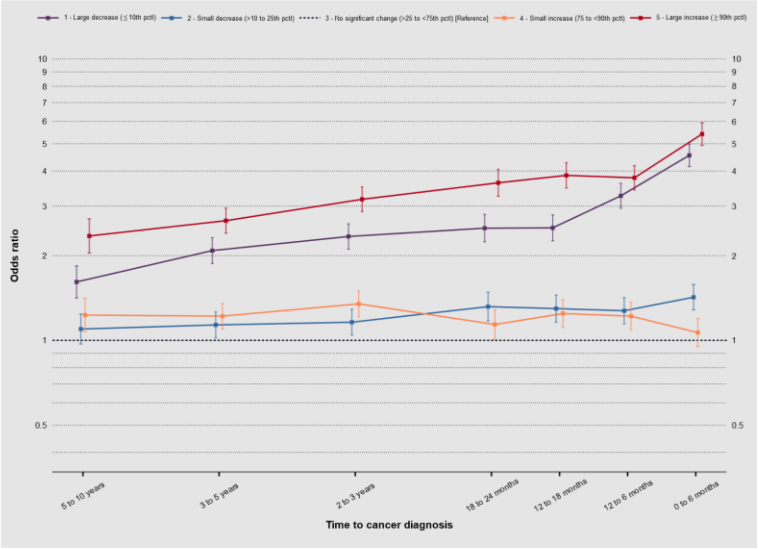

© 2022 Giannakeas V et al. *JAMA Network Open*.

eFigure 6. Odds ratio of cancer by platelet count category and time from diagnosis. Select cancer sites by cancer stage.

Lung stage I (N = 9,697)

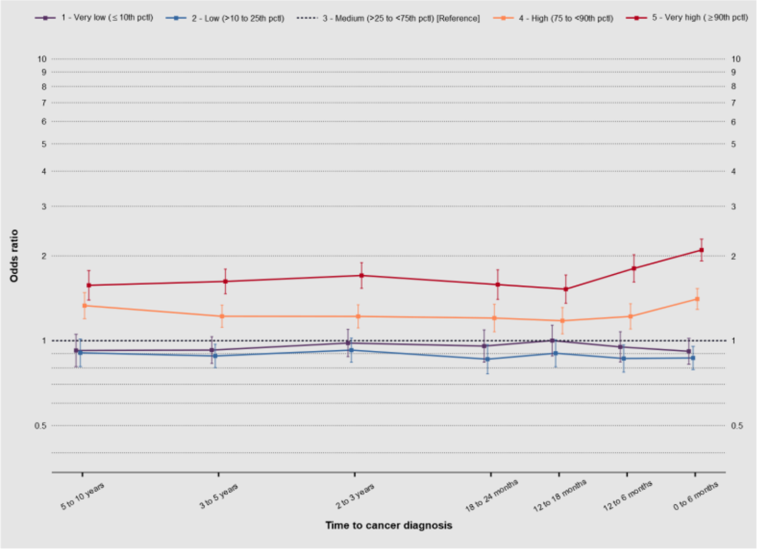

Lung stage II (N = 3,921)

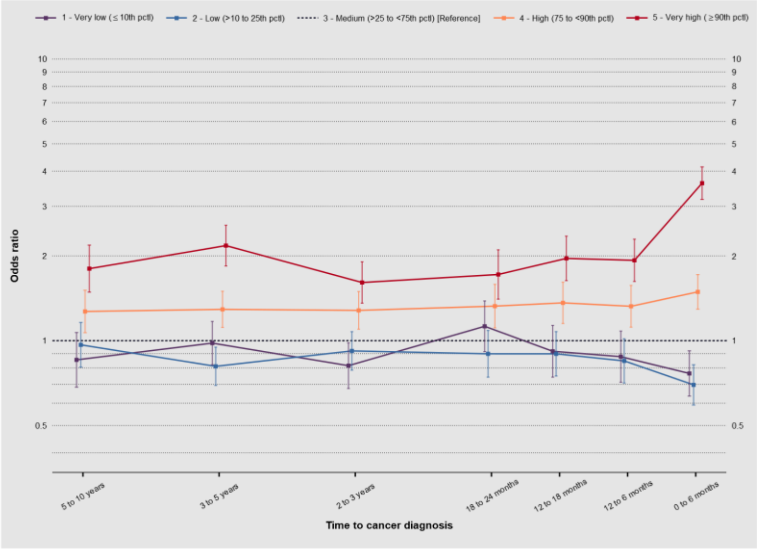

Lung stage III (N = 9,911)

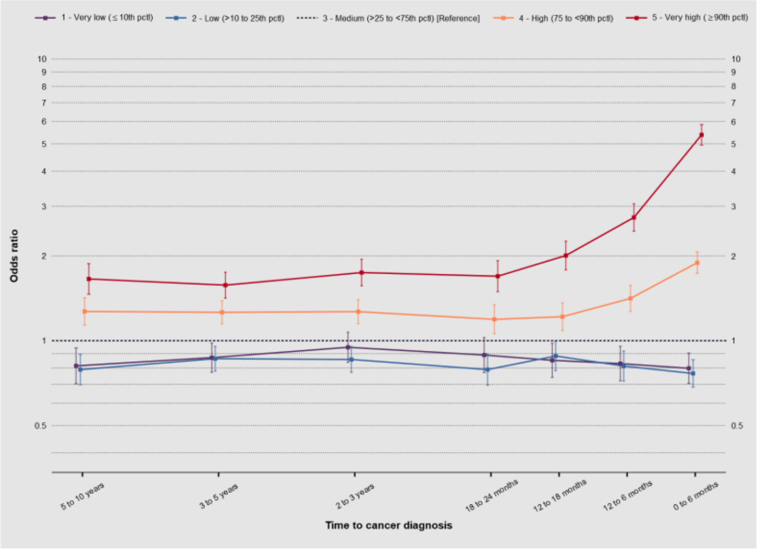

Lung stage IV (N = 24,390)

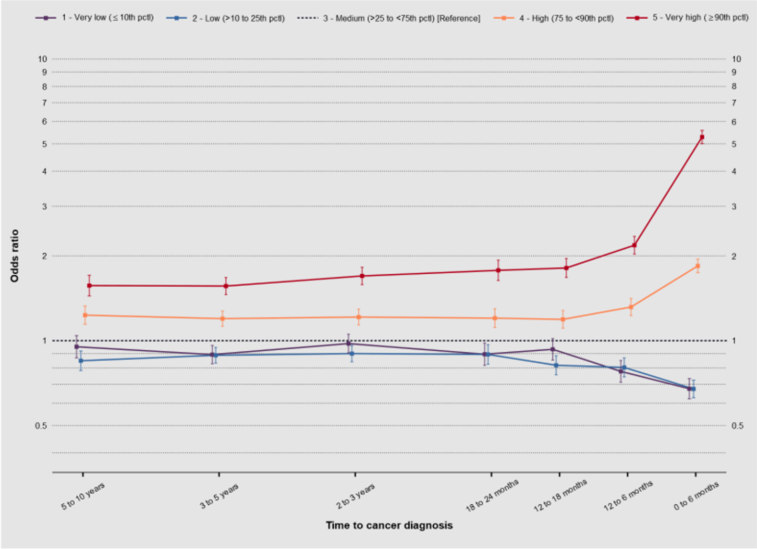

© 2022 Giannakeas V et al. *JAMA Network Open*.

Ovary stage I (N = 1,196)

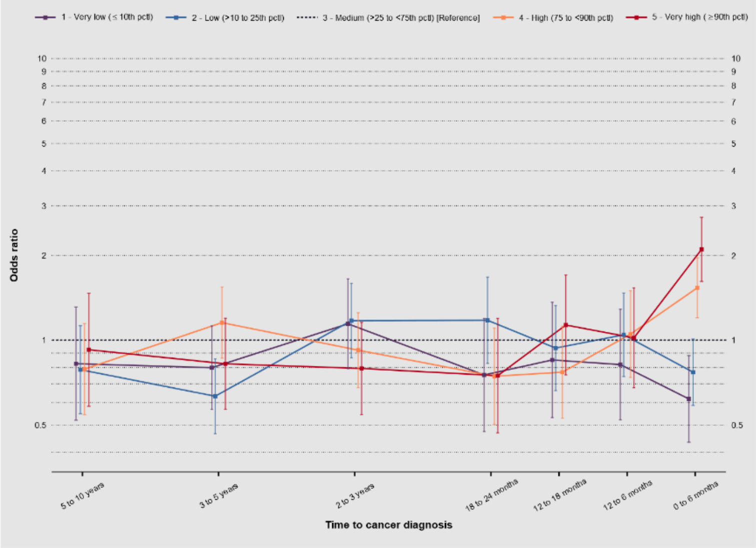

Ovary stage II (N = 543)

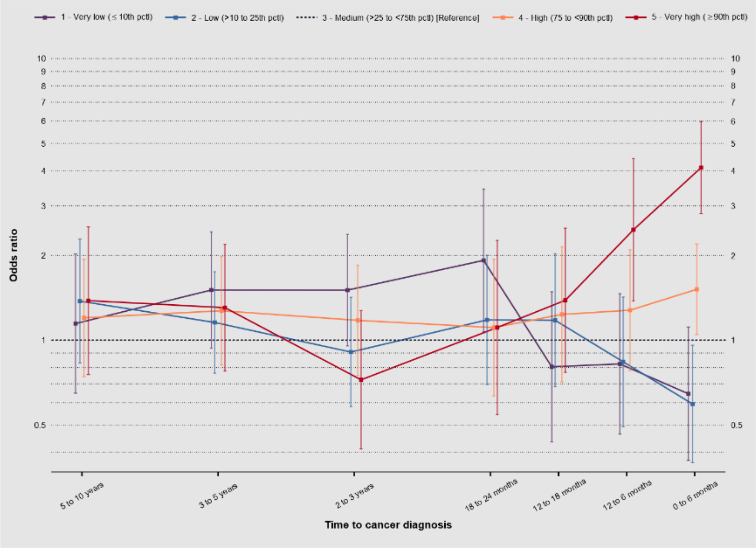

Ovary stage III (N = 2,315)

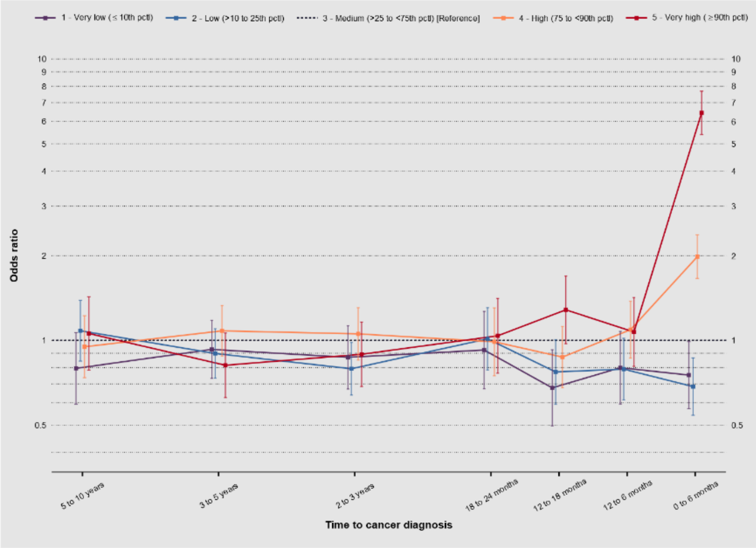

Ovary stage IV (N = 926)

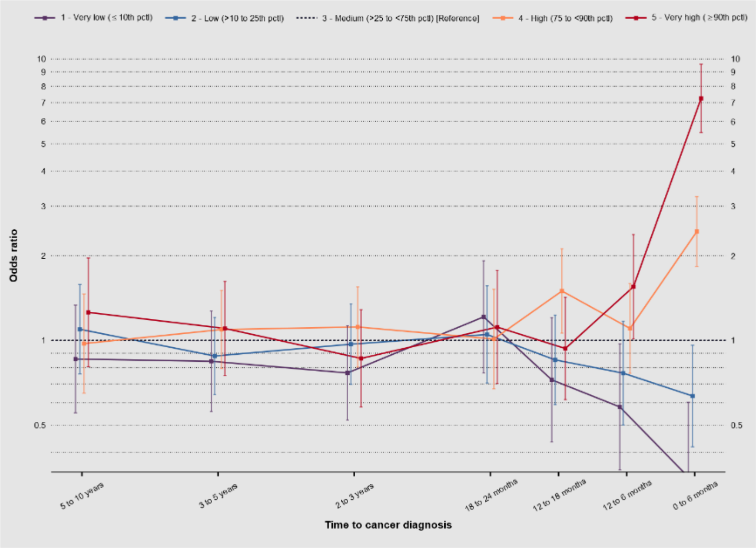

© 2022 Giannakeas V et al. *JAMA Network Open*.

Stomach stage I (N = 600)

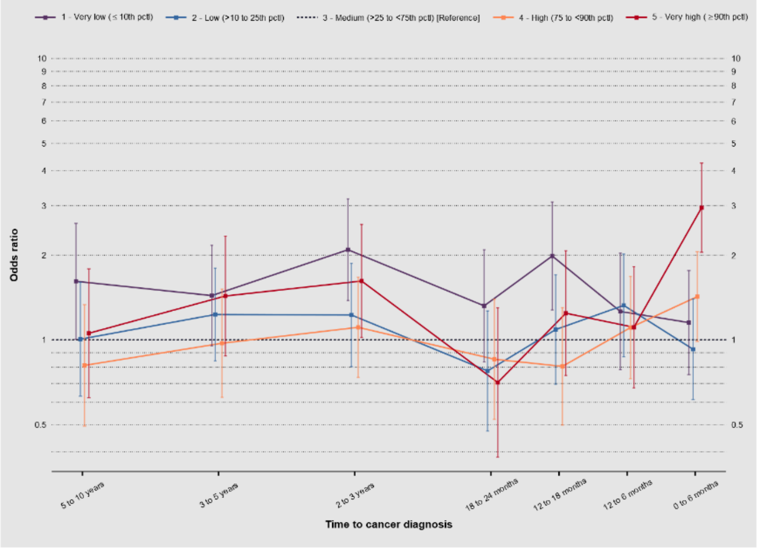

Stomach stage II (N = 678)

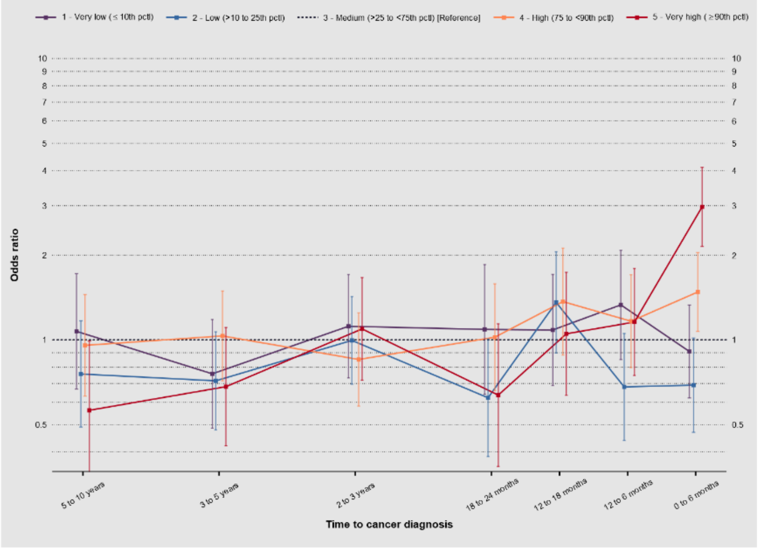

Stomach stage III (N = 799)

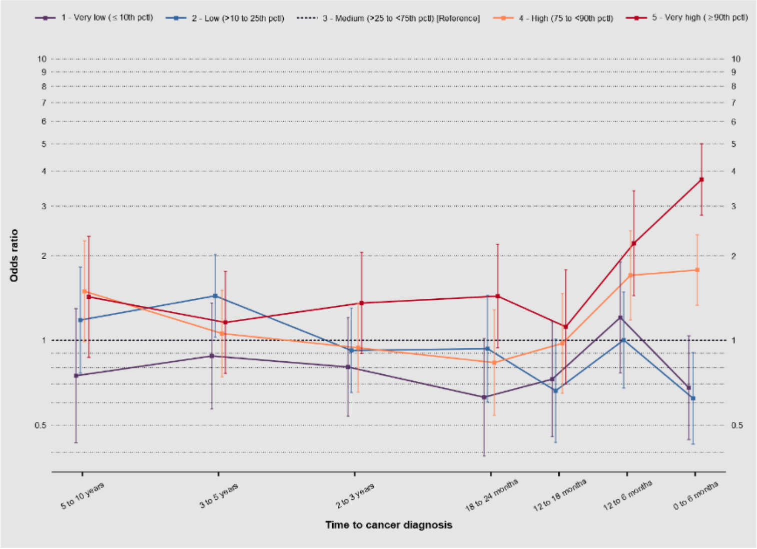

Stomach stage IV (N = 1,673)

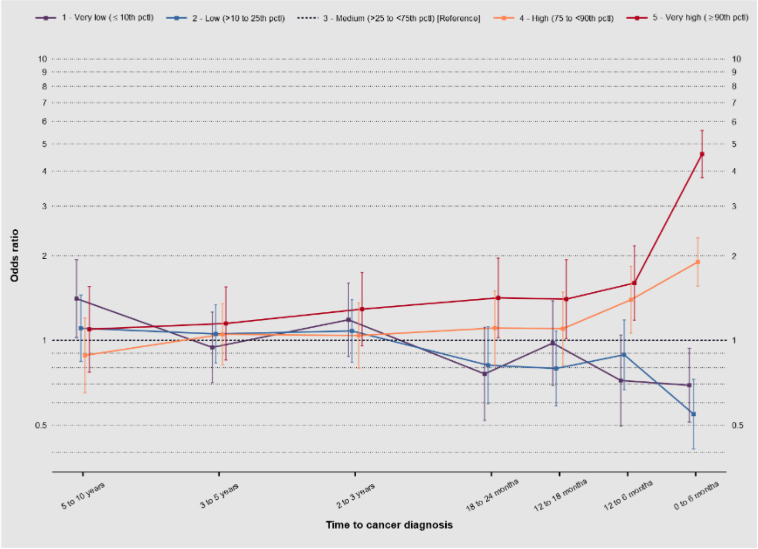

© 2022 Giannakeas V et al. *JAMA Network Open*.

Breast stage I (N = 28,646)

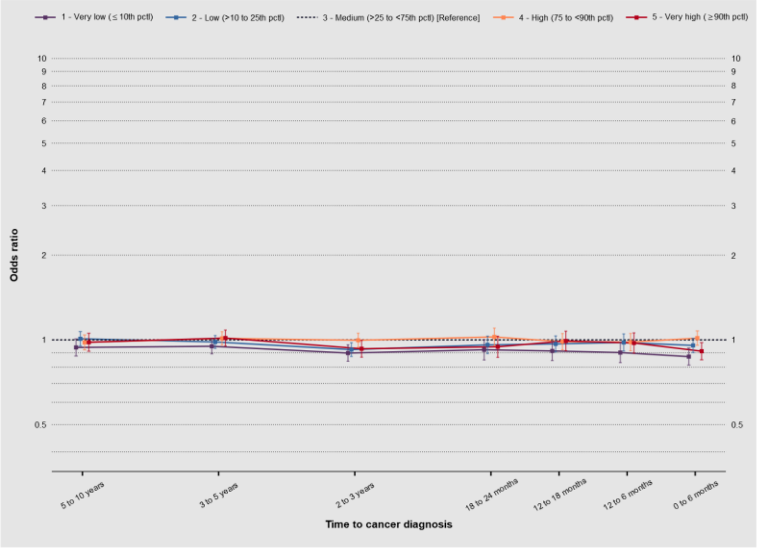

Breast stage II (N = 22,577)

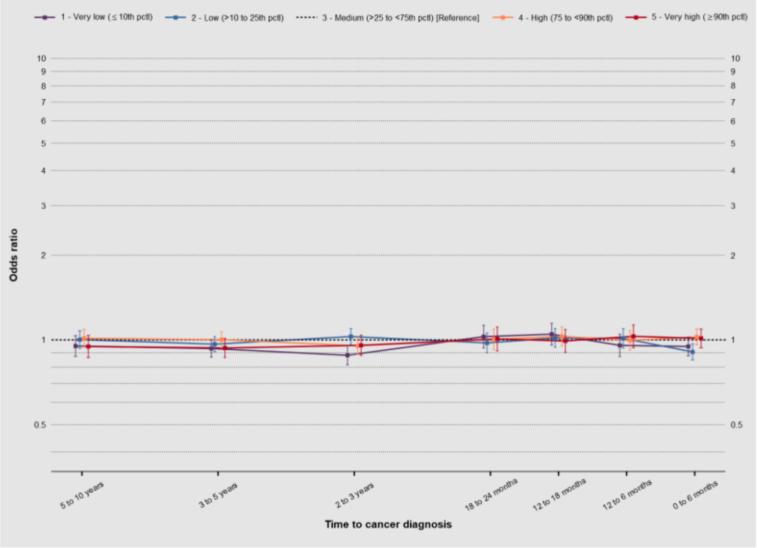

Breast stage III (N = 7,740)

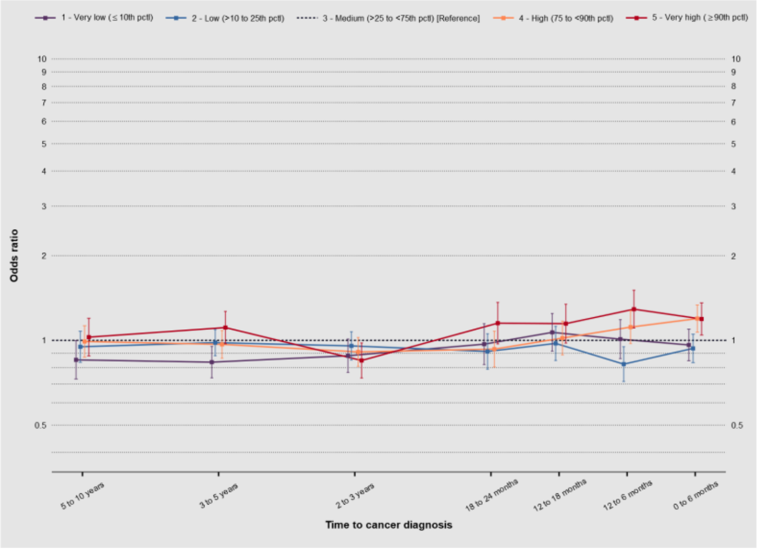

Breast stage IV (N = 2,672)

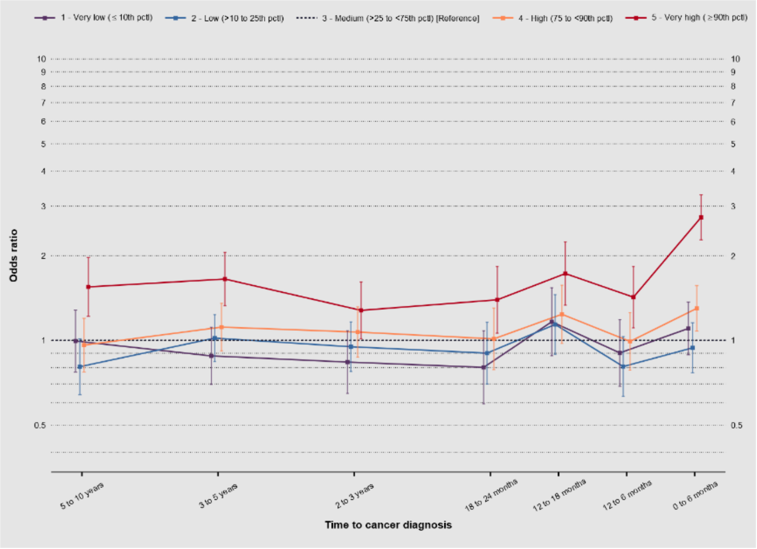

© 2022 Giannakeas V et al. *JAMA Network Open*.

Prostate stage I (N = 12,383)

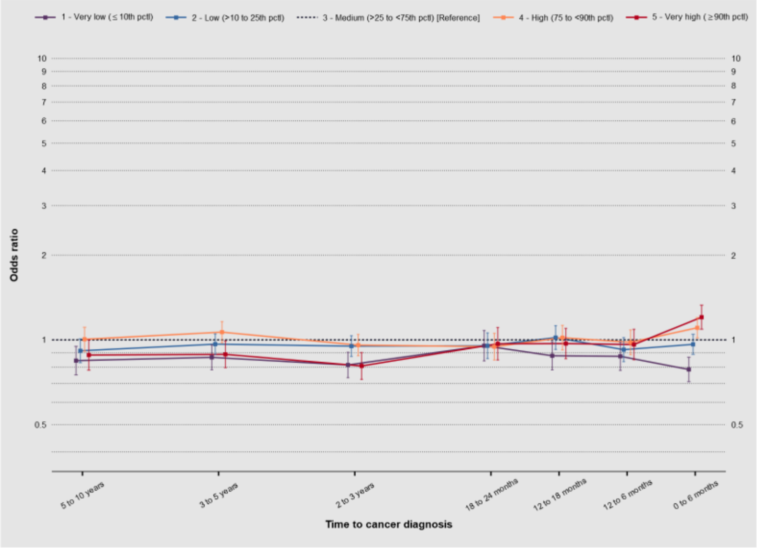

Prostate stage II (N = 30,688)

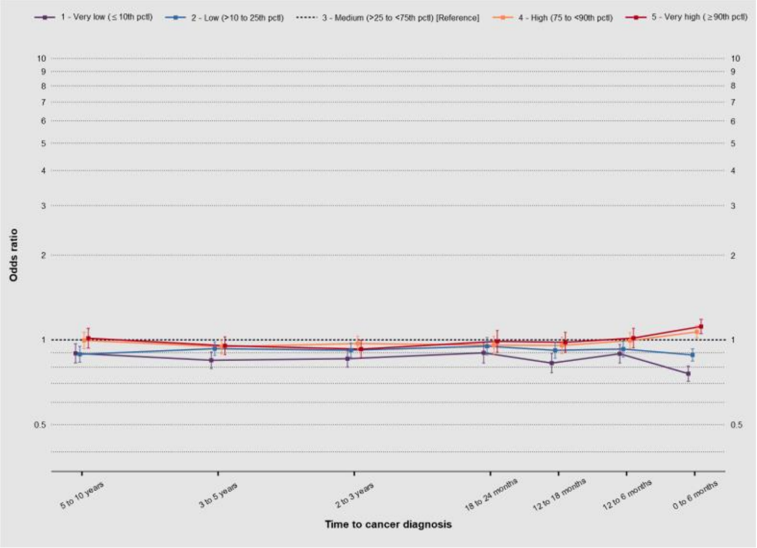

Prostate stage III (N = 8,137)

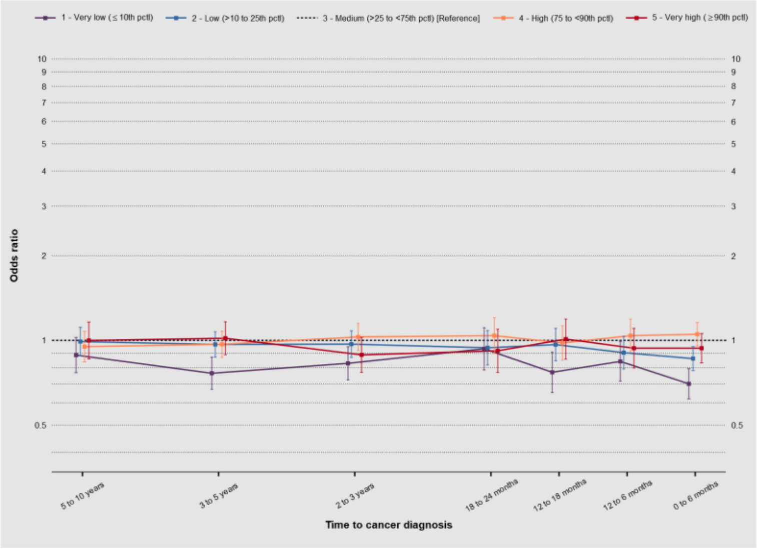

Prostate stage IV (N = 5,797)

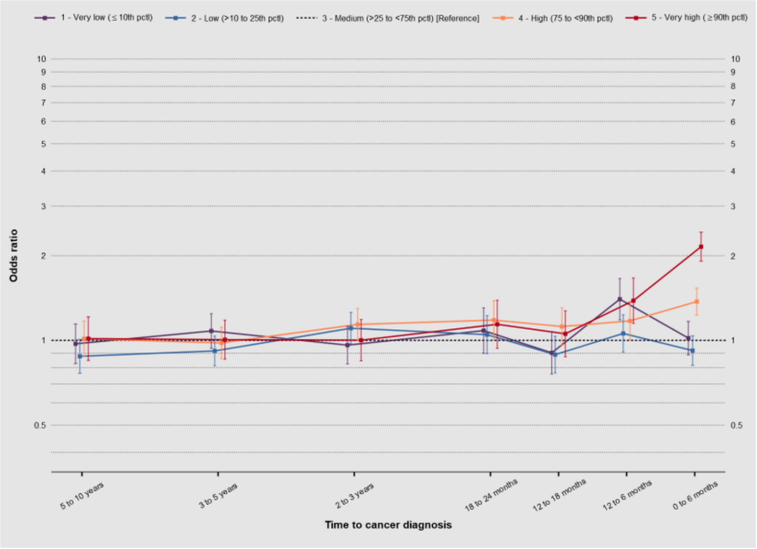

© 2022 Giannakeas V et al. *JAMA Network Open*.

eFigure 7. Odds ratio of cancer by platelet count category (clinical definition) and time from diagnosis.

Colon (N = 51,271)

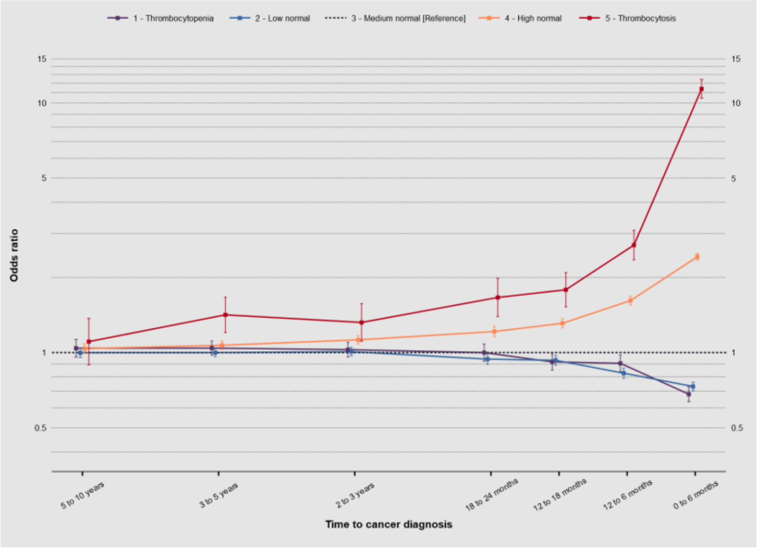

Lung (N = 56,586)

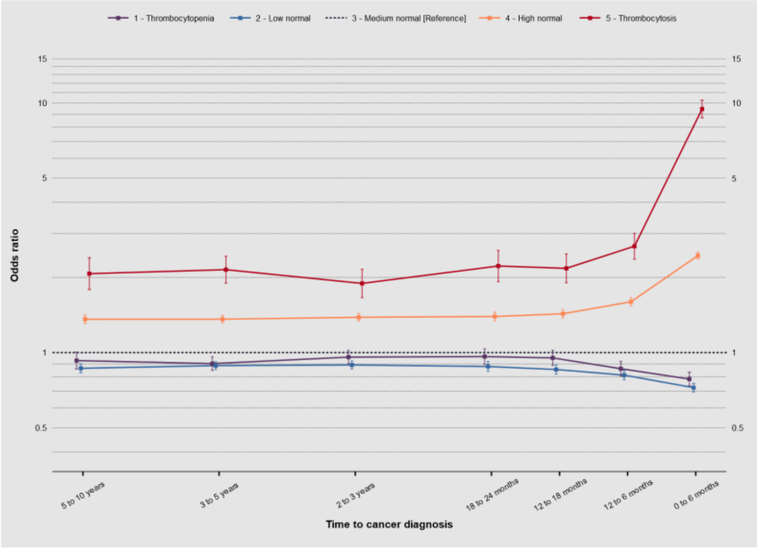

Ovary (N = 7,658)

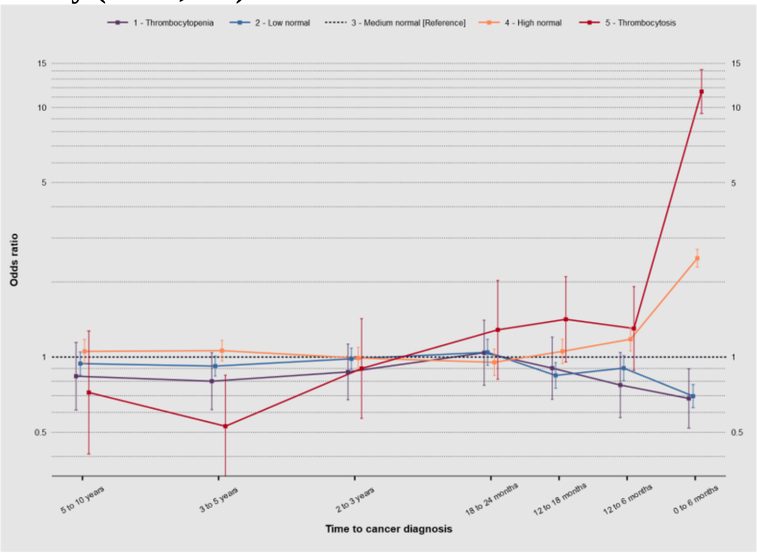

Stomach (N = 9,166)

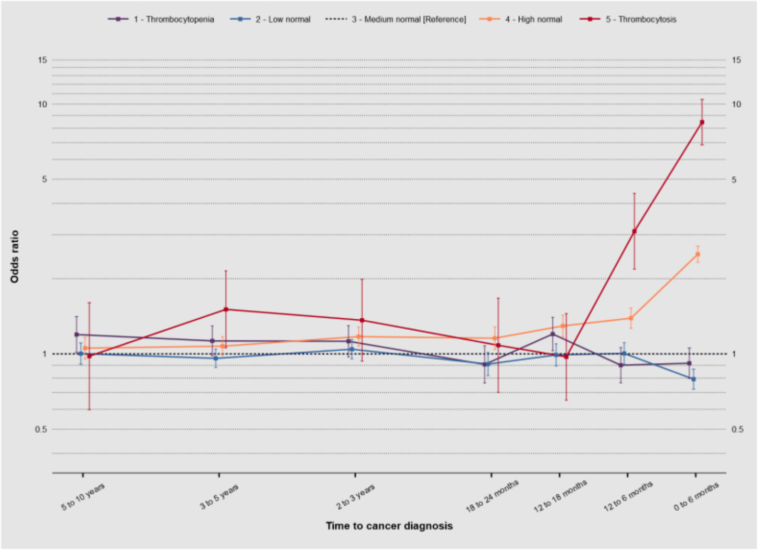

© 2022 Giannakeas V et al. *JAMA Network Open*.

Breast (N = 65,703)

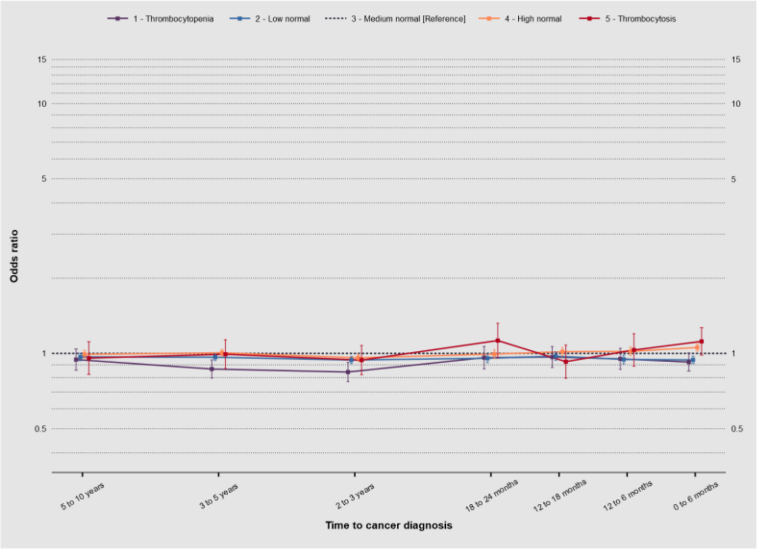

Prostate (N = 62,770)

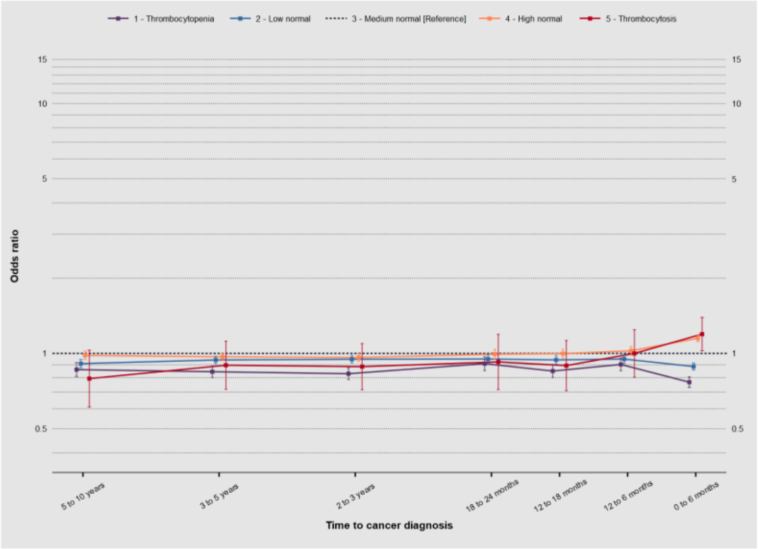

Melanoma (N = 20,167)

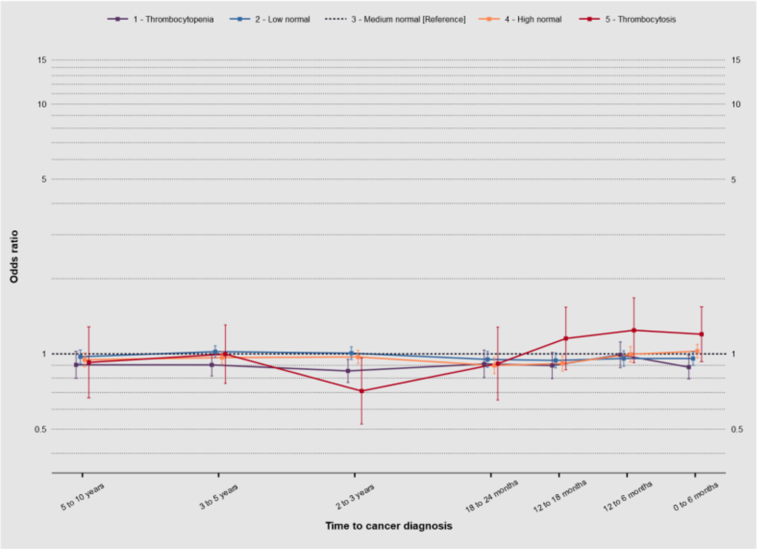

Thyroid (N = 21,559)

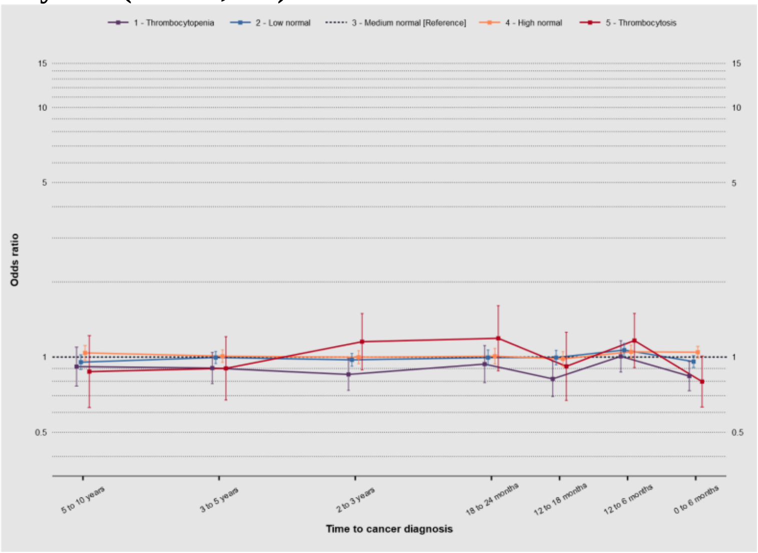

© 2022 Giannakeas V et al. *JAMA Network Open*.

Esophagus (N = 4,691)

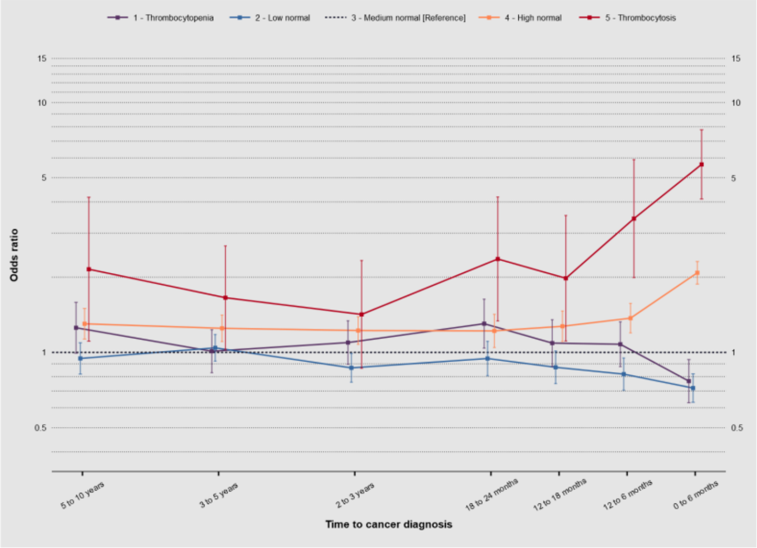

Kidney (N = 14,057)

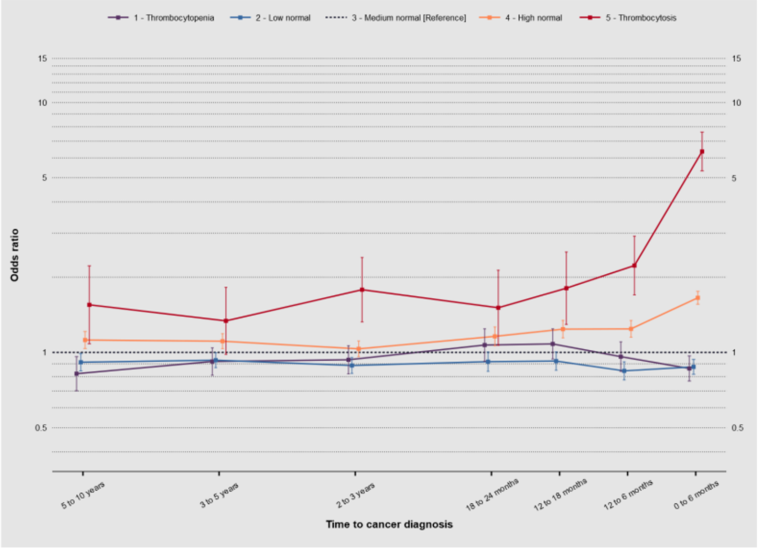

Pancreas (N = 12,009)

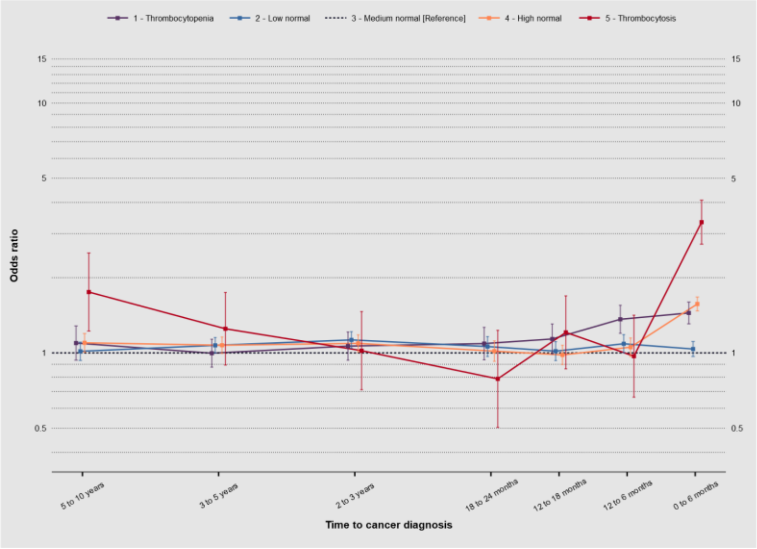

Other GI (N = 5,259)

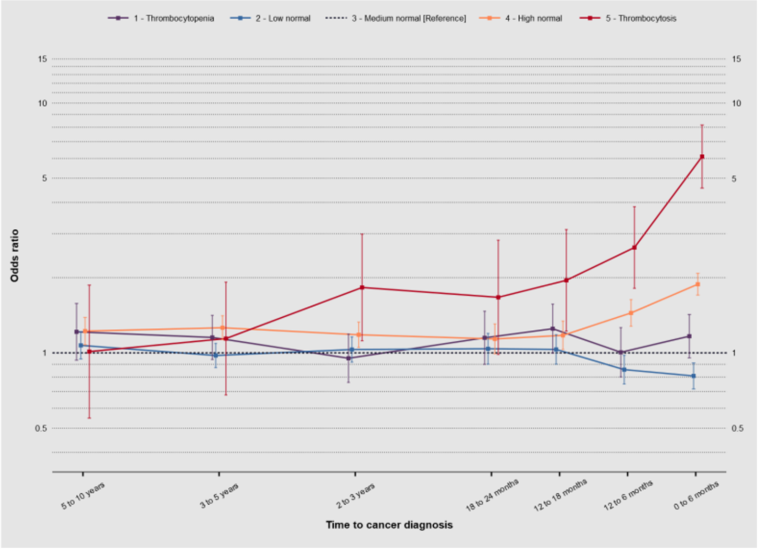

© 2022 Giannakeas V et al. *JAMA Network Open*.

Cervix (N = 3,493)

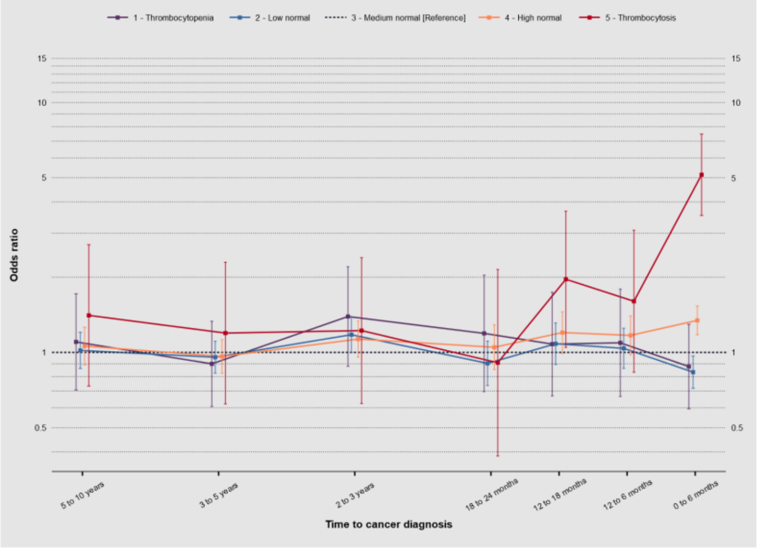

Endometrium (N = 17,124)

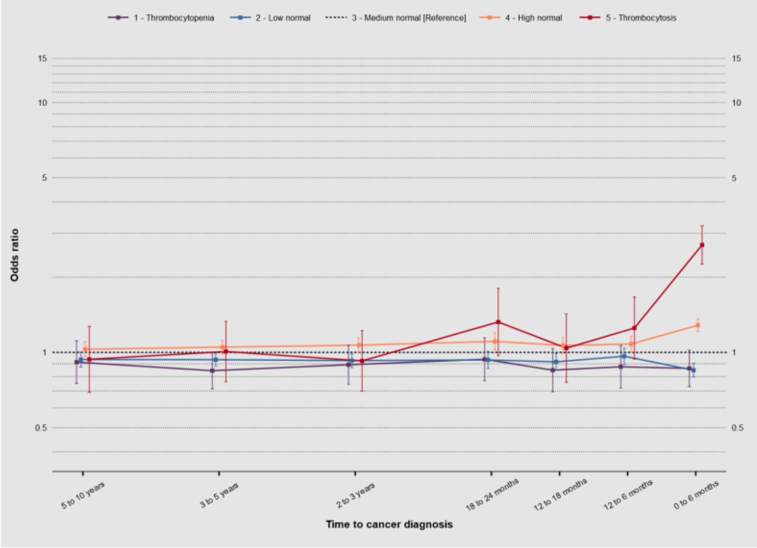

Bladder (N = 23,267)

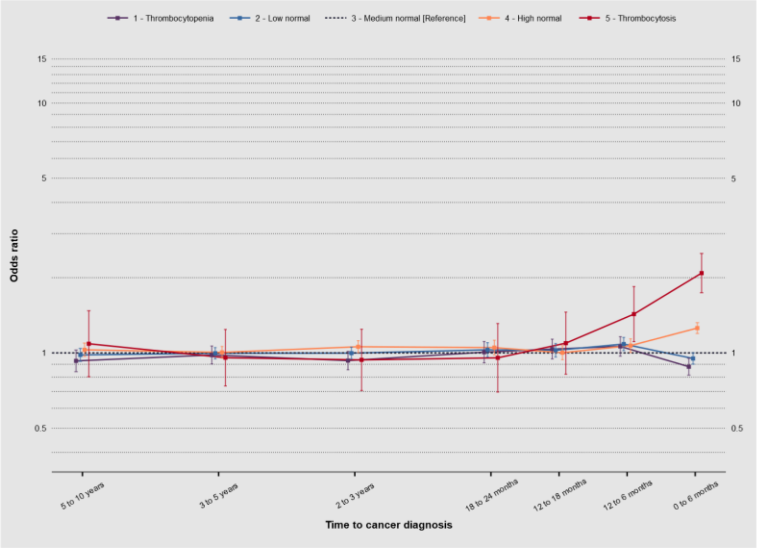

Liver (N = 7,651)

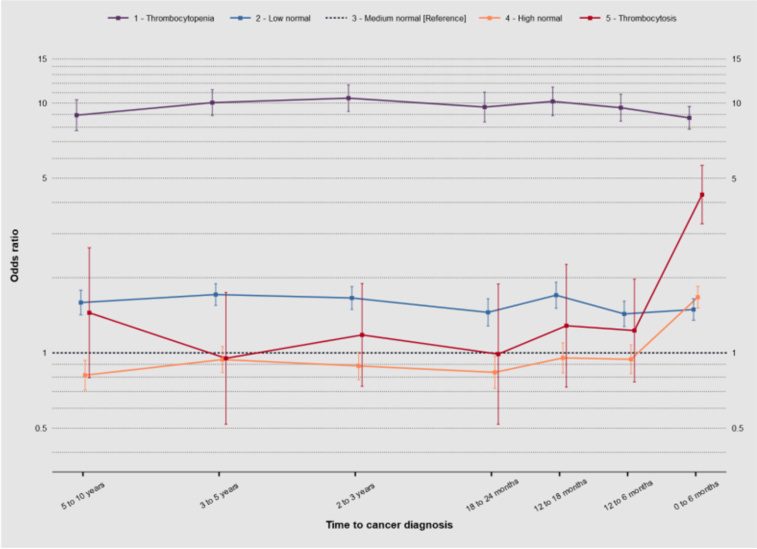

© 2022 Giannakeas V et al. *JAMA Network Open*.

Brain (N = 5,721)

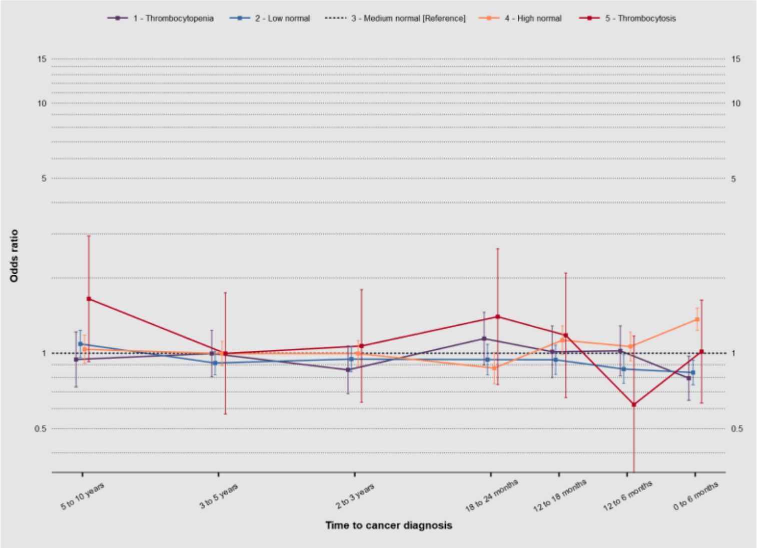

Head and neck (N = 13,318)

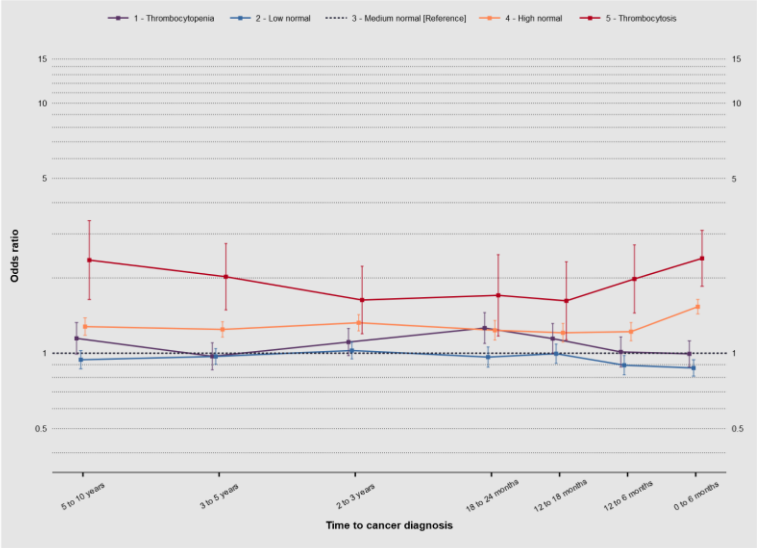

Other solid tumour (N = 26,907)

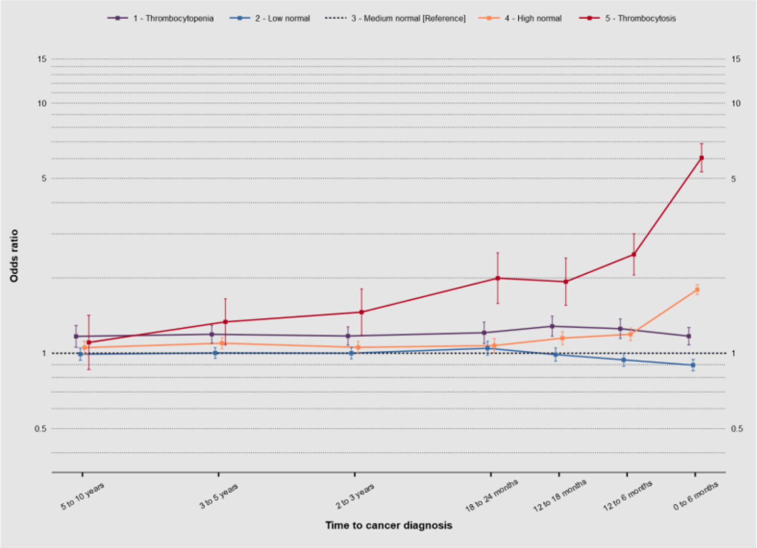

© 2022 Giannakeas V et al. *JAMA Network Open*.

Leukemia (N = 5,157)

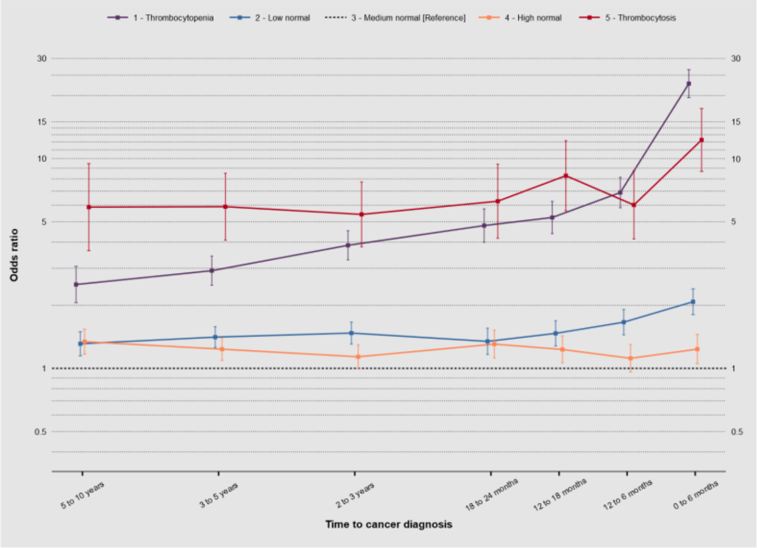

Lymphoma (N = 33,785)

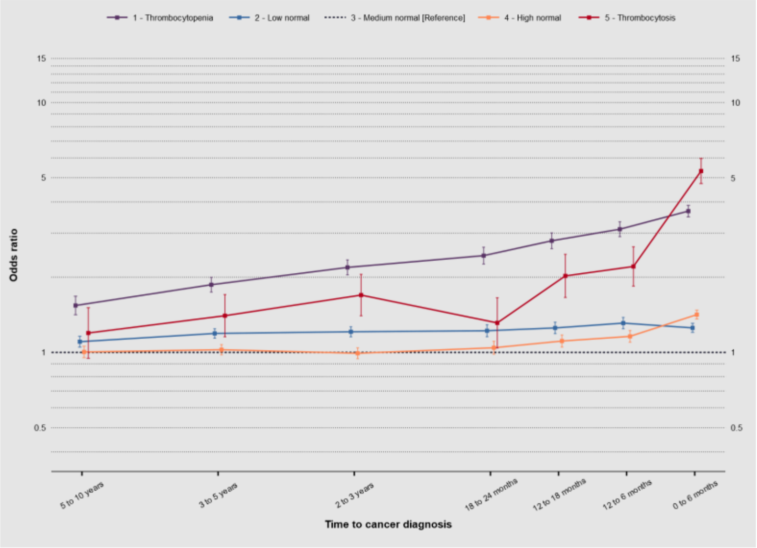

Multiple myeloma (N = 8,265)

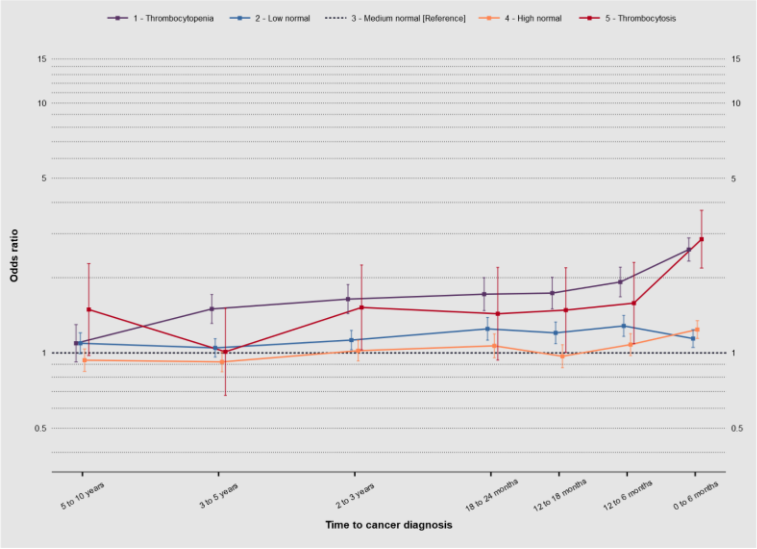

Other hematologic tumour (N = 16,195)

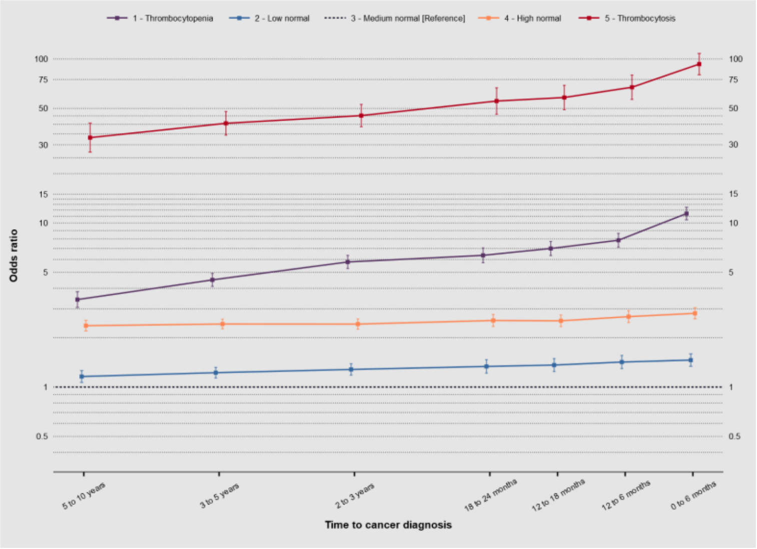

© 2022 Giannakeas V et al. *JAMA Network Open*.
